# Identification of ARF genes in *Cucurbita pepo L* and analysis of expression patterns, and functional analysis of *CpARF22* under drought, salt stress

**DOI:** 10.1186/s12864-024-09992-8

**Published:** 2024-01-25

**Authors:** Ming-jun Zhang, Ying-yu Xue, Shuang Xu, Xuan-ru Jin, Xing-chu Man

**Affiliations:** 1 College of Plant Protection, Gansu Agricultural University, Lanzhou, 730070 China; 2 Biocontrol Engineering Laboratory of Crop Diseases and Pests of Gansu Province, Gansu Agricultural University, Lanzhou, 730070 China

**Keywords:** Zucchini, ARF gene family, Drought and Salt stress, Pathogen stress, Transgenic *Arabidopsis thaliana*

## Abstract

**Background:**

Auxin transcription factor (ARF) is an important transcription factor that transmits auxin signals and is involved in plant growth and development as well as stress response. However, genome-wide identification and responses to abiotic and pathogen stresses of the ARF gene family in *Cucurbita pepo *L, especially pathogen stresses, have not been reported.

**Results:**

Finally, 33 ARF genes (*CpARF01* to *CpARF33*) were identified in* C.pepo *from the *Cucurbitaceae *genome database using bioinformatics methods. The putative protein contains 438 to 1071 amino acids, the isoelectric point is 4.99 to 8.54, and the molecular weight is 47759.36 to 117813.27 Da, the instability index ranged from 40.74 to 68.94, and the liposoluble index ranged from 62.56 to 76.18. The 33 genes were mainly localized in the nucleus and cytoplasm, and distributed on 16 chromosomes unevenly. Phylogenetic analysis showed that 33 CpARF proteins were divided into 6 groups. According to the amino acid sequence of CpARF proteins, 10 motifs were identified, and 1,3,6,8,10 motifs were highly conserved in most of the CpARF proteins. At the same time, it was found that genes in the same subfamily have similar gene structures. Cis-elements and protein interaction networks predicted that *CpARF* may be involved in abiotic factors related to the stress response. QRT-PCR analysis showed that most of the *CpARF* genes were upregulated under NaCl, PEG, and pathogen treatment compared to the control. Subcellular localization showed that *CpARF22* was localized in the nucleus. The transgenic *Arabidopsis thaliana* lines with the *CpARF22* gene enhanced their tolerance to salt and drought stress.

**Conclusion:**

In this study, we systematically analyzed the *CpARF* gene family and its expression patterns under drought, salt, and pathogen stress, which improved our understanding of the ARF protein of zucchini, and laid a solid foundation for functional analysis of the *CpARF* gene.

**Supplementary Information:**

The online version contains supplementary material available at 10.1186/s12864-024-09992-8.

## Background

Auxin-response factor (ARFs) is an essential family of transcription factors that bind to the Auxin-response elements (AuxRE) and play critical roles in a variety of plant development and physiological processes, as it has been shown to control the processes of cell differentiation, elongation, and division in conjunction with other plant-growth regulators [1]. ARF has a modular domain structure with three primary domains, and an N-terminal B3-like DNA-binding domain (DBD) is highly conserved, and DBD may bind to the ‘TGTCTC’ motif, a variable intermediate region that acts as an activating domain (AD) or inhibitory domain (RD) and a C-terminal dimer domain (CTD) contains motifs III and IV [2]. They are also present in the Aux/IAA family and are involved in protein–protein interactions through dimerization with auxin/indole-3-acetic acid (Aux/IAA) family genes and ARF genes [3]. Members of the Aux/IAA family are generally considered repressors of auxin-induced gene expression [4]. ARF genes are associated with many biological processes related to growth and development, such as embryogenesis, leaf expansion, leaf senescence, lateral root growth, and fruit development [5]. Auxin is also engaged in in plant responses to various environmental stresses, and it has been demonstrated that auxin can exert rapid and specific effects on genes at the molecular level [6].

At present, with the development of sequencing technology, a large number of ARF family members have been identified in numerous plants. There are 23 ARF genes in *Arabidopsis thaliana*, Under osmotic stress, the growth of *Arabidopsis thaliana* leaves was inhibited by enhancing the ARF-mediated auxin response [7]. Maize has 35 gene members and 23 of 35 ZmARF proteins containing domains III and IV, also observed at the C-terminus of Aux/IAA [8]. There are 15 *GbARF* members in *Ginkgo biloba*, of which *GbARF10b* and *GbARF10a* revealed transcriptional activators in auxin responses in male flower development [9]. 22 members of the gene family were identified in tomato, and eight *SlARF* genes were identified as repressors, five of which were activators [10]. There are 20 *StARF* genes in potato, most of which are highly responsive to abiotic stress-[11]. 31 Apple (*Malus pumila*) ARF genes have been identified in apple [12]. In addition, most *BvARF* genes were found to be strongly responsive to salt stress [13]. Research in Sorghum showed that the *SbARF* genes are mainly down-regulated under salt stress in the root, and most *SbARF* genes show significant down-regulation under drought stress, and the expression of three genes (*SbARF10,16* and *21*) was significantly up-regulated [14]. The similar study also showed that most *Arabidopsis thaliana* ARF genes were significantly down-regulated under salt and drought stress [15]. Recently, it has also been demonstrated that ARF genes are differentially expressed in resistance to wheat leaf rust infection, suggesting that they have some regulatory effects on plant-pathogen interactions [16]. Through the identification of the ARF gene of *Paulownia fortunei*, it was found that the *PfARF21* and *PfARF18/miR160h* modules played an important role in the response of *Paulownia fortunei* to phytoplasma infestation [17]. The principal functions of the ARF gene family are to control the transportation of materials in cells, and how to block the effective transportation of toxic substances secreted by the pathogen regulating this gene may reduce the occurrence probability of *Fusarium wilt*. This study also found a response relationship between *ARF3* gene expression in the ARF gene family and *Fusarium oxysporum* providing a good reference for further studies [18].

Zucchini has strong regeneration ability due to its thick root system, its stem can be up to 5 m long, and its branch is strong, its petiole is stout and its growth is strong, therefore, *Cucurbitaceae* crops generally have a stronger environmental adaptability than other plants [19]. In addition, zucchini is a new type of functional food with high nutritional value, there is a certain medicinal value and dietary health care role, the main components of the fruit to carbohydrates, contain a variety of soluble sugars, vitamin C, major elements, inorganic salts, protein, and other amino acids needed by the human body [20]. However, drought, salt and disease stress seriously affected the growth and productivity of zucchini. Therefore, breeding resistant and stress-resistant varieties is one of the main measures to solve the problem of disease control, and ARF family genes play a key role in abiotic and biological stress resistance, at present, the ARF family genes have not been reported in zucchini. Therefore, in this study, we identified the ARF genes of zucchini, and comprehensively analyzed the basic information of zucchini ARF proteins, gene structure, Cis-elements, gene duplication and so on. QRT-PCR to determine expression patterns under drought, salt stress, and Pathogen. These results provided valuable information for further studying the function of the ARF gene in zucchini.

### Materials and methods

#### Screening and Identification of *ARF* gene family in Zucchini

The whole genome sequences of zucchini were downloaded from the *Cucurbitaceae* database (http://cucurbitgenomics.org/organism/14). The *Arabidopsis thaliana* ARF protein sequences as target sequences to predict *CpARF* genes. The threshold value was E < e^−5^, and then the hypothetical ARF genes of zucchini was reached. The identified ARF genes were subjected to BLAST alignment on NCBI online software (http://blast.ncbi.nlm.nih.gov/Blast.cgi). Then, we used HMMER v3.3.2 (https://www.ebi.ac.uk/Tools/hmmer/) to search for conserved sequences and eliminate candidate genes for proteins that do not contain a specific ARF conserved domain (number: PF03638). The conserved *CpARF* domain was determined using tools NCBI-CDD (https://www.NCBI.nlm.nih.gov/CDD/), SMART (http://SMART.embl.de/), and Pfam (http://Pfam.xfam.org/). Finally, 33 *CpARF* genes were identified in zucchini.

### Phylogenetic Tree of the *CpARF* gene family and Analysis of Related Information

Expasy (https://web.Expasy.org/) online tool ProtParam was used to calculate physical and chemical properties of CpARF proteins, such as theoretical isoelectric point, instability index, relative molecular weight and hydrophobicity index. The subcellular location of the CpARF protein was analyzed by PSORT (http://psort1.hgc.jp/form.html) [21]. MEGA 11 software was used to perform multiple sequence alignment of ARF protein sequences of zucchini, pumpkin*,* and *Arabidopsis thaliana*, and then the phylogenetic tree was constructed by the maximum likelihood method. For example, MEGA 11 used 1000 repeated Bootstrap values with default values. Finally beautifying of the phylogenetic tree was through Evolview (http://120.202.110.254:8280/evolview) software [22].

### *CpARF* genes structure analysis, conservative motif prediction, and secondary structure analysis

The GFF annotation files of zucchini was imported into GSDS 2.0 (Gene Structure Display Server 2.0) (http://gsds.cbi.pku.e-du.cn/) to analyze the gene structure characteristics of the *CpARF* genes, and draw the exon–intron structure diagram was edited by the TBtools. MEME v5.0.5 software (http://meme-suite.org/tools/meme) was used to analyze the conserved motifs of the CpARF protein [23], and the number of motifs search count is set to 10. The secondary structure of CpARF protein was analyzed by NPS @ -GOR online server (https://npsa-prabi.ibcp.fr/cgi-bin/npsa_automat.pl?page=npsa_gor4.html).

## Chromosomal location of *CpARF* genes and gene duplication

TBtools was used to map *CpARF* genes on chromosome mapping by GFF annotation file. Moreover, we detected duplicate gene pairs using the plant genome replication database server (http://chibba.agtec.uga.edu/duplication/index/locket). ClustalW software was used to predict the amino acid sequences of the partially repeated CpARF proteins. The values of Ka and Ks were calculated by Tbtools.

### *CpARF* Cis-regulatory element analysis and protein interaction network construction

According to the GFF annotation file of zucchini, the Tbtools were used to extract the 2000 bp of DNA sequence upstream of the transcription start site of the *CpARF* family genes. PlantCARE was used to analyze the cis-acting elements of the *CpARF* genes promoter region [24]. STRING (https://cn.string-db.org/) software was used to construct the protein interaction network involved in the ARF gene using the *Arabidopsis thaliana* ARF gene as a template, and the protein interaction network of ARF proteins in zucchini was predicted by using picture editing software.

### Functional annotation analysis of *CpARF* genes

Based on amino acid sequences of CpARF proteins, BLAST2GO (https://www.blast2go.com/) was then used to obtain GO annotations. To investigate the metabolic pathways of CpARF proteins, the CpARF proteins were aligned to the KEGG database (https://www.kegg.jp).

### Plant materials, biotic, and abiotic stress treatments

The seeds used for this investigation originated from the college of plant protection, Gansu Agricultural University China. The seeds of zucchini were disinfected in 2% NaClO solution for 7 min to sterilize, washed three times with sterile distilled water, and the seeds were placed on filter paper and covered with petri dishes. The seeds were cultured for 3 days under dark conditions at 25 °C, and sterile water was sprayed every day to keep the filter paper moist. Ten seeds were placed in each petri dish for germination, and six petri dishes were shared. When the embryo grew to 2 cm, it was moved into the pot. Salt and drought stress treatments of zucchini: temperature 25℃, relative humidity 70%, light intensity 130 μmol·m^−2^·s^−1^, light–dark ratio 16 h / 8 h. When the third cotyledon was grown, the zucchini plants were treated with 200 mmol/L NaCl and 20% PEG, respectively. The treatment time was 0, 3, 6, 9, 12, 24 h. The leaves were quickly sampled in liquid nitrogen and stored at -80℃ for 3 portions each, then follow-up quantitative experiments were carried out. Pathogenic stress treatment of zucchini: The preserved strains of *F.oxysporum* were transferred to a PDA plate medium, and cultured in the dark at 25℃ for 7 d until the mycelium was covered with petri dishes. The spore suspension with a concentration of 1 × 10^7^ / ml was prepared with sterile water, and the seedlings with the same growth were selected. The prepared spore suspension was immersed in the root for 30 m, and the control group was immersed in the same amount of sterile water for 30 m. The leaves and roots of zucchini at 0 h, 12 h, 24 h and 48 h after pathogen infection were taken in turn and put into liquid nitrogen. Each treatment had three replicates and was stored at -80℃ for subsequent experiments.

### Expression Pattern Analysis and Real-time Quantitative PCR Analysis

Total RNA of leaves and roots was extracted from frozen samples using RNA simple Total RNA kit (Wuhan, China). Reverse transcription of RNA into cDNA used the Prime Script RT reagent kit (Wuhan, China). The SYBR Green Pro Taq HS premix qPCR kit (containing Rox) was used for qRT-PCR analysis. To detect the specificity of the primers, the target gene and the reference gene (β-Actin) were compared and verified in Cu Gen DB. The reaction system for qRT-PCR analysis on ABI7500 Real-Time PCR System (Applied Biosystems) was 20 μL, including 10 μL 2 × SYBR Green Pro Taq HS Premix (ROX plus)*^1^, 7 μL RNase free water, 1.0 μL primers, and 1.0 μL cDNA template. PCR reaction conditions: 95 °C 30 s, 95 °C 5 s, 60 °C 30 s, 40 cycles. 95 °C 15 s, 60 °C 1 min, 95 °C 15 s,. The results were calculated by the 2^−ΔΔCt^ method [25].

### Subcellular localization of the *CpARF22* protein

The cloned *CpARF22* gene was transferred to the expression vector containing the green fluorescent protein (GFP) by homologous recombination method, and the constructed GV1300: *CpARF22*-GFP vector plasmid was transformed into Agrobacterium GV1300. Agrobacterium strains containing plasmids were infiltrated into tobacco leaves for 48 h. The GFP fluorescence signal of CpARF22 protein was observed by confocal laser scanning microscopy (LSCM).

### Generation of transgenic *Arabidopsis thaliana* plants and determination of related physiological parameters

The CDS region of the *CpARF22* gene was submitted to the website of homologous recombination primer design In-Fusion Cloning: general information (http://www.takarabio.com) specific primers were designed. Forward Primer *CpARF22*-F: CGGGGGACGAGCTCGGTACCATGGCTTGCAATGGAGGAGATTC; Reverse Primer *CpARF22*-R: TGCTCACCATGTCGACTCAAACTGCAGGGGAGTCGG. The coding sequence of *CpARF22* gene was inserted into pCAMBIA-1302-EGFP vector. The obtained recombinant plasmid pCAMBIA-1302-*CpARF22* was introduced into E.coli DH5α, cultured in LB ( containing Kan) solid medium overnight at 37 °C, and a single colony was selected and cultured in 10 mL LB liquid medium containing Kan for 6–8 h. The suspended bacterial solution was used as a PCR bacterial solution identification template. The candidate-positive clones were sequenced, and the suitable monoclonal bacteria were sequenced. The plasmid was extracted and verified by enzyme digestion. The recombinant plasmid pCAMBIA-1302-*CpARF22* was transformed into agrobacterium-competent GV3101 and used to infect *Arabidopsis thaliana*. The transgenic seeds of T_0_ generation were sterilized and sown on an MS medium containing 50ug/mL hygromycin B. The T_1_ generation plants were obtained and DNA was extracted. Using the genomic DNA of non-transcription gene plants as negative control and plasmid pCAMBIA-1302-*CpARF22* as the positive control, the positive genetically modified crops were further identified by PCR, and then the T_2_ generation was screened by the same PCR method until the homozygous T_3_ generation transgenic *Arabidopsis thaliana* plants were obtained. The wild-type *Arabidopsis thaliana* seeds and the T_3_-generation homozygous seeds overexpressing *CpARF22* were sterilized and sown on 1/2MS medium containing 0,50 mM NaCl and 20% PEG for about 7 days, observe and record root length.

### Statistical analysis

All the statistical analyses were performed using Microsoft Excel 2010 and SPSS software (IBM, Armonk, NY). The means among various groups were compared by Duncan’s multiple range tests. The data were analyzed and are expressed as the means standard deviations (SDs), and *P* < 0.05 indicated significance difference.

## Results

### Genome-wide Identification of *ARF* gene family in Zucchini

By analyzing the whole genome sequence of zucchini, 33 candidate ARF members (Table 1, Table S1) were identified, named as *CpARF01*-*CpARF33*. The number of amino acids of the family members varied greatly, ranging from 438 aa (CpARF19) to 1071aa (CpARF20), and the average number of amino acids was 749. The molecular weight of the protein was 47,759.36 Da (CpARF19) to 117,813.27 Da (CpARF20). The PI values of the encoded proteins ranging from 4.99 to 8.54, and the PI values of 27 proteins were less than 7 (except CpARF04, CpARF06-CpARF09*,* and CpARF2*6*), which belonged to acidic proteins, suggesting that they may play a role in the acidic subcellular environment. The average coefficient of hydrophilicity ranged from -0.669 (CpARF05) to -0.350 (CpARF26). The instability index ranged from 40.74 (CpARF11) to 68.94 (CpARF03), both of which belonged to unstable protein, while the liposoluble index ranged from 62.56 (CpARF06) to 76.18 (CpARF32). The physical and chemical properties analysis showed that 33 CpARF proteins were unstable hydrophobic proteins, and the hydrophobicity index of all the proteins was less than 0, so 33 proteins were hydrophilic proteins. Prediction of subcellular localization showed that 13 proteins were located in the nucleus, 14 in the cytoplasm, 3 in the chloroplast matrix, 1 in the microbody, 1 in the cell membrane, and 1 in the periphery. 82% of CpARF proteins have the characteristics of transcription factors and play a role in the nucleus and cytoplasm.

**Table 1:** Basic physicochemical properties of the proteins encoded by the 33 *CpARF* family genes identified in the Zucchini Genome

Gene accession No	Gene	Size (aa)	Molecular weight	Isoelectric point	Instability index	Aliphatic index	GRAVY	Subcellular Localization	phosphorylation sites
Cp4.1LG01g00450.1	*CpARF01*	699	78,183.79	6.94	56.17	68.71	-0.595	nucleus	151
Cp4.1LG01g17160.1	*CpARF02*	550	61,055.49	5.32	48.26	73.73	-0.475	endoplasmic reticulum	106
Cp4.1LG01g19620.1	*CpARF03*	839	93,309.36	5.68	68.94	75.39	-0.466	cytoplasm	150
Cp4.1LG01g24390.1	*CpARF04*	688	76,157.80	7.29	47.21	72.56	-0.433	nucleus	126
Cp4.1LG01g22980.1	*CpARF05*	769	83,523.68	5.47	61.35	66.33	-0.669	chloroplast stroma	147
Cp4.1LG02g08050.1	*CpARF06*	589	63,259.83	7.73	54.61	62.56	-0.642	cytoplasm	161
Cp4.1LG05g07840.1	*CpARF07*	584	63,467.64	8.42	64.78	66.13	-0.532	cytoplasm	143
Cp4.1LG05g08160.1	*CpARF08*	838	91,462.81	8.03	55.03	74.14	-0.411	nucleus	150
Cp4.1LG05g08310.1	*CpARF09*	591	63,958.99	7.48	50.65	67.50	-0.568	cytoplasm	150
Cp4.1LG06g00800.1	*CpARF10*	880	96,839.30	5.09	54.17	73.55	-0.413	cytoplasm	142
Cp4.1LG07g01180.1	*CpARF11*	835	92,787.48	6.51	40.74	72.40	-0.491	outside	138
Cp4.1LG07g07700.1	*CpARF12*	953	105,600.58	6.20	62.16	70.55	-0.547	nucleus	124
Cp4.1LG07g10790.1	*CpARF13*	880	97,981.09	6.20	62.35	69.26	-0.584	cytoplasm	123
Cp4.1LG08g13310.1	*CpARF14*	607	67,287.59	5.29	60.25	70.64	-0.473	chloroplast stroma	124
Cp4.1LG09g07430.1	*CpARF15*	890	98,822.89	5.85	66.11	69.01	-0.511	cytoplasm	151
Cp4.1LG10g06190.1	*CpARF16*	891	98,898.43	6.18	63.98	75.30	-0.411	cytoplasm	165
Cp4.1LG10g05170.1	*CpARF17*	711	77,877.45	6.65	59.60	67.61	-0.435	nucleus	153
Cp4.1LG10g04760.1	*CpARF18*	587	64,771.64	7.00	48.47	69.47	-0.357	nucleus	105
Cp4.1LG11g04200.1	*CpARF19*	438	47,759.36	4.99	41.30	73.42	-0.372	microbody (peroxisome)	64
Cp4.1LG11g06740.1	*CpARF20*	1071	117,813.27	5.98	62.42	73.39	-0.494	nucleus	179
Cp4.1LG11g12020.1	*CpARF21*	847	93,282.59	5.62	57.99	70.06	-0.458	cytoplasm	111
Cp4.1LG13g00970.1	*CpARF22*	650	72,058.42	5.95	58.87	70.03	-0.570	chloroplast stroma	156
Cp4.1LG13g05520.1	*CpARF23*	836	93,036.11	5.56	66.38	72.39	-0.509	cytoplasm	132
Cp4.1LG13g09970.1	*CpARF24*	684	75,624.89	5.88	43.58	69.85	-0.439	nucleus	129
Cp4.1LG14g02660.1	*CpARF25*	727	81,703.23	6.74	51.59	69.42	-0.567	nucleus	169
Cp4.1LG16g05330.1	*CpARF26*	680	74,740.74	8.54	48.72	74.56	-0.350	nucleus	142
Cp4.1LG16g05510.1	*CpARF27*	719	79,772.38	5.84	54.87	67.09	-0.528	cytoplasm	153
Cp4.1LG17g10130.1	*CpARF28*	680	76,155.03	5.94	54.78	72.93	: -0.516	cytoplasm	144
Cp4.1LG18g09430.1	*CpARF29*	850	94,359.18	6.05	50.70	69.96	-0.516	nucleus	138
Cp4.1LG19g04050.1	*CpARF30*	705	78,158.47	6.83	48.27	69.84	-0.424	nucleus	128
Cp4.1LG19g06940.1	*CpARF31*	723	78,850.37	7.28	61.64	65.27	-0.413	nucleus	134
Cp4.1LG19g05880.1	*CpARF32*	840	93,309.15	5.86	65.38	76.18	-0.422	cytoplasm	171
Cp4.1LG20g05550.1	*CpARF33*	883	97,693.10	5.92	65.77	73.34	-0.396	cytoplasm	148

### Phylogenetic analysis of the *CpARF* family in Zucchini

**Fig. 1:**
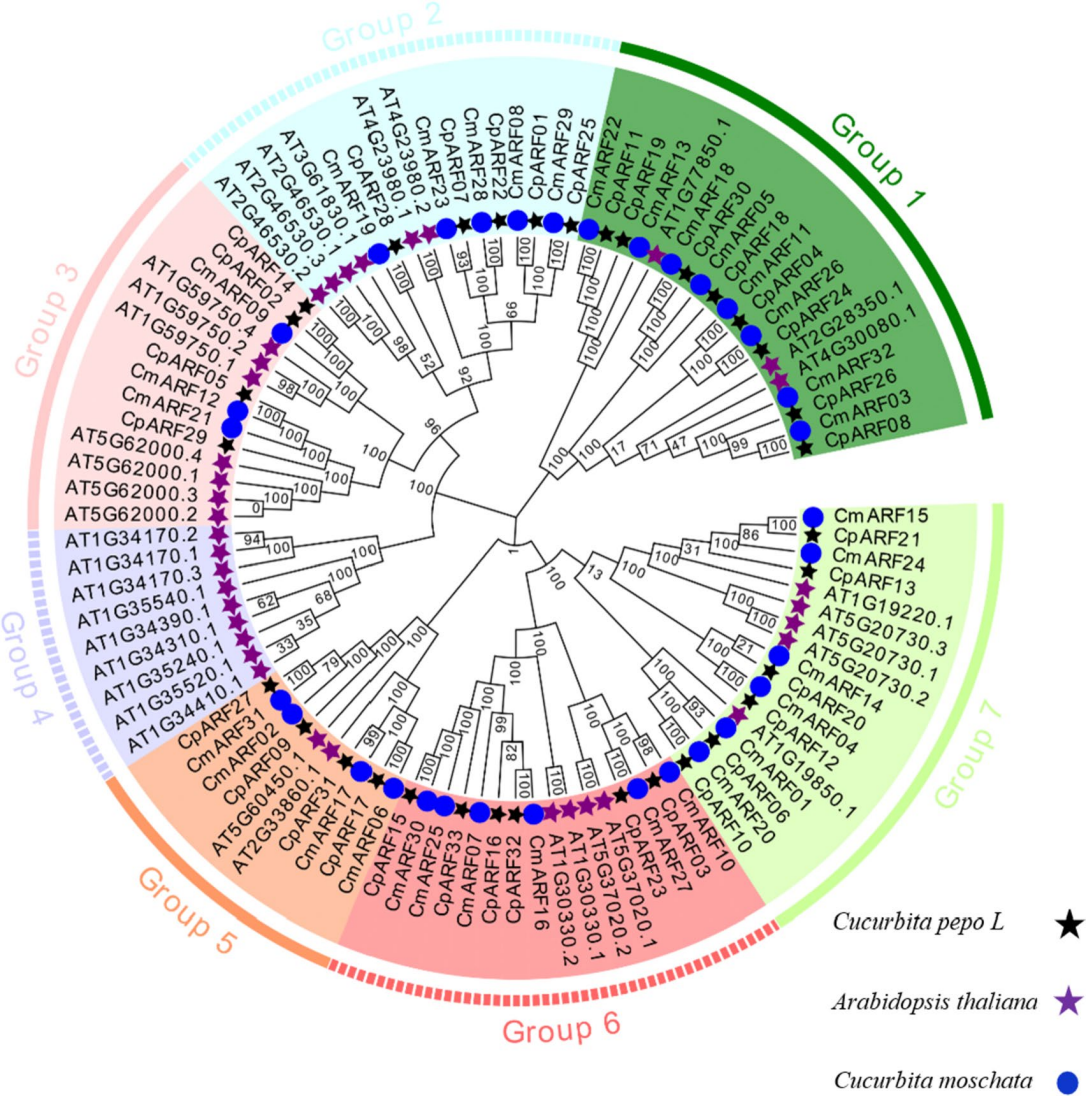
Phylogenetic analysis of *CpARF* protein families in *Arabidopsis thaliana*, pumpkin and zucchini. Pumpkin, *Arabidopsis thaliana and* zucchini are represented by the black pentagram, purple pentagram, and circle, respectively. The same species is represented by the same symbol with the same color. The numbers outside the branch represent the guided values based on 1000 repetitions

In order to study the genetic relationship between the ARF family members of zucchini and the ARF family such as *Arabidopsis thaliana* and pumpkin, the phylogenetic trees of 101 protein sequences of three species were constructed (Fig. 1, Table S2). The results showed that 101 members were divided into seven evolutionary branches, and the *CpARFs* genes of zucchini were distributed in six branches, indicating that the *CpARFs* family proteins were diverse. It was found that one pair of paralogous *CpARF* gene pairs (*CpARF14* and *CpARF02*); 23 pairs of orthologous gene pairs were found between pumpkin and zucchini(*CpARF08/ CmARF03, CpARF24/ CmARF26, CpARF04/ CmARF11, CpARF30/ CmARF18, CpARF11/ CmARF22, CpARF25/ CmARF29, CpARF01/ CmARF08, CpARF22/ CmARF28, CpARF07/ CmARF23, CpARF28/ CmARF19, CpARF05/ CmARF12, CpARF27/ CmARF31, CpARF31/ CmARF17, CpARF17/ CmARF06, CpARF15/ CmARF30, CpARF32/ CmARF16, CpARF23/ CmARF27, CpARF03/ CmARF10, CpARF10/ CmARF20, CpARF06/ CmARF01, CpARF12/ CmARF04, CpARF20/ CmARF14 and CpARF21/ CmARF15*), it suggests that there is a close relationship between pumpkin and zucchini.

### Gene structure and conserved motif analysis

**Fig. 2:**
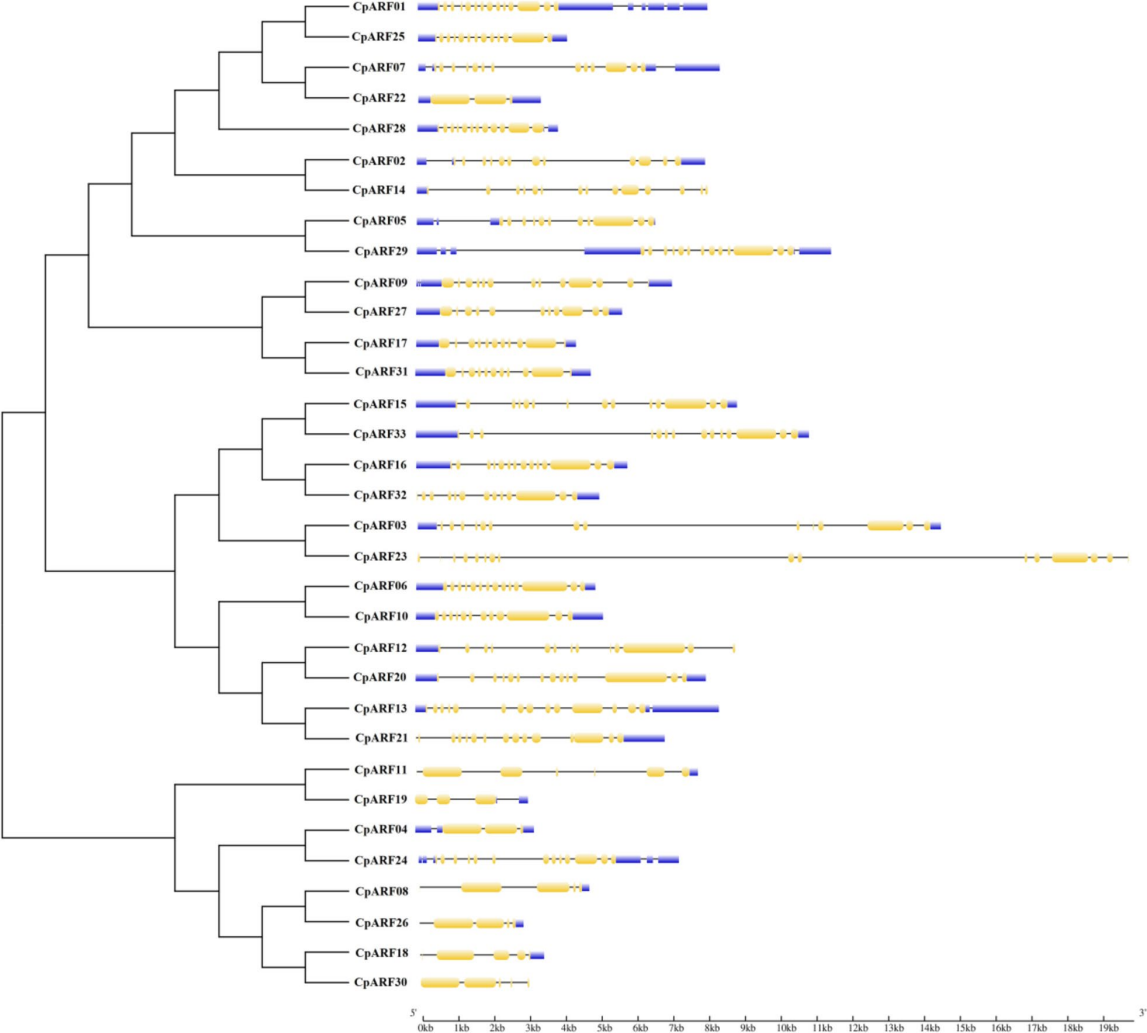
Phylogenetic relationship and exon–intron structure analysis of ARF family in zucchini. Exon–intron structure analysis was performed using the online tool GSDS. The length of exons and introns of each *CpARF* gene shows that the value includes CDS, UTR, and introns. The yellow value is the CDS coding region, and the blue value is the UTR coding region

Gene structure analysis showed that 33 *CpARF* genes contained exons and introns, and the number of exons ranged from 3 to 16. Among them, the number of exons of 25 *CpARF* genes were greater than 10. The number of exons of *CpARF05*, *CpARF08*, *CpARF19*, *CpARF24,* and *CpARF26* genes ranged from 2 to 4, and the number of exons of the remaining 3 genes ranged from 5 to 6. In addition, we found that most genes have terminal non-coding sequences, of which 22 genes have 5 ' and 3 ' terminal non-coding sequences, *CpARF08*, *CpARF11*, *CpARF16*, *CpARF19*, *CpARF21*, *CpARF26*, *CpARF31* have 3 ' terminal non-coding sequences, *CpARF12*, *CpARF14* have 5 ' terminal non-coding sequences, *CpARF23*, *CpARF30* do not have terminal non-coding sequences, 94% of the genes have terminal non-coding sequences. At the same time, it was found that the genes in different subfamilies have distinct gene structures, such as *CpARF11* and *CpARF16*, the length difference was large, suggesting that the genes had mutated during evolution (Fig. 2). These results indicated that the structural differences of *CpARFs* genes may affect their functions. Therefore, it is necessary in order to further analyze the functions of these genes.

**Fig. 3:**
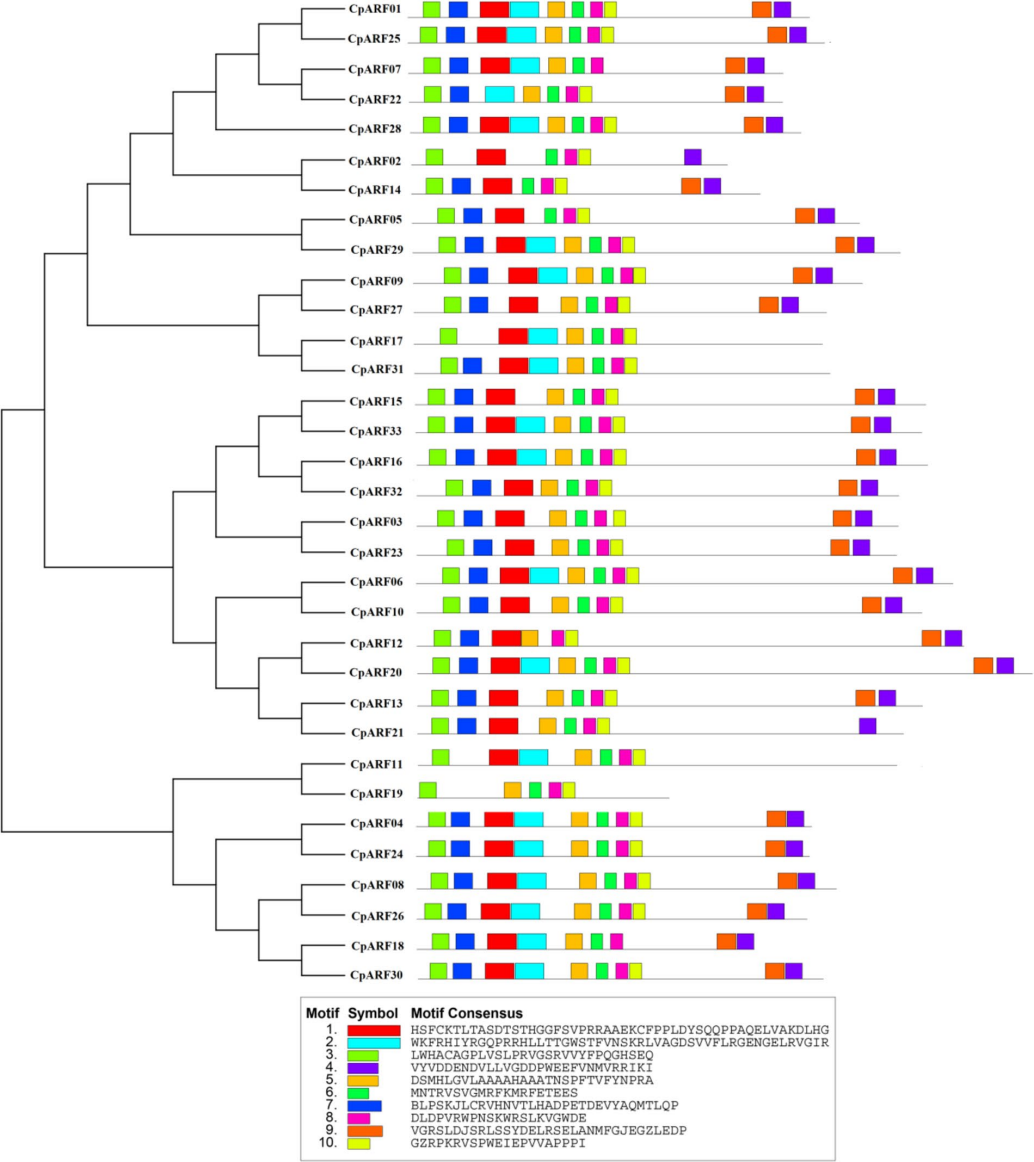
Phylogenetic relationship and conserved motif analysis of ARF family in zucchini analysis of Chromosome Location and Gene Duplication

Through Motif analysis of 33 CpARF proteins (Fig. 3), it was found that Motif 1 exists in all CpARFs( expect CpARF19, CpARF22), Motif 2 exists in 20 CpARFs (CpARF02, CpARF03, CpARF05, CpARF10, CpARF12-CpARF15, CpARF19, CpARF21, CpARF23, CpARF27, CpARF32), and Motif 3 exists in all CpARFs. Motif 4 exists in all CpARFs (expect CpARF11, CpARF17, CpARF19, CpARF31), Motif 5 exists in all CpARFs (except CpARF02, CpARF05, CpARF14), Motif 6 exists in all CpARFs (except CpARF12), Motif 7 exists in all CpARFs (CpARF02, CpARF11, CpARF17, CpARF19), Motif 8 exists in all CpARFs. Motif 9 exists in all CpARFs (except CpARF02, CpARF11, CpARF19, and CpARF21), and motif 10 exists in all CpARFs (except CpARF03 and CpARF18). At the same time, we can note that the proteins in the same subfamily have similar basic motifs. The 33 CpARF proteins have conserved motifs between 5–10, of which 14 CpARF proteins have 10 conserved motifs, 10 CpARF proteins have 9 conserved motifs, 5 CpARF proteins have 8 conserved motifs (CpARF05, CpARF12, CpARF14, CpARF21, CpARF31), and 2 CpARF proteins have 7 conserved motifs (CpARF11, CpARF17). CpARF02 protein has 6 conserved motifs, and CpARF19 protein has 5 conserved motifs. It can be observed that motifs 1,3,6,8 and 10 are particularly conserved in most CpARF proteins. This difference in conserved motifs may also serve as one of the reasons for the functional differences in *CpARF* genes.

**Fig. 4:**
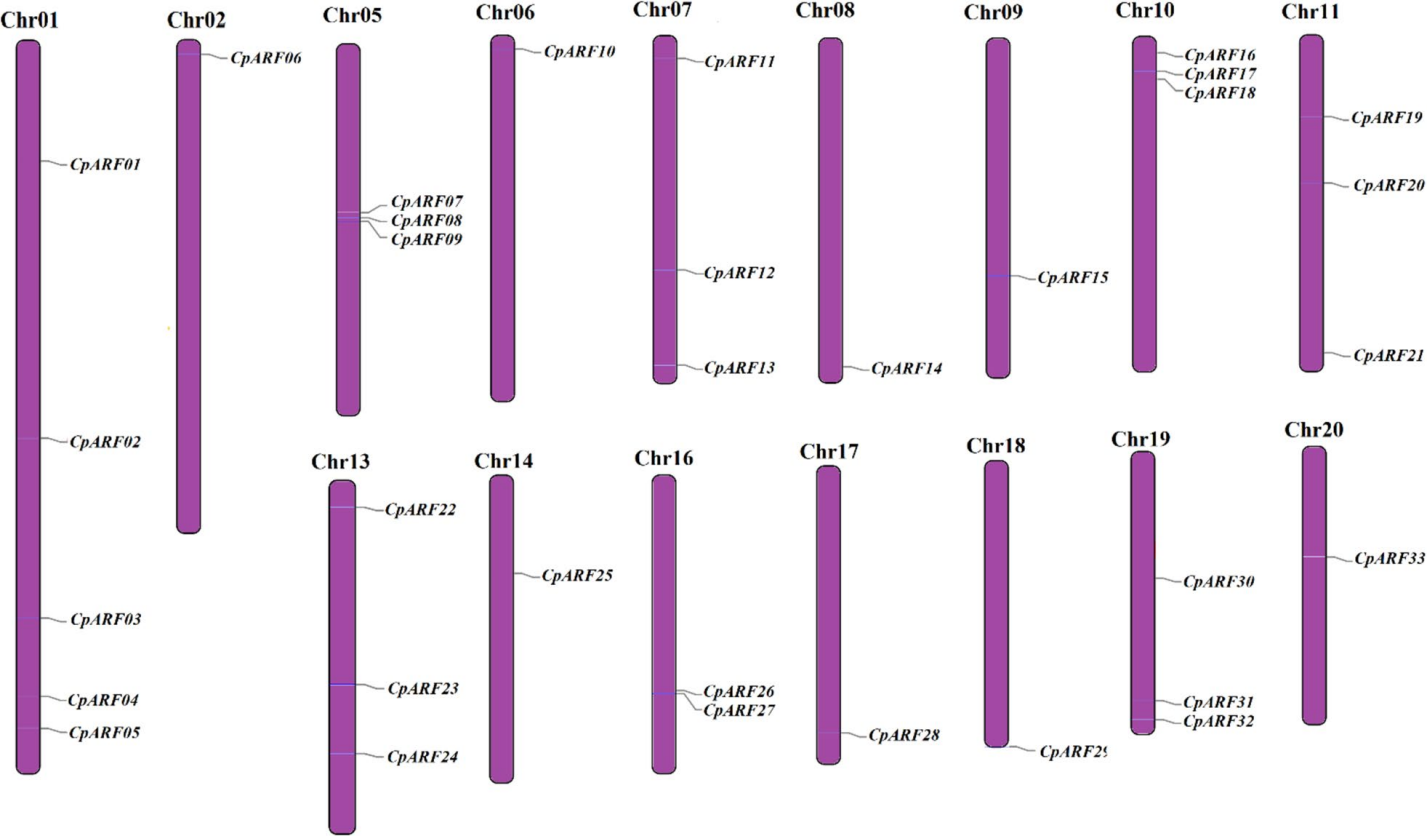
The chromosome location map of the *ARF* gene in zucchini was drawn by the MapDraw program, and the name of the gene was marked on the right side of the chromosome

The results showed that 33 *CpARF* genes were unevenly distributed on all 16 chromosomes of zucchini. There were 5 *CpARF* genes on chromosome 1, 3 *CpARF* genes on chromosomes 5,7,10,11,13, and 19 respectively, 2 *CpARF* genes on chromosome 16, and 1 *CpARF* gene on chromosomes 2,6,8,9,14,17,18, and 20 respectively (Fig. 4).

The evolutionary selection of zucchini *CpARF* genes was tested by calculating non-synonymous (Ka) and synonymous (Ks) substitutions (Table 2). A total of 20 pairs of duplicated gene pairs were discovered in the *CpARF* genes, and all of them were fragment duplications. In general, the ratio of Ka/Ks > 1 indicates positive selection, Ka/Ks < 1 indicates underwent purifying selection pressure, and Ka/Ks = 1 indicates neutral selection. The results showed that the Ka/Ks ratios of 20 pairs of *CpARF* genes were < 1 (Table 3), indicating that these genes underwent purifying selection pressure during evolution. At the same time, we found that the time of duplication of these genes varied greatly, and the repeated years ranged from 6.785 (*CpARF07/CpARF22*) and 119.870 MYA (*CpARF26/CpARF30*).

**Table 2:** The Ka/Ks ratios and predicted duplication dates for duplicated *CpARF* Genes in Zucchini

Duplicated CpARF gene1	Duplicated CpARF gene2	Ka	Ks	Ka/Ks	Date (MYA) = Ks/2λ	Selective pressure	Duplicate type
*CpARF01*	*CpARF22*	0.265	1.362	0.195	45.387	Purifying selection	Segmental
*CpARF03*	*CpARF23*	0.041	0.322	0.127	10.718	Purifying selection	Segmental
*CpARF04*	*CpARF24*	0.112	0.560	0.200	18.665	Purifying selection	Segmental
*CpARF06*	*CpARF10*	0.058	0.355	0.162	11.830	Purifying selection	Segmental
*CpARF07*	*CpARF22*	0.068	0.204	0.332	6.785	Purifying selection	Segmental
*CpARF08*	*CpARF26*	0.039	0.444	0.087	14.811	Purifying selection	Segmental
*CpARF09*	*CpARF27*	0.058	0.266	0.217	8.850	Purifying selection	Segmental
*CpARF11*	*CpARF19*	0.219	0.318	0.689	10.601	Purifying selection	Segmental
*CpARF12*	*CpARF20*	0.074	0.533	0.139	17.767	Purifying selection	Segmental
*CpARF13*	*CpARF21*	0.080	0.379	0.210	12.624	Purifying selection	Segmental
*CpARF15*	*CpARF16*	0.149	1.737	0.086	57.898	Purifying selection	Segmental
*CpARF15*	*CpARF32*	0.143	1.455	0.098	48.516	Purifying selection	Segmental
*CpARF16*	*CpARF32*	0.053	0.345	0.153	11.491	Purifying selection	Segmental
*CpARF17*	*CpARF31*	0.071	0.378	0.189	12.612	Purifying selection	Segmental
*CpARF18*	*CpARF04*	0.287	2.188	0.131	72.933	Purifying selection	Segmental
*CpARF18*	*CpARF30*	0.053	0.371	0.143	12.366	Purifying selection	Segmental
*CpARF22*	*CpARF25*	0.262	1.593	0.165	53.100	Purifying selection	Segmental
*CpARF24*	*CpARF26*	0.307	2.349	0.131	78.312	Purifying selection	Segmental
*CpARF24*	*CpARF30*	0.327	1.832	0.179	61.051	Purifying selection	Segmental
*CpARF26*	*CpARF30*	0.263	3.596	0.073	119.870	Purifying selection	Segmental

**Table 3:** Secondary Structure of CpARF proteins

Gene	Alpha helix	Extended strand	Random coil
*CpARF01*	169 (24.18%)	145 (20.74%)	385 (55.08%)
*CpARF02*	126 (22.91%)	109 (19.82%)	315 (57.27%)
*CpARF03*	192 (22.88%)	165 (19.67%)	482 (57.45%)
*CpARF04*	133 (19.33%)	151 (21.95%)	404 (58.72%)
*CpARF05*	192 (24.62%)	130 (16.67%)	458 (58.72%)
*CpARF06*	175 (18.72%)	203 (21.71%)	557 (59.57%)
*CpARF07*	135 (20.71%)	149 (22.85%)	368 (56.44%)
*CpARF08*	147 (20.14%)	182 (24.93%)	401 (54.93%)
*CpARF09*	143 (18.26%)	171 (21.84%)	469 (59.90%)
*CpARF10*	177 (20.11%)	164 (18.64%)	539 (61.25%)
*CpARF11*	242 (28.98%)	152 (18.20%)	441 (52.81%)
*CpARF12*	254 (26.65%)	155 (16.26%)	544 (57.08%)
*CpARF13*	247 (28.07%)	133 (15.11%)	500 (56.82%)
*CpARF14*	99 (16.31%)	127 (20.92%)	381 (62.77%)
*CpARF15*	195 (21.91%)	164 (18.43%)	531 (59.66%)
*CpARF16*	219 (24.58%)	157 (17.62%)	515 (57.80%)
*CpARF17*	120 (16.88%)	140 (19.69%)	451 (63.43%)
*CpARF18*	95 (16.18%)	128 (21.81%)	364 (62.01%)
*CpARF19*	86 (19.63%)	86 (19.63%)	266 (60.73%)
*CpARF20*	278 (25.96%)	164 (15.31%)	629 (58.73%)
*CpARF21*	217 (25.62%)	147 (17.36%)	483 (57.02%)
*CpARF22*	131 (20.15%)	145 (22.31%)	374 (57.54%)
*CpARF23*	177 (21.17%)	158 (18.90%)	501 (59.93%)
*CpARF24*	132 (19.30%)	145 (21.20%)	407 (59.50%)
*CpARF25*	177 (24.35%	167 (22.97%)	383 (52.68%)
*CpARF26*	129 (18.97%)	177 (26.03%)	374 (55.00%)
*CpARF27*	137 (19.05%)	149 (20.72%)	433 (60.22%)
*CpARF28*	191 (28.09%)	124 (18.24%)	365 (53.68%)
*CpARF29*	177 (20.82%)	178 (20.94%)	495 (58.24%)
*CpARF30*	103 (14.61%)	159 (22.55%)	443 (62.84%)
*CpARF31*	92 (12.72%)	134 (18.53%)	497 (68.74%)
*CpARF32*	214 (25.48%)	148 (17.62%)	478 (56.90%)
*CpARF33*	191 (21.63%)	170 (19.25%)	522 (59.12%)

### Secondary structure analysis of CpARF proteins

The secondary structure of the ARF proteins of zucchini showed that the proteins encoded by *CpARF* genes of zucchini contained α-helix, extended chain, and random coil. The overall structural similarity was very different and the complexity was general. The distribution of random coils in ARF proteins is the most as shown in Table 3. The proportion of CpARF10*,* CpARF14*,* CpARF17-19*,* CpARF27*,* CpARF30*,* and CpARF31 in random coils is greater than 60%, and the remaining 25 are between 50 and 60%. The distribution of α-helix and extended strand in ARF proteins is second. In the extended chain, CpARF01*,* CpARF04*,* CpARF06-CpARF09*,* CpARF14*,* CpARF18*,* CpARF22*,* CpARF24-27, CpARF29*,* and CpARF30 accounted for more than 20%, and the remaining 18 CpARF proteins were between 15 and 20%. In the α-helix, CpARF01*-*CpARF03*,* CpARF05*,* CpARF07*,* CpARF08*,* CpARF10-13*,* CpARF15*,* CpARF16*,* CpARF2*0*-23*,* CpARF25*,* CpARF28*,* CpARF29*,* CpARF32, and CpARF33 accounted for more than 20%, and the remaining 12 CpARF proteins are between 12% -20%. The prediction shows that most of them are composed of random coils, which is in line with the secondary structure prediction results.

### Protein interaction and promoter element analysis

**Fig. 5:**
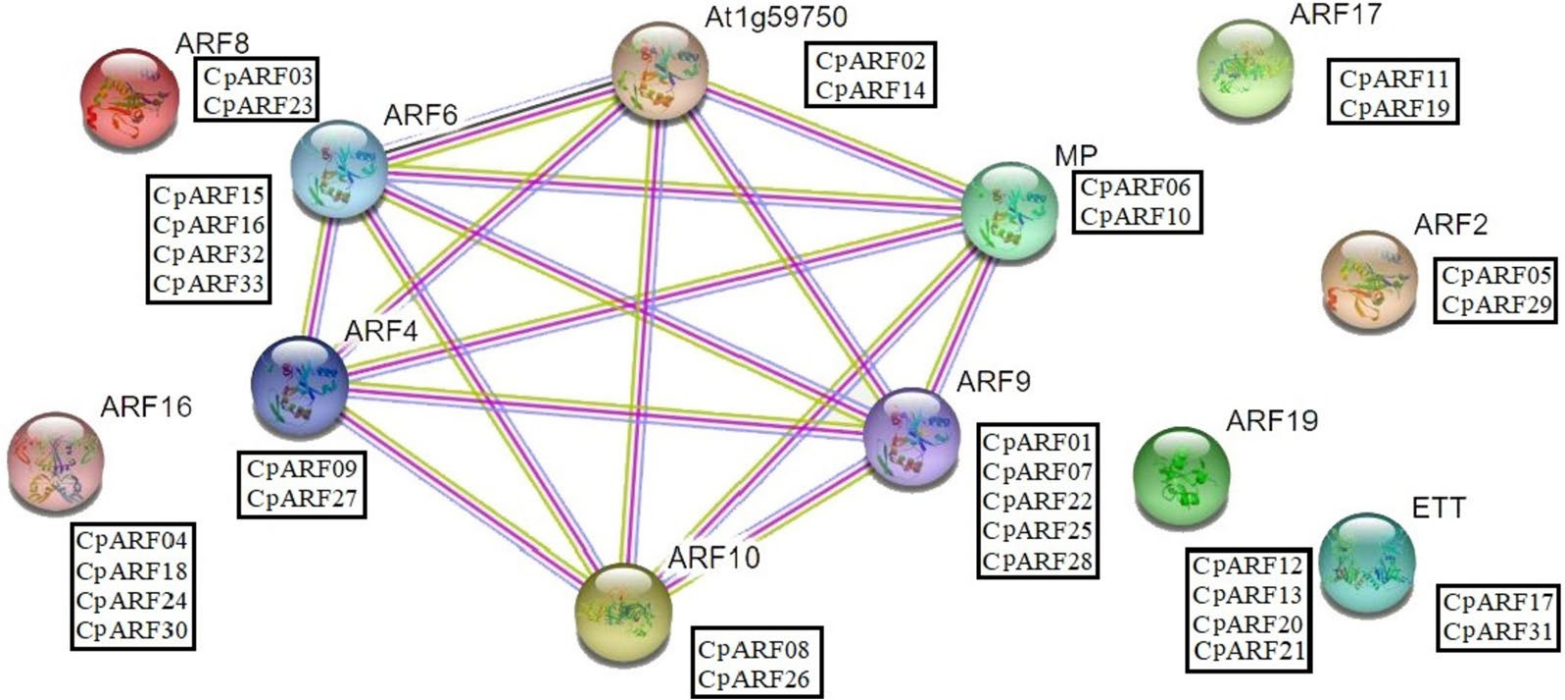
The prediction of the interaction network of *CpARF* proteins based on the interactions of their orthologs in *Arabidopsis thaliana*

In order to predict the interaction between CpARF proteins, String software was utilized to construct the ARF protein interaction network of zucchini based on the homologous sequence of *Arabidopsis thaliana* (Fig. 5). Depending to the prediction results, 12 CpARF proteins appeared in the known interaction network of *Arabidopsis thaliana* ARF proteins, indicating that there is a complex relationship between the two. We observed that the protein sequence of ARF9 was closely related to five CpARF protein sequences (CpARF01, CpARF07, CpARF22, CpARF25, CpARF28), ARF8 protein and two CpARF protein (CpARF03, CpARF23) are homologous, ARF16 protein and four CpARF protein sequences (CpARF04, CpARF18, CpARF24, CpARF30) are homologous, ARF2 protein and two CpARF protein sequences (CpARF05, CpARF29) are homologous, ETT and two CpARF proteins (CpARF17, CpARF31) were highly similar, these results suggested that these homologue zucchini genes may have similar functions. At the same time, *Arabidopsis thaliana* ARF interacts with multiple proteins with known functions and unknown functions, indicating that it has a complex regulatory mechanism, so similar CpARF proteins may also have analogous functions. ARF6 and ARF8 regulate the maturation of male and female floral organs in *Arabidopsis thaliana*, and the removal of these proteins in ARF6 ARF8 double mutants or plants over-expressing miR167 can lead to infertility [26, 27]. ARF4 interacts with auxin / Indole-3-Acetic-Acid12 (IAA12), and the expression signal of ARF4 shows a dynamic pattern analogous to that of ARF5 and IAA12 during bud meristem formation. The induced expression of ARF4 complemented the regenerative phenotype of IAA12 over-expression but did not rescue the defects in the arf5 mutant mp-S319 [28]. Auxin response factor 9 (ARF9) and forkhead-associated (FHA) domains are directly involved in plant development through various pathways, such as hormone regulation, plant morphology, embryogenesis, and DNA repair. Reproducible TDFs are sequenced and characterized for homology analysis, annotation, protein–protein interaction, subcellular localization and physical mapping [29]. After MP is mutated, it can limit the movement of the virus in the plant to reduce the occurrence of viral diseases [30]. Auxin response factor ARF10, as a derepressed transcription factor in auxin signal transduction, is a positive regulator in ABA signal transduction, and ARF10 regulates the dormancy and germination of *Arabidopsis thaliana* seeds [31].

**Fig. 6:**
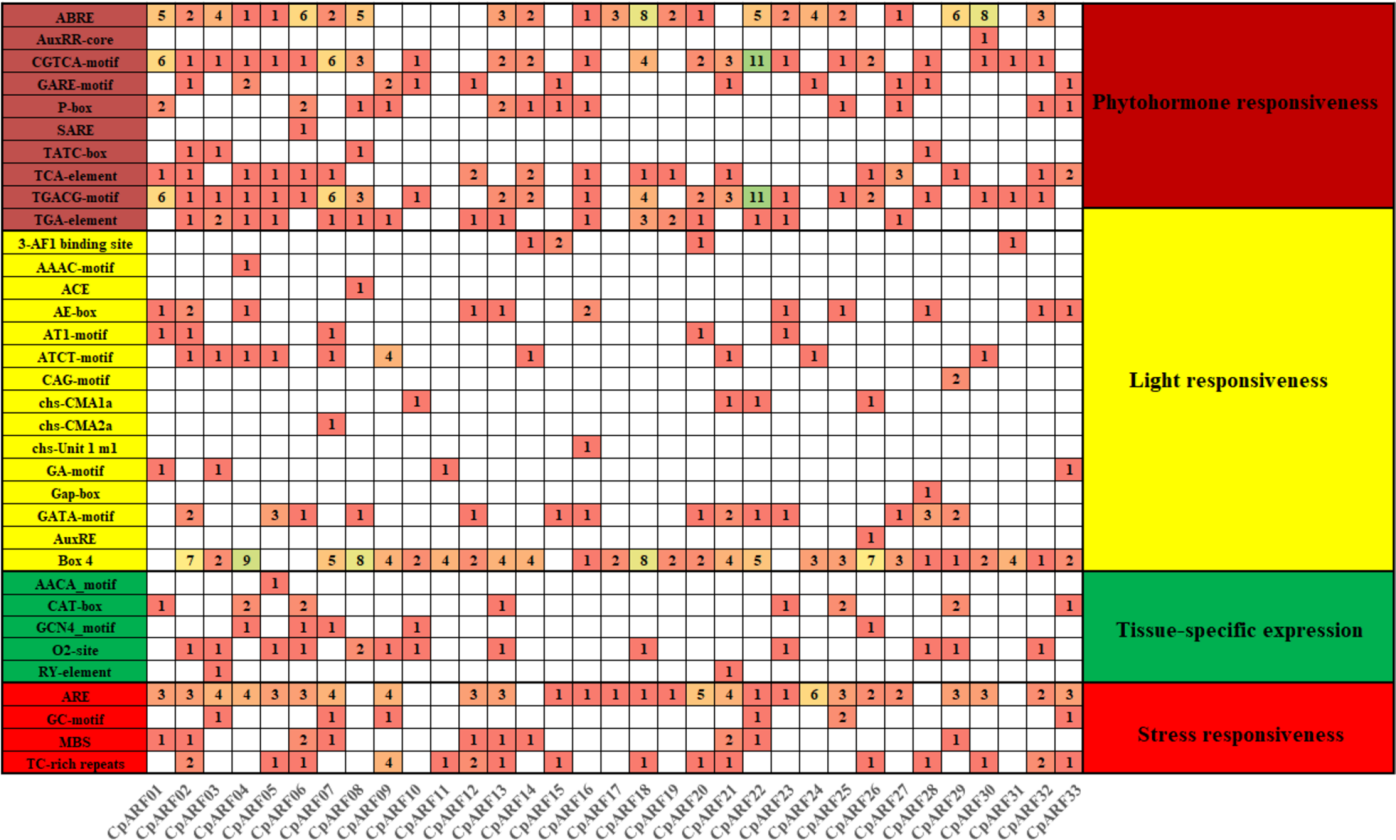
The cis-regulatory element in the promoter region of *CpARF* genes of zucchini. The colors and numbers on the grid indicate the number of different cis-regulatory elements in the *CpARF* genes

Cis-acting elements are transcription factor-specific binding sites and thus play an important role in regulating genes responsible for the growth, differentiation, and development of organisms, including plants (Fig. 6, Table S3). According to the functional annotation, we obtained 34 cis-acting elements and found that 29.4% (10) were related to hormone response. Among them, the abscisic acid response element (ABA) has ABRE, which exists in the promoter region of all *CpARF* genes (except C*pARF09-CpARF12, CpARF15, CpARF21, CpARF26, CpARF28, CpARF31, CpARF33*). The gibberellin response elements (GA) have GARE-motif, P-box, TATC-box, in which TATC-box only exists in *CpARF02, CpARF03*, *CpARF08, CpARF28*, jasmonic acid response elements (JA) have CGTA-motif, TGACG-motif, salicylic acid response elements (SA) have SARE, SARE only exists in *CpARF06*, auxin response elements (IAA) have TGA-element, AuxRR-core, in which AuxRR-core only exists in *CpARF30*. 44.1% (15) were linked to light, and the light response elements were (3-AF1 binding site, ACE, AE-box, AT1-motif, and other elements). Among them, 3-AF1 binding site existed in *CpARF14, CpARF15, CpARF20, and CpARF31*, AAAC-motif only existed in *CpARF04* 14.7% (4) of them were related to pressure, and the vital element for anaerobic induction was ARE. ARE existed in 27 *CpARFs* (except *CpARF08, CpARF10, CpARF11, CpARF14, CpARF28, and CpARF31*), and the similar enhancer element GC-motif involved in hypoxia-specific induction. It also was in 27 *CpARFs* (except *CpARF03, CpARF07, CpARF09, CpARF22, CpARF25, and CpARF33*), drought response element (MBS). Cis-acting elements TC-rich repeats are involved in defense and stress responses. 8.8% (5) were linked to tissue-specific expression-related elements. The endosperm-specific expression element had AACA-motif, which only existed in *CpARF05*. The meristem-specific element (CAT-box) existed in 8 *CpARF* genes (*CpARF01, CpARF04, CpARF06, CpARF13, CpARF23, CpARF25, CpARF29, CpARF33*). Seed-specific expression of PP2Cphosphatase element (RY-element) exists in *CpARF03, CpARF21*. The box is available in almost all *CpARFs* (except *CpARF01, CpARF04, CpARF13, CpARF23, CpARF25, CpARF29, CpARF33*). In addition, we found that *CpARF22* contains 38 cis-elements, followed by *CpARF07* and *CpARF18* which contain 31 cis-elements. The number of cis-elements in most *CpARF* genes is between 11 and 28, and the remaining *CpARFs* contain the least cis-elements. *CpARF10* contains 8 cis-elements, *CpARF15* contains 7 cis-elements, *CpARF17* contains 6 cis-elements, *CpARF19* contains 8 cis-elements, *CpARF31* contains 7 cis-elements, and *CpARF11* contains 3 cis-elements.

### Function enrichment analysis of KEGG and GO

**Fig. 7:**
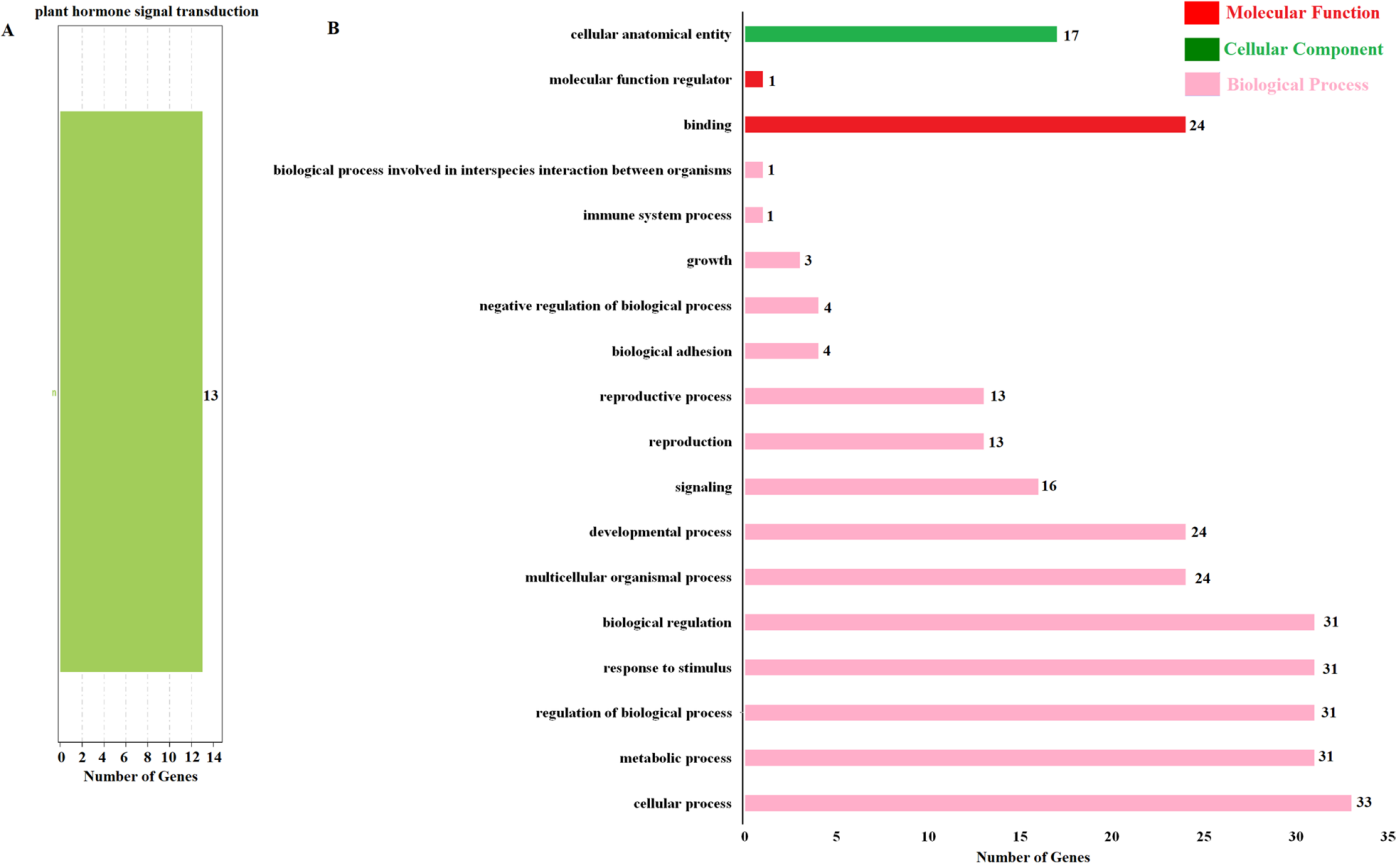
Function annotation Analysis of KEGG and GO. A: KEGG Function annotation; B: GO Function annotation

We analyzed the function of these genes based on KEGG database and found that 13 ARF genes (*CpARF01, CpARF02, CpARF06, CpARF07, CpARF10, CpARF13, CpARF14, CpARF17, CpARF21, CpARF22, CpARF25, CpARF28* and *CpARF31*) were involved in plant hormone signal transduction pathway. In addition, GO analysis showed that ARF family genes were involved in 18 terms, involving biological process (15 terms), molecular function (2 terms) and cellular component (1 term). We found that 33 genes were involved in cellular process, 31 genes (expect *CpARF19* and *CpARF22*) were involved in metabolic process, regulation of biological process, response to stimulus, biological regulation (Fig. 7).

### Expression analysis of ARF Gene in Zucchini in response to salt stress

**Fig. 8:**
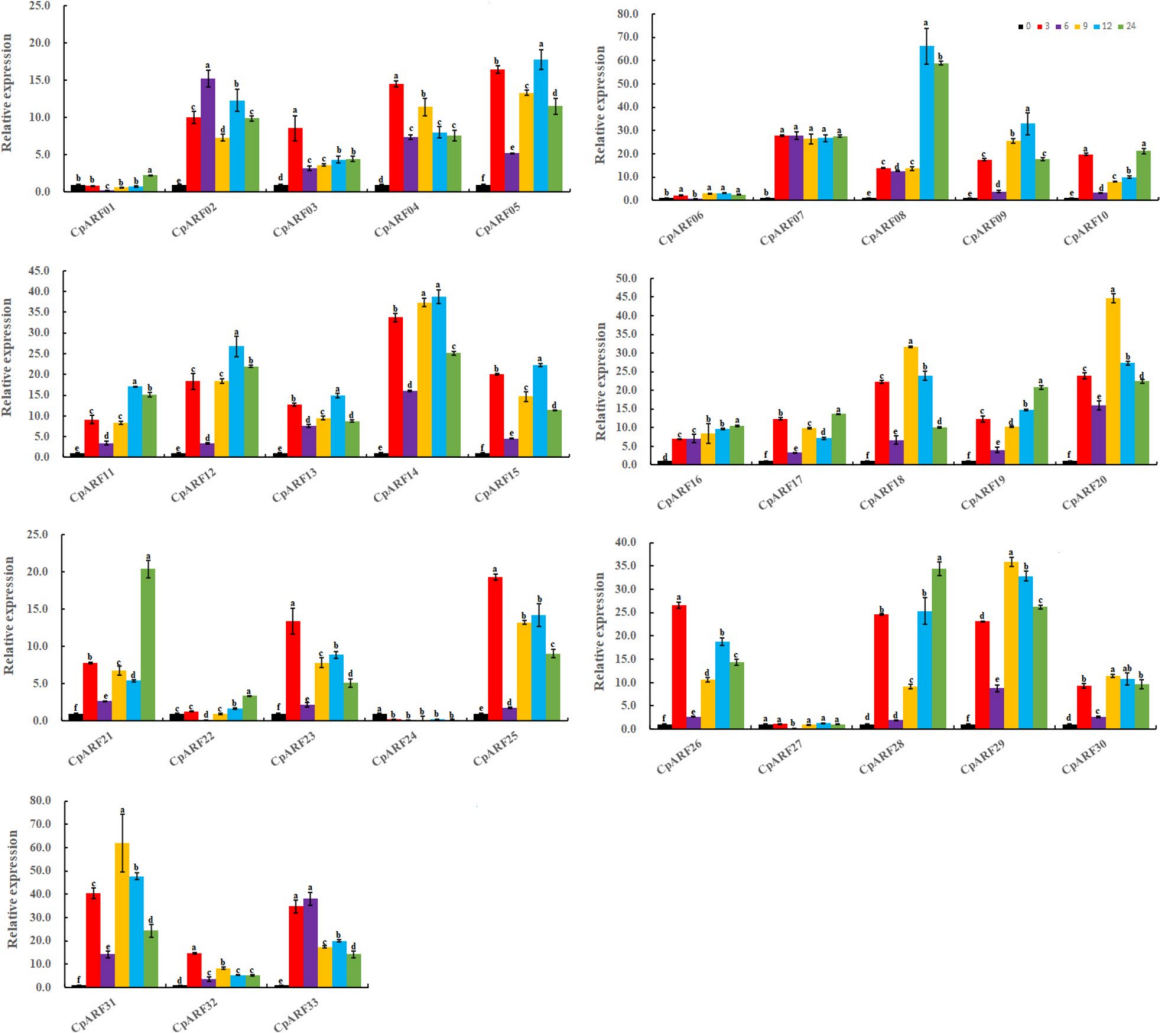
The expression profiles of auxin response factor (*ARF*) genes in zucchini in response to NaCl treatment. The relative expression levels of 33 ARF genes in leaves were measured at 0, 3, 6,9,12,24 h of treatment. The relative mRNA levels of the group at 0 h were used for reference. The relative expression was calculated using the method of 2^−△△Ct^. Values are means ± SD (*n* = 3). * represent significance at *p* < 0.05 compared with the references

Auxin response factors (ARFs) play a major role in the differential expression of auxin in response to various abiotic stresses. In addition, there were significant differences in the expression levels of zucchini genes under salt stress (Fig. 8, Table S4, Table S5). Here, we treated zucchini seedlings with 200 mmol / L NaCl for 0 h, 3 h,6 h, 9 h, 12 h, and 24 h, and then assessed the expression of 33 *CpARF* genes using qRT-PCR. All the *CpARF* genes (except *CpARF24* and *CpARF27*) showed a rapid response to salt stress. Among them, *CpARF04, CpARF23, CpARF25,* and *CpARF26* reached the most meaningful level of NaCl treatment after 3 h. After 6 h of salt treatment, only *CpARF02* had the most significant expression level. *CpARF18, CpARF20, CpARF29,* and *CpARF31* reached the maximum at 9 h, and some genes had the most significant expression at 12 h (*CpARF05, CpARF08, CpARF09, CpARF11, CpARF12, CpARF14*). The expression values of *CpARF10*, *CpARF21,* and *CpARF28* were the highest under 24 h salt treatment. Among them, the expression trends of six *CpARF* genes (*CpARF03, CpARF10, CpARF17, CpARF19, CpARF22, CpARF28*) were first up-regulated and then down-regulated and then up-regulated. The expression trends of two genes (*CpARF07, CpARF16*) were continuously up-regulated, and the expression of *CpARF21* was the most complex, it was up-regulated first, then down-regulated, then up-regulated, then down-regulated and finally up-regulated, the expression trends of the remaining 19 genes were first up-regulated, then down-regulated, then up-regulated and then down-regulated.

### Expression analysis of ARF Gene in Zucchini in response to drought stress

**Fig. 9:**
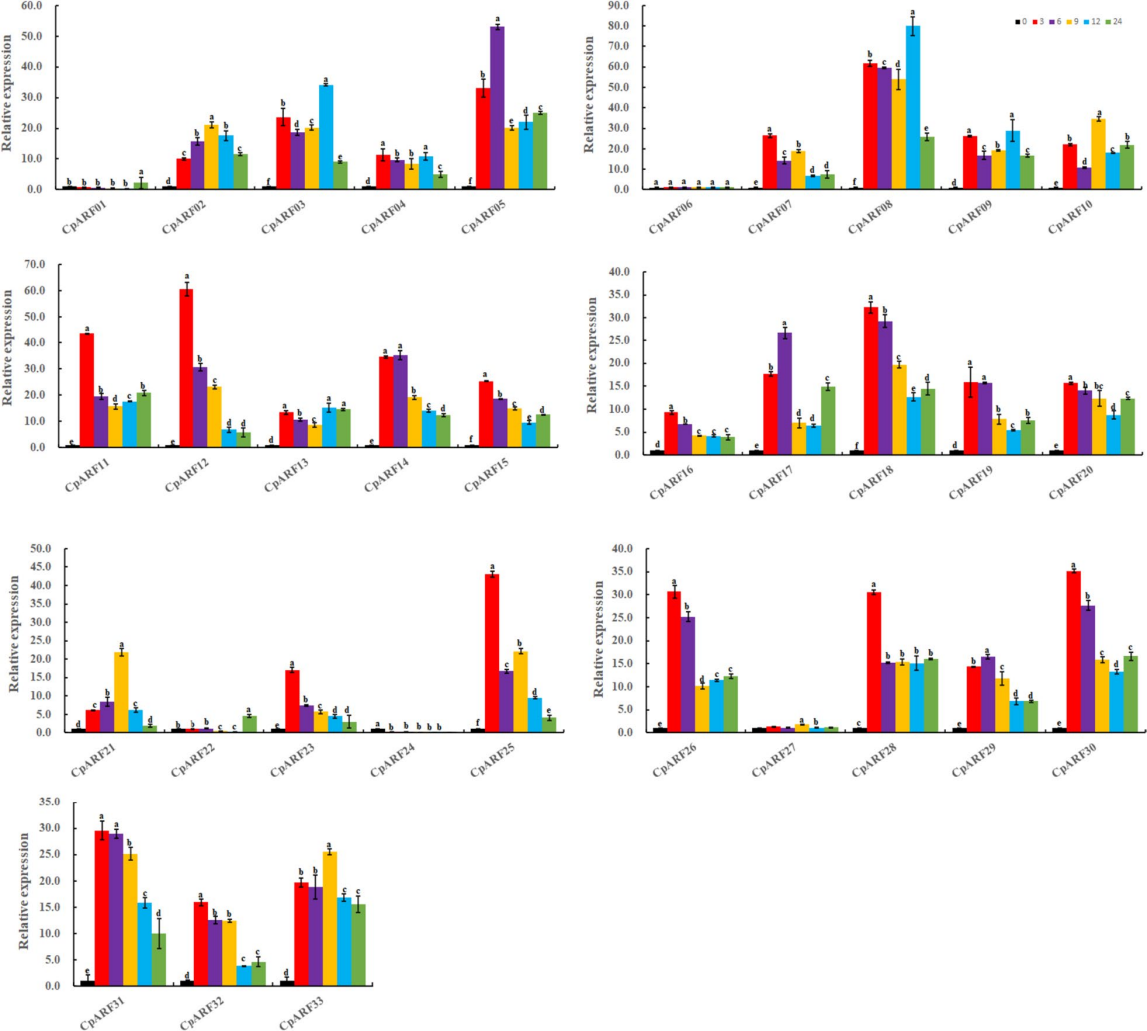
The expression profiles of auxin response factor (*ARF*) genes in zucchini in response to PEG treatment. The relative expression levels of 33 ARF genes in leaves were measured at 0, 3, 6,9,12,24 h of treatment. The relative expression levels of the group at 0 h were used for reference. The relative expression level was calculated using the method of 2^−△△Ct^. Values are means ± SD (*n* = 3). * represents significance at *p* < 0.05 compared with the reference

The same method was utilized to treat zucchini seedlings with 20% PEG (Fig. 9, Table S4, Table S5). It was found that the most significant genes were expressed at 3 h (*CpARF07, CpARF11, CpARF12, CpARF15, CpARF16, CpARF18, CpARF20, CpARF23, CpARF25, CpARF26, CpARF28, CpARF30, CpARF31, CpARF32*), *CpARF05*, *CpARF14,* and *CpARF17* were the most significant at 6 h, and *CpARF02*, *CpARF10*, *CpARF21,* and *CpARF33* were the highest at 9 h. The expression level of *CpARF03*, *CpARF08,* and *CpARF09* was the most significant at 12 h, and the expression level of *CpARF22* was the highest at 24 h. Only *CpARF24* was down-regulated under drought stress. *CpARF06* was almost not expressed before and after treatment, while the expression trend of *CpARF01* and *CpARF22* were first down-regulated and then up-regulated. Moreover, the expression trends of 9genes (*CpARF02, CpARF12, CpARF14, CpARF16, CpARF21, CpARF23, CpARF27, CpARF29CpARF31*) were first up-regulated and then down-regulated, and the expression trends of 12 genes (*CpARF05, CpARF11, CpARF15, CpARF17-CpARF20, CpARF26, CpARF28, CpARF30, CpARF32*) were first up-regulated, then down-regulated and then up-regulated. Eight genes (*CpARF03, CpARF04, CpARF07-CpARF09, CpARF13, CpARF25, CpARF33*) were up-regulated first, then down-regulated, then up-regulated, and then down-regulated. The expression tendency of *CpARF10* was up-regulated first, then down-regulated, then up-regulated, then down-regulated, and finally up-regulated. Of concern, most of these *CpARF* genes were up-regulated under different treatments, indicating that they responded positively to drought stress.

### Expression analysis of ARF Gene in leaves of Zucchini in response to pathogen stress

**Fig. 10:**
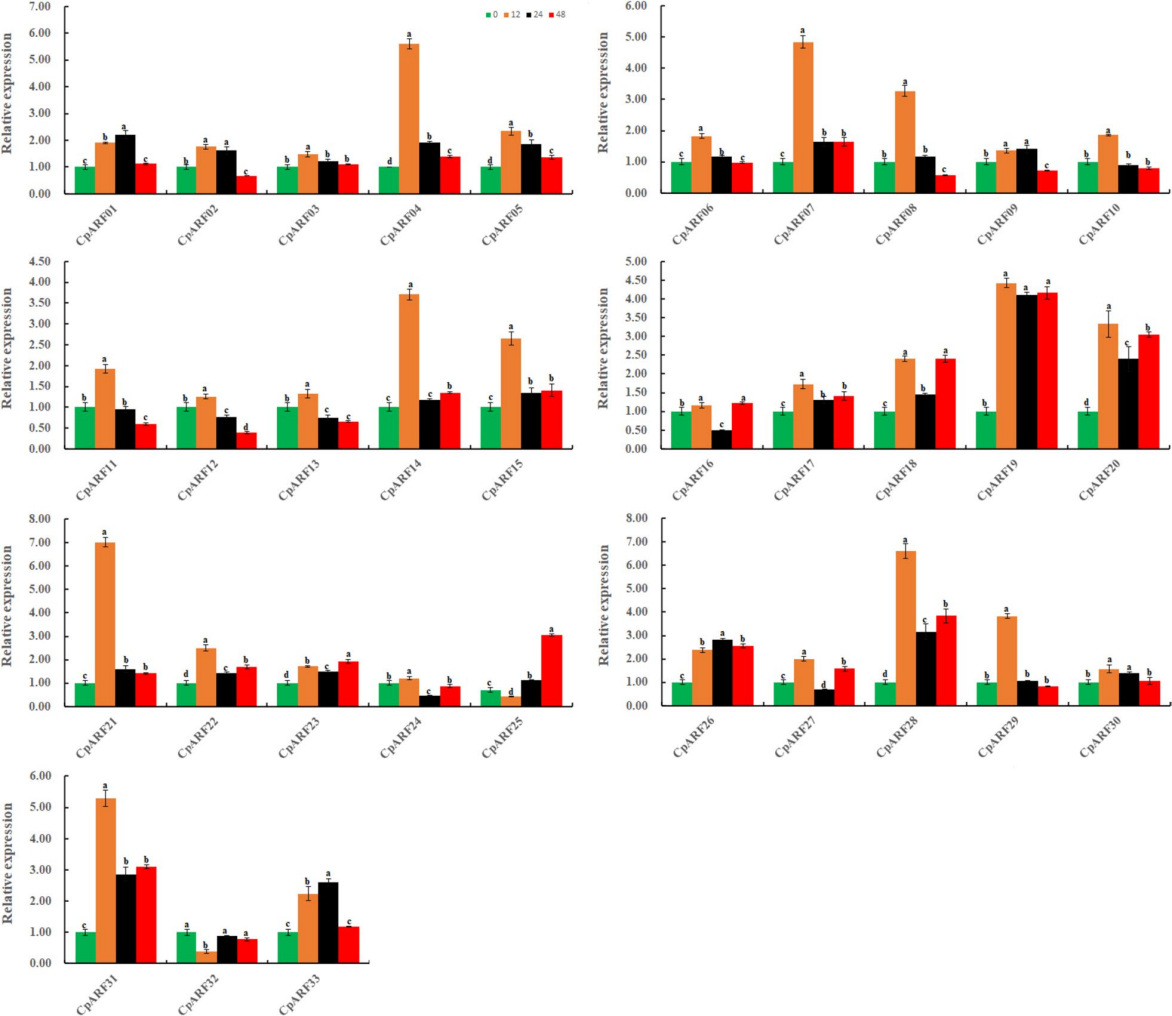
The auxin response factor (ARF) gene in the leaves of the zucchini responded to pathogen expression profiles. The relative expression levels of 33 *ARF* genes in the leaves of zucchini were measured at 0,12,24,48 h after treatment. The relative expression was calculated using the method of 2^−△△Ct^. Values are means ± SD (*n* = 3). * represents significance at *p* < 0.05 compared with the references

In order to analyze the expression patterns of ARF genes in zucchini after infection by pathogenic bacteria (Fig. 10, Table S4, Table S6), the zucchini growing for about 15 days was inoculated with* F. oxysporum* isolated in our laboratory, and the leaves were taken at different time points (0,12,24 and 48 h) for qRT-PCR analysis. The results showed that the expression of 25 ARF genes in the leaves of zucchini increased significantly at 12 h in the initial stage of inoculation, and decreased to varying degrees at 24 h or 48 h after inoculation. Four ARF genes (*CpARF01, CpARF09, CpARF26, CpARF33*) had the most significant expression at 24 h, and four ARF genes (*CpARF16, CpARF18, CpARF23, CpARF25*) had the highest expression at 48 h. The expression pattern of 18 genes (*CpARF01-CpARF13, CpARF21, CpARF26, CpARF29, CpARF30, CpARF33*) was up-regulated first and then down-regulated. The expression pattern of 14 genes (*CpARF14-CpARF20, CpARF22-CpARF25, CpARF27, CpARF28, CpARF31*) was up-regulated first, then down-regulated and then up-regulated. The expression pattern of *CpARF32* was down-regulated first, then up-regulated, and then down-regulated.

### Expression Analysis of *ARF* Genes in Roots of Zucchini in response to pathogen stress

**Fig. 11:**
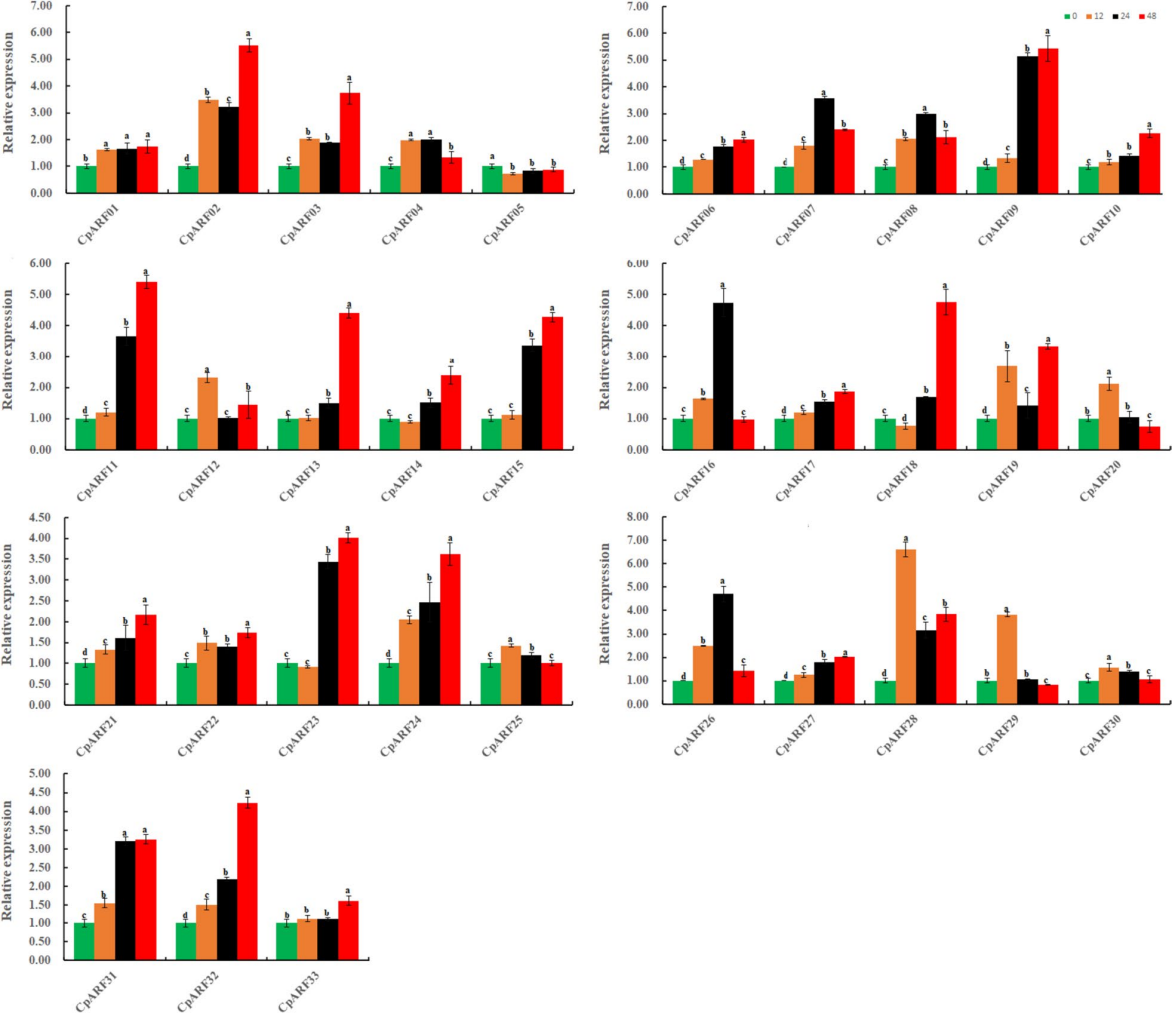
The auxin response factor (ARF) gene in the roots of the zucchini responded to pathogen expression profiles. The relative expression levels of 33 ARF genes in the root of zucchini were measured at 0,12,24,48 h after treatment. The relative expression was calculated using the method of 2^−△△Ct^. Values are means ± SD (*n* = 3). * represents significance at *p* < 0.05 compared with the references

In the root, the expression level of the *CpARF05* gene decreased after inoculation with *F.oxysporum*, and other genes increased significantly (Fig. 11, Table S4, Table S6). The expression level of six genes (*CpARF12, CpARF20, CpARF25, CpARF28, CpARF29, CpARF30*) was the most significant at 12 h, and the expression of five genes (*CpARF04, CpARF07, CpARF08, CpARF16, CpARF26*) was the highest at 24 h, and the expression of the remaining 21 genes was the most significant at 48 h. Among them, the expression pattern of 14 genes (*CpARF01, CpARF06, CpARF09-CpARF11, CpARF13, CpARF15, CpARF17, CpARF21, CpARF24, CpARF27, CpARF31-CpARF33*) was continuously up-regulated. Nine genes (*CpARF04, CpARF07, CpARF08, CpARF16, CpARF20, CpARF25, CpARF26, CpARF29, CpARF30*) were up-regulated first and then down-regulated, and four genes (CpARF05, *CpARF14, CpARF18, CpARF23*) were down-regulated first and then up-regulated. Six genes (*CpARF02, CpARF03, CpARF12, CpARF19, CpARF22, CpARF28*) were originally up-regulated, then down-regulated, and then up-regulated. The above results showed that the ARF family genes of zucchini had a rapid response to the infection of *F.oxysporum*, and the expression patterns of various *CpARFs* were different, which indicated that different ARF members of zucchini played different roles in the stress regulation pathway of pathogens.

### Subcellular Localization of CpARF22 Protein

**Fig. 12:**
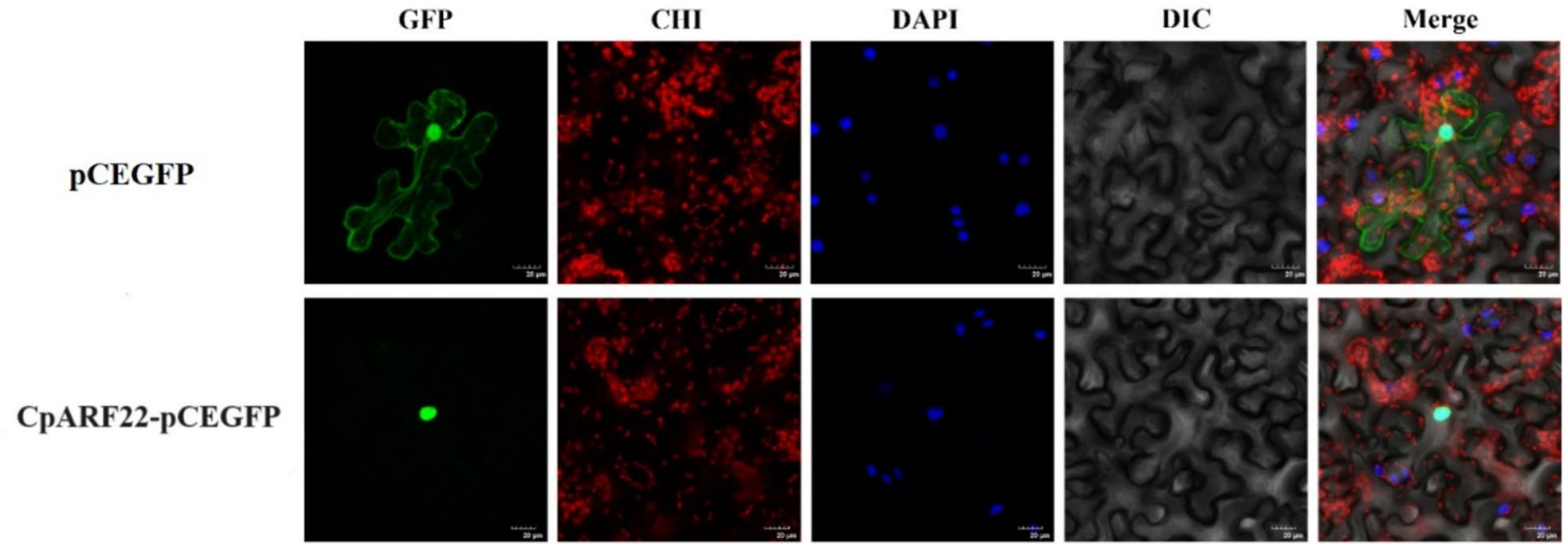
Subcellular localization. Localization of *CpARF22* in tobacco leaves, CpARF22-GFP represents the gene and GFP represents the control is an empty vector. GFP stands for green fluorescence field, DAPI stands for DAPI field (nuclear staining), DIC stands for indicating field, and Merge stands for superposition field. The images were taken by LEICA DMi8, Japan fluorescence microscopy

The chimeric *CpARF22* gene DNA in *Agrobacterium tumefaciens* was immersed in tobacco leaves, and the fluorescence number in the cells was observed by a laser scanning confocal microscope. As shown in Fig. 12, the CpARF22-GFP fusion protein is expressed in the nucleus.

### Overexpression of* CpARF22* Increased Salt and Drought Stress Tolerance in *Arabidopsis thaliana*

**Fig. 13:**
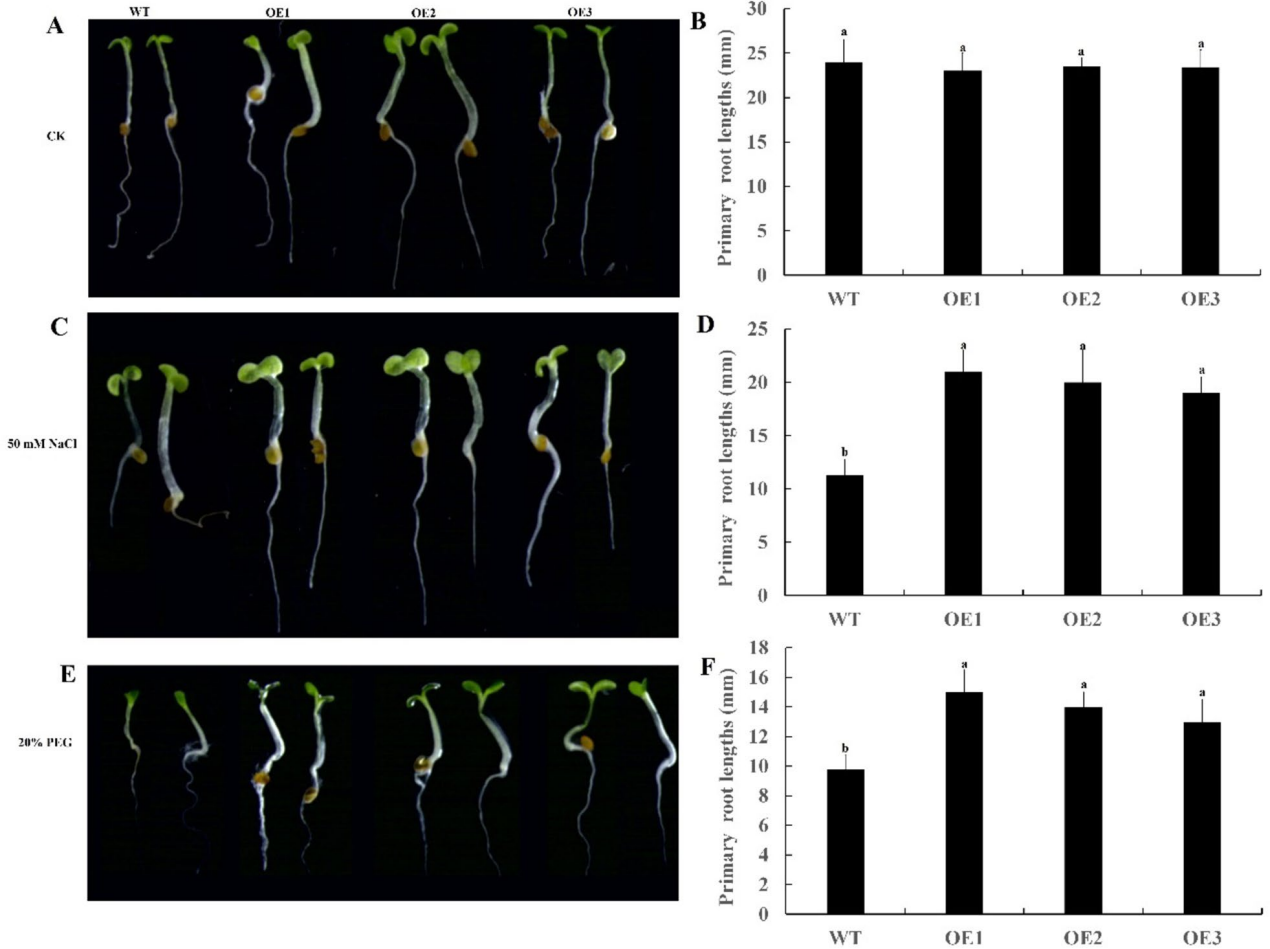
Effects of salt and drought stress on root length of wild-type (WT) and *Arabidopsis thaliana* overexpressing *CpARF22* (OE-1, OE-2, and OE-3)

In order to further study the function of *CpARF22* gene, we successfully cultivated transgenic *Arabidopsis thaliana* plants over-expressing *CpARF22* through *Agrobacterium tumefaciens*-mediated transformation and obtained a homozygous T_3_ generation over-expression lines (OE1-3). Randomly selected transgenic and wild-type *Arabidopsis thaliana* seeds were seeded on normal 1/2 MS medium, 50 mM NaCl and 20% PEG 1/2 MS medium, respectively, there are no significant differences were shown between WT and OE line plants under normal conditions (Fig. 13A, B). However, in 50 mM NaCl and 20% PEG treatments, we observed significant differences in growth between transgenic and WT plants After growing on 1/2 MS medium containing 50 mM NaCl and 20% PEG for 1 week, the root length of *CpARF22*-overexpressing plants exhibited a longer root phenotype compared to WT (Fig. 13C, D, E, F). Taken together, these results suggested that over-expression of *CpARF22* enhances salt and drought tolerance in transgenic *Arabidopsis thaliana*.

## Discussion

Auxin response factors (ARFs) are a class of transcription factors widely involved in plant growth and development and stress resistance regulation, so they become a key gene family for understanding plant biology. At present, there is not any detailed analysis of the ARF genes in zucchini. With the continuous exploration of the nutritional value of zucchini, its economic value is also constantly improving. In order to better elucidate the function of ARF in affecting specific auxin responses in zucchini, we carried out a systematic and comprehensive analysis of the ARF family genes in zucchini.

In this study, we identified 33 *CpARF* genes by analyzing the whole genome of zucchini, the number of genes was close to that of maize [32] and *Populus trichocarpa* [33], lower than that of soybean [34] and Osmanthus fragrans [35], higher than that of *Arabidopsis thaliana* [36] and rice [37]. Subcellular localization prediction showed that the CpARF proteins of zucchini were mostly located in the nucleus and cytoplasm. We constructed a phylogenetic tree with 101 ARFs based on zucchini, *Arabidopsis thaliana* and pumpkin. According to the phylogenetic tree analysis, the CpARF genes were divided into seven branches. At the same time, 23 pairs of orthologous genes and 1 pairs of paralogous genes were found, which meant that many species expanded through their species specificity in the evolution of ARF genes [38]. Studies have shown that gene duplication may be one of the fundamental factors in the genetic innovation of the zucchini genome. In this study, 33 *CpARF* genes were unevenly distributed on 16 chromosomes. Most of them were located on chromosome 1, and only one group of tandemly repeated *CpARF* genes (*CpARF26, CpARF27*) were found on chromosome 16. Gene structure is usually conserved during evolution, this study systematically analyzed the structural distribution and length of exon–intron of the ARF genes in zucchini. It was concluded that the member of the ARF genes in zucchini were composed of 3–16 exons, and similar results have been observed in some species, such as *Arabidopsis thaliana* [39], rice [40], tomato [41]. The above results showed that the ARF genes is highly conserved in structure. Changes in intron during evolution can be considered a necessary way for gene families to obtain novel gene functions [42]. In addition, ARF proteins belonging to the same taxonomic branch show common evolutionary origins and have conserved motifs associated with their functions [43]. These specific conserved motifs ensured the characteristics of ARF protein interaction and DNA binding modification [44]. In general, the structural relationship between CpARF proteins can indicate their functional similarities and differences.

Cis-acting elements, as key molecular switches, are involved in regulating the transcriptional regulation of gene activity in various biological processes. They regulate the precise initiation and transcription efficiency of gene transcription by binding to transcription factors [45]. *ARF* genes are widely involved in regulating plant development and various stress responses, we assessed the cis-acting elements in the promoter region of *CpARF* family members. Several cis-acting elements responding to plant hormones were found, including abscisic acid response element (ABA), gibberellin response element (GA), jasmonic acid response element (JA), salicylic acid response element (SA) and auxin response element (IAA). This finding suggests that gene members of the *CpARF* family may be involved in regulating plant development and plant stress responses. In addition, we also found cis-acting elements in response to light and pressure, indicating that the *CpARF* gene may be affected by light and pressure during the development of zucchini. The *CpARF* gene also contains cis-elements related to defense, stress response, zein metabolism regulation, and endosperm expression.

At present, many studies have shown that *ARF* genes have potential regulatory effects under various abiotic stresses [46]. In oil palm, six oil palm *ARF* genes, including *EgARF9*, *EgARF10*, *EgARF12*, *EgARF13*, *EgARF15,* and *EgARF22*, were significantly up-regulated under drought and salinity stress conditions, the over-expression of these six genes improved the resistance of oil palm to drought [47]. In addition, *NsARF1b*, *NsARF7b*, *NsARF9b,* and *NsARF16* were significantly up-regulated under drought stress in *Nitraria sibirica*, and the up-regulated *NsARFs* had little correlation with ABA biosynthesis [48]. In this study, we used qRT-PCR to examine the expression profiles of zucchini genes under drought and salt stress conditions. The results showed that 94% of *CpARF* genes were significantly up-regulated under salt stress, and 88% of *CpARF* genes were significantly up-regulated under drought stress, indicating that these genes may have different response mechanisms to drought and salt stress, and they may play a central role in abiotic stress processes. Some studies have reported that ARF are involved in resisting pathogen infection [49]. Fusarium wilt is the major limiting factor affecting the production of zucchini. In addition, we found that the expression of the *CpARF* gene was up-regulated mainly in response to *F. oxysporum* infection, indicating that zucchini ARF seems to play an active role in resisting pathogen infection. In summary, this paper summarizes the ARF gene of zucchini and provides a reference for further study of the function of zucchini under pathogen stress and abiotic stress. This study is only the beginning of the study of ARF in zucchini, and the functions of various candidate genes have not been verified. In the future, we will further elucidate the gene functions of these candidate genes under pathogen stress and abiotic stress. And further, clarify the molecular mechanism related to the disease resistance of zucchini. In addition, the role of ARFs in the growth and development and fruit ripening of zucchini can be further studied.

## Conclusion

In this study, systematically analyzed the related information, phylogeny, gene structure, conserved motifs, chromosome localization, gene replication, cis-acting elements, and protein interactions of the ARF gene family in zucchini. In addition, we analyzed the expression patterns of zucchini under two different stress treatments and zucchini fusarium wilt infection. The cis-element found in the *CpARF* gene promoter was considered to be associated with plant hormones abiotic stress and pathogenic stress. QRT-PCR analysis showed that under salt stress and drought stress, the expression of *CpARF08* gene was down-regulated most obviously at different time periods. Under pathogen stress, the expression of *CpARF21* gene in leaves was down-regulated most obviously at different time periods, and the expression of *CpARF28* gene in roots was down-regulated most obviously at different time periods. The transgenic *Arabidopsis thaliana* lines with the *CpARF22* gene enhanced their tolerance to salt stress and drought stress.

### Supplementary Information

**Additional file 1:  Table S1.** The 33 ARF gene-coding protein sequence information in this study.

**Additional file 2: Table S2. **Amino acid sequence information of 101 ARF genes.

**Additional file 3: Table S3. **Cis-acting elements in the promoter region of CpARF genes.

**Additional file 4. Table S4. **The primer is designed for qRT-PCR. 

**Additional file 5: Table S5. **Relative expression of 33 genes by qRT-PCR in different stress.

**Additional file 6: Table S6. **Relative expression of 13 genes by qRT-PCR in Pathogen stress.

## Data Availability

The reference genome assembly used for data analysis was obtained from the National Center for Biotechnology Information (NCBI). The datasets analyzed during this study are included in this published article and its supplementary information files.

## Abbreviations

qRT-PCR: Quantitative RT-PCR
*CpARF*: *Cucurbita pepo Auxin Response Factor*
UTR: Untranslated region
GFP: Green fluorescent protein
