# Supplementary material for: Identification of ARF genes in Cucurbita pepo L and analysis of expression patterns, and functional analysis of CpARF22 under drought, salt stress
Source: BMC Genomics. 2024 Jan 25;25:112. doi: 10.1186/s12864-024-09992-8 (PMC10809590; doi:10.1186/s12864-024-09992-8)
Supplement: Supplementary file 1 — Additional file 1: Table S1. The 33 ARF gene-coding protein sequence information in this study. [file 12864_2024_9992_MOESM1_ESM.docx]

Table S1 Amino acid sequence information of 33 ARF genes

>CpARF01

MANRGGGSFSPSNVSSQGSGRDGLYVELWKASAGPLVEVPRVHEHVFYFPQGHMEQLEASTNQELNQKLPSFNLPSKILCHVVDVRLLAEQETDEVYAQISLIPEGNQEEPATPGPSPGECRKSKVHSFCKVLTASDTSTHGGFSVLRKHATECLPPLDMTQQTPTQELIAKDLHGYEWRFKHIFRGQPRRHLLTTGWSTFVTSKRLVAGDSFVFLRGENGELRVGVRRLSRQQSTMPSSVISSHSMHLGVLATASHAAATLTRFVVYYKPRASQFLVSLSKYKEAMNTKFLVGMRFKMRFEGEDSPERRFSGTIIGVDDISPYWPNSQWRSLRVQWDELASIQRPERVSPWEIEPFVAPVSQSIPQSISVKNKRPRPPLDIPESDNSTVTTLRHPVSTQPHDDKTQLSVPAGWHYKQTDISSNGNSVSRTHNEGSWLTVPNGSVSQHRLKDSTEDHKSNSVWSAVFPGVPTAHSTCPTPRTSNPMSDQINEFGEEGRKTEVAPSCRLFGIELIDHSKRPVPPERTSDQSNCAANEVTESHVNTLLSSNAEQQGSNLPKASSKERKLGLLQALPKEIQHKHNASTNCRSRTKVQMQGMAVGRAVDLTTLVGYDQLIDEMEKMFDIKGELRPRDKWEIVFTDDEGDTMLMGDYPWQEFCNMVRRIYIWSSQDVKTSSGRRLTMSAIEFDGTAITSESADS

>CpARF02

MSFSAASNLPSGGPHSGSSSDALYRELWHACAGPLVTLPRQDERLEASMNQGLEQQMPSFNLASKILCKVVNVVLRAEPDTDEVYAQITLLPESNQSEVTTPDPALPEPTRCNVHSFCKTLTASDTSTHGGFSVLRRHADDCLPPLDMSQQPPWQELVATDLHGNQWHFRHIFRGMVYKVEQLLYLTPSQLFAFAIALLIYLEQLANSRFLNILPYDRTSRSTFLVSLNKYLEAQNHKLSVGMRFKMRFEGEEVPERSFSGTIVGLGDNASPGWANSDWRSLKVQWDEPSSILRPDRVSAWELEPLVASNPLSSQPTQRNKRPRPTVLPSPTADAAVLGGWKPTVESSAFSFTEPPQRGRDVYSSPKFSTAASNSLGFNGNSSLGAVSSNNYWSNTNRVENIIDTSSHGANREPIEKKQNTKNGCRLFGIQLLGNSDVGEASPDTTPKMVSVDRKLEEMFDIEGELCGSVKKWQVVYTDDEDDMMMVGDDPWNEFVSMVRKIFIYTTEEVKRLSPKIKLPLGGETKLSKPDSDTTANHTEDQSSIVGSDC

>CpARF03

MLSIESDWVKLDMKLSTSGFGQQDHEGGEKKCLNSELWHACAGPLVSLPTSGTRVVYFPQGHSEQVAATTNKEVDGHIPNYPNLPPQLICQLHNVTMHADVETDEVYAQMTLQPLTAQEQKDTFLPMELGIPSRQPTNYFCKTLTASDTSTHGGFSVPRRAAEKVFPPLDFSQQPPAQELIARDLHDVEWKFRHIFRGKSPYVRNEKNQLLLGIRRATRPQTVMPSSVLSSDSMHIGLLAAAAHAAATNSCFTVFYNPRASPSEFVIPLTKYVKAVFHTRVSVGMRFRMLFETEESSVRRYMGTITGISDLDPVRWSNSHWRSVKFSVSFSTVKVGWDESTAGERQPRVSLWEIEPLTTFPMYPSLFPLRLKRPWHPGVSSVHDNRDDASNGLMWLRGGVGEQALHSLNLQSAGSLPWLQQRLDSSMFGNDHNQQYQAMLAAGMPNLGGVDMLRQQIMHLQQPFQYAQQAGLHNSLLQLQQHQLVQQSMSQNILQAPSQVMAENLPQHILQQTLQNQPEDLPNQQQHTYHDTVQVQSNQFHQGGHSNVPSPTFPRSNLVDSNSNYPESISSRRNIIASSCAEGTGNLSNIYRSGQSILTEQLPQQSPVSKNAHSPVDAHPNSMSFPFSGRDSILELGNCNSDSPSPTLFGVNIDSSGLLLPSNVPTYASPSIAPDSTSMPLGDSGFQNSLYNCVQGSSELLHSSGQVDPSNPTQTFVKVYKTGSIGRSLDISRFSSYQELREELAQMFGIEGQLEDPRRSGWQLVFVDRENDALLLGDDPWEAFVNNVWYIKILSPQDVQELREQVIESFNPIDGQRLTSGSNEAENVSGLPSVGSLEY

>CpARF04

MNRGMDSVKEPMRNVEKCLDSQLWHACAGGMVQMPSVNSKVVYFPQGHAEHAQGNVDFGNARIPSLIPCRVSATKYMADLETDEIFAKISLLPLRNNEFNLDDDYELLGHDDIRNQEKPSSFAKTLTQSDANNGGGFSVPRYCAETIFPPLDYSAEPPVQTILAKDVHGEIWKFRHIYRGTPRRHLLTTGWSNFVNQKKLVAGDSIVFLRAETGDLCVGVRRAKRGIGCGIDYSPGWNPANSGSSLMGYSDFMRENEGRMVRRHSNGNLNGRVKVESVIEAATLAASGKRFEIVYYPCAGTPEFVVRASTVRSAMQIHWYSAMRFKMPFETEDSSRISWFMGTISSIQVADPVRWPDSPWRMLQVSWDEPDLLQNVKSVNPWLVEVVVNMPAIHVSPFLPPRKKPRYPLQAETAVFGHLPMPSFSTNFFEATNPFQSITASNIPAGIQGARHTQFGLSSSNLQISIPPLRQFPAGLKHLEGATPIPVVRGEDSFGGTKSPDNQSHWLTVGNHIQSSKETKETKPDHIILFGQLILPNQQSSNSSSGDTTNASDANLEKASNLSDGSGLSSQQNGSLDNSSDGGSTSYNGPNKTGLSLDIGHCEVFMELENVSHTLDLSVLGSYEELYRKLGNMFSVGRSEMLNSVLYQDALGATKQAGDEPFSKFMRTARRLTILANSRSRFSKKRQY

>CpARF05

MTSAEASIKPNSASAFKNHADSTKAPSDPPNALSAKDADIALYTELWNACAGPLVSVPRENERVFYFPQGHIEQVEASTNQVPDQQMPVYDLPSKILCRIINVCLKAEADTDEVFAQITLLPEANQDEDSVDKEPPPPPPRRFHVHSFCKTLTASDTSTHGGFSVLRRHADECLPPLDMSQQPPTQELVAKDLLGNEWRFRHIFRDANGSKFMFRTSPSEFVVPYDQYMESIKKSYTIGTRFKMRFEGEEAPEQRFTGTVIGCEDADPKRWKDSKWRCLKVRWDETSTISRPEKVSPWKIEPALAPPALNPLPMTRPKRHRSNMVPTSPDSSVLTREGPSRMIIDHSPASAFTRVLQGQEFSTLRGNLIDGNESDTAEKSVMWSPSLDDEKIDVVSTSKKHGADSWMPAGSGEPTYADLLSGFGANIDSSCGVRATLGDPAIVTANSIRKHAVYQDGKFNFLGGGSSWSVLPSGLSLNLVDSSQKAHIQAGDLPYQMRGNATFNGVGDHSMAQCYRIDHSMAHCSRIEQPNGNWSMPPPSSHFDYPVHSTELTSKPMLFQNQDILNLKDGNCKLFGISLVKNPAISDPAELHRNVMNEADVIHANRHPIHLHESELSRGSNLADKSLAINVADKLQHACTPNLKDSQSKSQGSSTRSCTKVHKQGIALGRSVDLSKFNNYDELIAELDQLFEFGGELLPPKKSWLVVYTDDEGDMMLVGDDPWQEFCGMVRKIFIYRREEVQKMNPRSLTLKGDENPSVEGEEAKEIKSPAVPSMSAPES

>CpARF06

MGSVDEKLKTIGGFITHAPQTNLLDDMKLLKEMQDQTGARKAINSELWHACAGPLVSLPHMGSLVYYFPQGHSEQVAVSTKRTATSQIPNYPNLPSQLMCQVHNVTLHADKDSDEIYAQMSLQPVNSEKDVFIVPDFGLRQSKHPNEFFCKTLTASDTSTHGGFSVPRRAAEKLFPPLDYTMQPPTQELVVRDLHDNTWTFRHIYRGQPKRHLLTTGWSLFVGAKRLRAGDSVLFIRDEKSQLLVGVRRANRQQSMLPSSVLSADSMHIGVLAAAAHAAANRSPFTIFYNPRACPSEFVIPLAKYRKCVYGTQLSAGMRFGMMFETEESGKRRYMGTIVGISDLDPLRWPGSKWRNLQVEWDEPGCCDKQNRVSSWDIETPESLFIFPSLTSGLKRPLHGGFLGETDWGSLVKRPMLRVPENVSYASTLCSEPLMKMLLRPQLVNLHGTTLQQQDMMGVMQPVTNPKIQQQVTPSSQHQNHHQPAPSPSTDPINSNSSPKANEPGKVQDPAAIESEAPTGAEADKSKYEREVSTCQSNPLPPVDCGEDKLTGNEVNMQTLVNQLSFVNQNQIPMQLQSVSSWPMQPQLESLIQHPQPIDMPQPEFPNSNGLISSMDGDGCLINPTCLPLSGVMRSPGNLSMLGLQDSSTVFSEALNFPIPSTGQEMWDPLSNIRFSSQNNHLVSFSHPDASNLNCMANANVTRDVSDESNNQSGIYGCSNLEISNGGNTLVDPAVSSTILDDYCTLKDADFPHPSDCLAGNFSSSQDVQSQITSASLGDSQAFSRQEFHDNSGGTSSCNVDFDEGNLLQNGSWKQVVPPMRTYTKVQKAGSVGRSIDVTSFKNYEELCSAIECMFGLEGLLNDPRGSGWKLVYVDYENDVLLIGDDPWEEFVSCVRCIRILSPSEVQQMSEEGMKLLNSAMMQGMNCSISEGGRA

>CpARF07

MTFHGGDSYTQSCVSAQGRGRDDLYMELWRACAGPLVDIPRVDEKVFYFPQGHMEQLEASTNLELNKRIPLFNLSSKILCRVIYIEPRADHESDEVYAQITLLPEANQSEPRSLDPCPPEPVKPVVHSFCKVLTASDTSTHGGFSVLRKHANECLPPLDMTQATPTQDLVAKDLHGYEWRFKHIFRGQPRRHLLTTGWSTFVTSKRLTAGDSFVFLRGDNGQLRVGVRRRAQQQSSMPPSVISSQSMHLGVLATASHAVTTQTRFVVYYKPRTCQFIISLNKYLEAVNYKFSLGMRFNMRFEGEDSPERRFSGTIIGAGDISPHWANSSWRSLRVSYSYSIPHREHHYGADLTKSAHWDSGLAQSHDGKQCGNATESRKGENNELCHHSEIDTISNSTCVSRTPTDGTWLFSTQSNGYKDPVNDTAQDYKTVPVCGWSILSGPSKVSDDQILDPTENGWKGETVASCRLFGIDLNHFAKLPAEKASSQPSSMSSDTDGRISTLSLAQSDSKSDNIEASIERKLEPLQASLKETQSSSTNTRSRTKVHMHGMAVGRAVDLTILEGYDQLIDELEKMFDVRGQLSARGKWEIVYTDDEGDMMLVGDDPWEEFCNMVRRIFICSREQVKKMSSGSKQLASIEVEGAVVIPDSPAI

>CpARF08

MFIALHLGENAIIRMKEADKNLDPQLWHACAGGMVQMPAINSKVFYFPQGHAEHAQAAVDFTSSLRIPPLIPCRVLAVKFLADHETDEVYANVRLVPLANNEINCEEEGGFGSNGSENNMEKPASFAKTLTQSDANNGGGFSVPRYCAETIFPRLDYTADPPVQTVIAKDVHGEVWKFRHIYRGTPRRHLLTTGWSSFVNQKKLVAGDSIVFLRSKNGDLCVGIRRAKRAIGCAADHPYGWNPGGGNSIPPYGGLTMFLRDEDNKLSRKGSLSSNGGGSLRGKGKVRPESVIEAAALAASGQAFEVVYYPRASTPEFCVKASSVRAAMRIQWCSGMRFKMPFETEDSSRISWFMGTISSVQVADPIRWPNSPWRLLQVTWDEPDLLQNVKRVSPWLVELVSNMPVIQLSPFSPPRKKFRLPQHPDFPLDSQFQLSSFPSNTLRPSSPMCCLSDNTSVGIQGARHTQFGISSSDFHLNNKLQLGLLPSSFQQLDFHSRISHRSVTDHRGSGSQNSSALLNAENTGPKLERSDSVKKYQFLLFGQPILTEQQITRSSSGDVHSPRTEKSSLDVNLERVKFLSDGSGYTFKQQQISPNKSPGAGFPWYQGYQATELGLDIGHCKVFMESEDVGRTLNLSVIGSYEELYRRLANMFGMEKPDILSHVLYQDATGAVKQAGDKPFSDFIKTARRLTILTDSGSDKMVGRRLMDGLRSGENGLDSSNKTGPLSIFA

>CpARF09

MEIDLNLTASDVGKNAYCNGNCEEGRCNYCLSSSTSSCSSNSSPALVSSSTYLELWHACAGPLTSLPKKGNVVVYFPQGHLEQIASASPFSPMEMRTFDLQPQILCRVINVHLLANKENDEVYTQLTLLPLPEFLGTGLEGKELEELALNGADGDGSGVSPTRSTPHMFCKTLTASDTSTHGGFSVPRRAAEDCFPPLDYTQLRPSQELIAKDLHGVEWRFKHIYRGQPRRHLLTTGWSLFVSQKNLISGDAVLFLRGENGELRLGIRRAVRPRNGLPDSIVGNQNSCADDLARVVKAISTKSIFDVFYNPRAYHAQFVISCQKYVKSINNPVTVGTRFKMRFEMDDSPERRFNGVVLGIGDMDPLRWPNSKWRCLTVRWDKDGDHQERVSPWEIEPSVSLPPLSVQSSPRLKKLRTSLQAAPPNAFAVGRGGFMDFEDSIRSSKVLQGQENVGIVSPFYGRDTTKRSLEFEVRSSAHQASGGAEKYRGDYVKVHPNSSFTGFMESERFLKVLQGQEICSLRPQTRKPEHCLGVWGKFNLSDNSFNTFHSPNSSFYHMASNGAQNMYFPRSEFYSTGQAAAVMRSNDGNFPRESALFSPSVDASVMSTTSGSNIKNSKDENVNENSTGCRLFGFSLTTETATNMQSSGKRSCTKVHKQGSLVGRAIDLSRLSGYTDLLSELERLFCMEGLLKDPDKGWRVLYTDNENDVMVVGDYPWLDFCDAVSKIHIYTQEEVEKMTNGVISDDTQSCLDQAPLCMEASKSSSVGQPDSSPTVGAAHEYRL

>CpARF10

MSSVEDKLKTPMGLVNNTPQTNLLDEMKLLKQMQDQTGARKAINSELWHACAGPLVSLPHVGSLVYYFPQGHSEQVAVSTKRTATSQIPNYPNLPSQLMCQVQSVTLHADKDSDEIYAQMSLQPVNSEKDVFLVPDFGLRPSKHPNEFFCKTLTASDTSTHGGFSVPRRAAEKLFPPLDYTMQPPTQELVVRDLHDNTWTFRHIYRGEDEKSQLLIGVRRANRQQTTLPSSVLSADSMHIGVLAAAAHAAANRSPFTIFYNPRACPSEFVIPLAKYRKCVFGTQLSAGMRFGMMFETEESGKRRYMGTIVGISDLDPLRWPGSKWRNLQVEWDEPGCCDKQNRVSSWEIETPESLFIFPSLTSGLKRPLHGGFLGETDWESLVKRPMLRVPENIRGDLSYAAPNLCSEPLMKMLLRPQLVSLHGATLHQDSANNLVKIQDMKDMQPETNSKMKQPIPTETASPGNQNPDPINPNSSPKESLPGKVHTSAATESEPPTGADDVSTNQSNTLPPVGDCVGGKLSAKEVNMQTLVNQLSFMNQNQIPMQSWPLQPQLESLIQHPQPIDMPQPEFTLDGDGCLINPSCLPLPGVMRSPGNLSMLALQESSTVFPEALNFPLPSTGQDMWDPLNNIRFSSNLNCMGNANVMRAVSDESNNQSGIYSCSNLEISNGGNTLVDPAVSSTILDDYCALKDADFPHPSDCLVGNFSSSQDVQSQITSAASLGDSQAFSRQDFHDNSGGTSSCNVDFDEGSLLHNGSWNQVVPPMRTYTKVQKAGSVGRSIDVTSFKNYDELCSAIECMFGLEGLLNDPRGSGWKLVYVDYENDVLLIGDDPWEEFVSCVRCIRILSPSEVKQMSEEGMKLLNSAMMQGINCSMPEGGRG

>CpARF11

METNFLFFLLLFDHRRSDSGSMDRQLWQAFAGKSVHIHTVGSEVYYFVQGHVEQATYIPSLSQSVLSNPATKCLVTGTDFHADSLSDEVCIKLNLHPIRPGRDESVVVSRFGRCDDNGGERDKIESFAKILTSSDANNGGGFSVPRCCADSVFPPLNYQADPPVQTLSITDVHGVVWNFRHIYRGTPRRHLLTTGWSKFVNHKKLIAGDSVVFAKNLRGDMFVGIRRASTSITRAISGGDCGRWNSQNGGARCSLEENCSGDGGTKVFSRRNIGKLPAEAVANAAELAAQFKPFELVYYPRAGRSEFVVQVEKVNKSKNYQWYSGTRVKMAMETEYSKMTWYQGTVTSASVPEHGPWMGSPWRMLEVTWDEIDALQSAKYVSPWEVELATPTPPIQPPLHPAKKFRGLPKSGMLNGEAELFSPLMNFVDSTMEQFNPSLLNLNSFPAGMQGARQKLFCESRLFNPCKEMTPLTCDESSMSELNAAKTMQMVSTDLLIGSVQFDTLSSDSQASVLSFATGTAENQNCCNSAKAGVNSIQLFGQVIYMNPPVENGLENGVSTNDGGKNLNQYLGQGRQEADYNMNIGSRYFSPASHSSPVNKEEDDGEDGNKAGDELHGLSLQALINVYREAFLDGDQKTISKVEAKLKNFEREKDGLFQKVSNTSAEITSGKENYIRLQADFDNFRKRSEKERLTVKNNAQKEVIENLLPMIDSFEKARQQIVPQTDKEKKIDVSYQGIYKQFVEVLRSWRISAVAAVGRPFDPSLHEAVAREESQEIKEGIIIQELRRGFLLGERLLRPARVKVSKGPGKKNSPTTNSDIPTEQPATATARLDEH

>CpARF12

MKTPANGVASSAAAPNSCEGGLEKKIINPELWQACAGPLVNLPPAGYHVVYFPQGHSEQVAASLKKDVDAQIPNYPNLPSKLLCLLHNVTLHADPETDEVYAQMTLQPVPSFDKDALLRSDLALKSNKPQPEFFCKTLTASDTSTHGGFSVPRRAADKIFPPLDFSMQPPAQELVAKDLHDTVWTFRHIYRAAAHAAANNSPFTVFYNPRASPSEFVIPLAKYYKAVSANQISLGMRFRMIWKGSQWRNLQVGWDESTGGERRNRVSIWEIEPVIAPFFICPPPFFRSKRPRQPGMPDDESSDLDSIFKRTMFGDDFCMKDPQGYPGLNLVQWMNMQNPSLSNMQPNYMHSFSGSMLPNLGGVDISRQLGMSNAQNPQSNNIQFNAQRLLPQAQQLDQLPKIPSTMNSMASVVQPPQQFDDISQQTRQNVVTSQIQSQIMQQPHANGILQQQTSLQNQQLQRSLPQNMLMQQHQQILGQNQQQNMNQPPIPDQSNHPLQMSDNQIQMQMLQKFQQQQQSLLAQQSVLQPAQLAQLPEQQSFSISMSTNQTMDVPHSAPAAGPPPSAQQAPKSHSSNQHLQPKFPQLQQQPHDMSRPMGHNNQFSAATSSVITGAAGAGQSGITDDIPSCSTSPSTNNSPSLVQPVTNGRLHRTTGLVDDVAQSAATIFSTNTLDSISPNANLVKNIPHKAAVKPSLNISKNQSHGVLTQQTFLTGAVPQTEFLDTSSSTTSACLSQNDAQLQQNNIMSFNSQPMLFRDGSQDLEPPTDLHNIPYGTNVDGQLVAHLNSDPLMNKGIGGLGKDFSNNFSSGDMLATYDAQKDPQQEISSSIVSQSFGIPDMTFNSMDSTINDSSFLNRNQWAPPPPFQRMRTYTKVYKRGAVGRSIDITRYSGYDELKQDLARRFGIEGQLEDRQKIGWKLVYVDHENDVLLLKVHSFIYHYGYDSLLAMEEIL

>CpARF13

MKAPPNGFVPNSGEGTGERKNINSELWHACAGPLVSLPPVGSLVVYFPQGHSEQVAASMNKETDFIPNYPNLPSKLVCMLHNVILHADPETDEVYAQMTLQPVNKYEKEALLASDIGLKQNRQPAEFFCKTLTASDTSTHGGFSVPRRAAEKIFPPLDYSMQPPAQELVARDLHDNSWTFRHIYRGMGPFVFSPLLSLDEKSQLLLGIRHANRQQPALSSSVISSDSMHIGILASAAHAAANNSPFTIFYNPRASPSEFVIPLAKYTKAMYTQVSLGMRFRMMFETEESGVRRYMGTITGISDMDSVRWKNSQWRNLQVGWDESAAGERPNRVSIWEVEPVVTPFYICPPPFFRPKFPKQPGMPDDESDIENAFKRAMPWFGDDFGMKDTPSSIFPGLSLVQWMSMQHNNQFPAAQSGILPSMLQQQQLLAGNVQSHQTSQPSNKNSIETTSLLQETQFQPQIDQQPSLVSKHHQQTQFQQAPLQLLQQSLSQRTQQLPQVQQFSQPIIPSEQQLQWQLLQKLQQQQQQQQQQQQQQQPLVSPASPLFPPQMIQPHPVHQQNQQLPPLPLSNQPQFNASGGSFQTEKHNSNGFSSLGLMQSQQVPITQSHNQFKPTTAIRGYSGLTDGDAPSCSTSPSTNNCQVSVSNLLNKNQQGAATLGGDLVVEPATNLPQELPSKPDLRNKPEFPNSKGLDQLKYKGTVPDQSFGVPNLPFKPGCSNDVNVNEAGALSSGLWANHGQRMRTYTKIETKAAKIMKVQKRGSVGRCIDVTRYKGYDELRHDLARMFGIEGQLEDPQRTDWKLVYVDHENDILLVGDDPWEEFVSCVQSIKILSSSEVQQMSLNGNLGHIQAPNQACSGTDSGNAWRGQYDDNSAASFN

>CpARF14

MSFSAVSSLPPGGPTSGPSSDALYRELWHACAGPLVTLPRQDERVYYFPQGHMEQLEASMHQGLEQQMPSFNLPSKILCKVVNVVLRAEPDTDEVYAQITLLPESNQSEVTSPDPALPEPTRCNVHSFCKTLTASDTSTHGGFSVLRRHADDCLPPLDMSQQPPWQELVASDLHGNQTSRSTFLVSLNKYLEAQNHKLSVGMRFKMRFEGEEVPERSFSGTIVGLGDNASPGWANSDWRSLKVQWDEPSSILRPDRVSSWELEPLVASSPLSSLPAQRNKRPRPAVLPSPSADAAVLGGWKPTVESSAFSYTEPQRGRDLYSSPKFSTAASNSLGFNGNNSLGAVSSNAYWSNTSMVENVMDTSSHGAKREPVEKKQNTRNGCRLFGFQLLGNSNVEEASPVSTPKMVGEDRPVPPIDTAFDQHFDPSNIHRSDMPSVSCDADKSCLISPLESQSRQIRSCTKVQMQGIAVGRAVDLTRFNQYDDLLRKLEEMFDIEGELCGSVKKWKVVYTDDEDDMMMIFIYTTEEVKRLSPKIKLPLSGEMKLSKPDSDMVANHTEDQSFSSVRLFFGAFLSPDYKTVQVRSLFLYGLSMTIREEEAQTSSTLL

>CpARF15

MRLSAAGFSPQAPEGERRVLNSELWHACAGPLVSLPAVGSRVVYFPQGHSEQVAASTNKEVDTQIPNYPSLPPQLICQLHNLTMHADADTDEVYAQMTLQPLSAQELKEAYLPAELGTPSRQPTNYFCKTLTASDTSTHGGFSVPRRAAEKVFPPLDFSMQPPAQELIARDLHDNEWKFRHIFRGMKRTNYSSESGVQVDRKPNEKNQLLLGIRRASRPQTVMPSSVLSSDSMHLGLLAAAAHAAATNSRFTIFFNPRASPSEFVIPLAKYVKAVYHTRVSVGMRFRMLFETEESSVRRYMGTITGISDLDPVRWQNSHWRSVKVGWDESTAGERQPRVSLWEIEPLTTFPMYPSPFPLRLKRQWPTGLPSFGIKDSDLGMNSPFMWLRGDNSDRGIQCLNFQGNGVSPWMHPRLDPSMVGMQSDVYQAMAAAALQEMRAIDYSKLAPASMLQFQQPQGLPCQPSTLMQPQMLHQSQSQHAFLQSVQEHQQHSQSQTQTQSHHLQPQLQPEPSFNNQQPQHQQQPRQPQPQPPPQPQPQPLVDHQPIPCTIPAISQFASCSQSQSPSLQTVPSLCQQPSFSDSNGNPVTSPTVSPFHSLAGSFVQDESSQLLNIQRANSVIPSAGWPSKRSAIDPLSTGASQYFLPQVESGMSQNTVALPPFPGRECAIGDRDEGSDPENHVLFGVNIESSSLLMQNGMSNLRGVGNDNVSTTLPFSSNYMSSSGTDFSVNPTTNCIDESGFLQPHENVGQVNQPNGTFVKVHKSGTYSRSLDITKFNNYLELRSELARMFGLEGELEDPLRSGWQLVFVDRENDVLLLGDGPWPEFVNSVWCIKILSPDEVQEMGKRGLELLNSMPIQRLSNNTCDDYGSRQDSRNLISGITSVGPFDY

>CpARF16

MRLSSAGFSPQPPEGERRVLNSELWHACAGPLVSLPAVGSRVVYFPQGHSEQVAISTNREVDAHIPSYPSLPPQLICQLHNVTMHADVETDEVYAQMTLQPLSAQEQKEPYLPAELGAPSKQPTNYFCKTLTASDTSTHGGFSVPRRAAEKVFPPLDFSQQPPAQELIARDLHENEWKFRHIFRGQPKRHLLTTGWSVFVSAKRLVAGDSVIFIWNEKNQLLLGIRRANRPQTVMPSSVLSSDSMHLGLLAAAAHAAATNSRFTIFYNPRACPSEFIIPLAKYVKAVYHTRVSVGMRFRMLFETEESSVRRYMGTITGISDLDSTRWPNSHWRSVKVGWDESTAGERQPRVSLWEIEPLTTFPLYPSPFSLRLKRPWPTGFPSFHGLKEDELGLNSQLMWLRGDGLDRGIQAMNFPGIGVAPWMQPRLDASMVGMQPEIYQAMAAAALQEMRTVDPAKVQAASLLQFQQTQTLPNRPATFMSPQILQQPQPTFIQGDDNQRLSRSQAQTLPTVLQQEIKHQTFNNQQQQQQQVFDHQQIPSTITTMSQFSSASQSQAQSLQTTPPLCRQQSFSDSNPNHVTSPIISPLHSLLGSFPQDESSQLLNLPRTNPMVHSSTWPSKRAAVDPLLSSVNSQFVLPQGENMGTTQTNISQNAISLPPFPGRECSLDQGNADPQSNLLFGVNIEPSSLLMQSGMPNLRGICSDSDSTAIPFSSNYVNTAASNFSANPTGTPSNCIEDSGFLQSPENTGQVNPSTRTFVKVYKSGSLGRSLDISKFSSYHQLRSELAHMFGLEGELEDPVRSGWQLVFVDRENDVLLLGDDPWPEFVNSVWCIKILSPQEVQEMGKQGLELLKSVPIQRLSNGSCDNYANRQESSRNLSTGITSVGSLEY

>CpARF17

MGALIDLNTTEEDESPSSAASSVSSSSASALTSSPSPSLTSSICLELWHACAGPLTSLPKKGNLVVYLPQGHLEQMQEFPATPSDLPPHILCRVIDVQLHQAEAGSDEVYAQVSLLPENEQMEHKMQEEMSNDIEEEDVEEGEKTTTPHMFCKTLTASDTSTHGGFSVPRRAAEDCFPPLDYNQQRPSQELVAKDLLGLKWKFRHIYRGQPRRHLLTTGWSAFVNKKRLVSGDAVLFLRGNDGELRLGIRRAAQLKSGSTFSNICSQKLNSSSIMDVVNAISSKSSFSVCYNPRATSSQFVLPFHKFLKSMNRSFSVGMRFRLSFETEDAADLRHTGHITGIGDVDPIGWPGSRWRSLMVRWDDGEINRHGRVSPWEIEPSGSVSMSSNLVPPGLKRSRIGLSSTKLEFPVPHGIGATDFGESLRFHKVLQGQEILGYNTPTDCDDNTRYHPPEKRRLLPDLHGSGIALMRNGPRNRLINSETSSRGFVFDESFQFQKVLQGQEIFPGPFYGRAMATNEVKESGGCGPVDGFRLSTTRDGWPTTMQCENFHTRSSIPSAQVSSPSSVFMFQQSMVPVLSFDSHNRGIMNKSTSHNSGTAFMTDNSMCPTSLGELNLLGLSHPSTTTASAFSGSKDLSSTTKAGCRLFGFSLTEEKNVGNTDKGSPATTPIHAGTTIGAQCPLKSPLMSKVVGSNCTKGALQYHYANYSTYY

>CpARF18

MDPKKKMITFMDSKEKSKEMDKCLDPQLWHACAGGMVQMPPVNARVFYFPQGHAEHACASPVDFRTCPKLPSYTLCRVSAIKFLADPDTDEVFAKLRLIPINGSELDFDDGIGRLNGSEQDKPTSFAKTLTQSDANNGGGFSVPRYCAETIFPPLDYSADPPVQTILAKDVHGETWKFRHIYRGTPRRHLLTTGWSSFVNHKKLVAGDSIVFLRAENGDLCVGIRRAKRGAGDGPDSSCGWNGAVPYGAFSANESLMGKGKVKAKSVIEAATLAANGQPFEIVFYPRASTPEFCVKAALVKAALQIRWCSGMRFKMAFETEDSSRISWFMGTVNSVQVADPLRWPESPWRLLLNVKRVSPWLVESVSNMSPIHIAPFSSPRKKLRYPQHPDFPLDNQPPMPLFSSYLHGSGSPFGCPPDNNSAGMQGARHAHFGLSLSDFHLSKLHSGLFPIGYRSPDPAAESATLSCNAMTEKPSMSENVSCLLTMAHSTQASKKCDGDDRQEVEPNVDPGHCKVFMESEDVGRTLDLSSLGSYEELYTKLGNMFDIDNSETLNHVLYRDVSGAVKHVGDEQFSDFIKTARRLTIL

>CpARF19

MDPKLWRAFAGDLAHLHTVGSEVYYFVQGHVEQATYAPKLSPAVLSNPVSKCLVTGVGLDADALTDEVLIKINLHPIRPGEGRSEVVSRLGCSEVNVISKFAKVLTSSDANNGGGFSVPRSNRPFTSGECSRVEENPSGDGDPRNFSRRTIGRVPPEVVATAAELAAQFKPFEVVFYPRIGLSQFVVPVEIVNNSMKYQWYPGIRVKLPTETEDTLRTQWHQGTIISVSIPEHDPWKGSPWRMLEITWEETDAPPNGKYVCPWEVELAGPAPPIQPSLHIAKRPRGHSKSGQLNGEAELFSPMMRVGDSSMEQFNQALLSFNSFPAGMQGARQNFLCESGLFDNPYKETTNAEPSTQMVSQVVSTDLHIGSAQSDTLSPDSQASVLSFATESADNQLSNSTEAGVTSFQLFGQIIHLNPPPENGANTDDVDMTSNQSD

>CpARF20

MKTPANGAAAMPNSSEGGLEKKTINPELWQACAGPLANLPPAGYHVVYFPQGHSEQVAASLKKDVDGQVPNYPNLPSKLLCLLHNVTLHADPETDEVYAQMTLLPVPSFDKDALLRSDLSLKSNKPQPEFFCKTLTASDTSTHGGFSVPRRAAEKIFPPLDFSMQPPAQELVAKDLHDNVWTFRHIYRGQPKRHLLTTGWSLFVSGKRLLAGDSVLFIRDEKQQLLLGIRRANRQPTNLSSSVLSTDSMHIGILAAAAHAAANNSPFTVFYNPRASPSEFVVPLAKYYKAVSANQISLGMRFRMMFETEESGTRRYMGTITGISDLDSVRWKGSQWRNLQVGWDESTGGERRNRVSIWEIEPVIAPFFICPPPFLRSKRPRQPGMPDDDSSDLDGICKRTMFGDDFCMKDPQGYPGLNLVQWMNMQNPSLSNAMQPSYMHSFSGSMLPNLGGVDISRQLGLSNTQIPQSNNIQFNAQRLLSQAQQLDQLPKLPSSMNSMGSVVQPSQQLDDMSQQTRSNLINQNAVSSQIQAQIMQQQQPHTNGIVLQQNQQLQRSLPQNQSQTMNQSPLPDPMIHQLQMSDNQVPMQMLQKLQQQQQQQQSLLAQQSVVLQPAQLPEQLRQSVDASFSRSMSSNQMLDIPQSTPAAVPPSNVSPQQAAKSNGTTNNRFSNQHMQPKLPQLHQPPPSTVLSDMSRPVGLPPTQTYNQLSAATSSVITGAAAAGQSGVTDDIPSCSTSPSTNNGPSLIQPVNNGRVHRSTGLVEDVAQSSATIFSTNTLTAVKPSLNISKNQSHGIFAQQTFLSGVVSQTDFLDTSSSTTSACLSQNEAQLQQNNVMSFNSQPMLFRDSTQDLEVPTDLHNIPYGTNVDGPLVAQLNSDPLLNKGIGELGKDFSNNFSSGAMLTTYDAQKDPQQEISSSIVSQSFGIPDMTFNSMDSAMNDNAFLNRNQWAPPPPFQRMRTYTKVYKRGAVGRSIDITSYSGYDELKQDLARRFGIEGQLEDRQKIGWKLVYVDHENDVLLVGDDPWEDFVNCVRSIKILSPQEVQQMSLDGDFGNGVLLNQACSSSDGGNA

>CpARF21

MGMKAPPNGFLPNSGEGERKNINSELWHACAGPLVSLPPVGSLVVYFPQGHSEQVAASMNKETDFIPSYPNLPSKLICMLHNVTLHADLETDEVYAQMTLQPVNKYEKEALLASDIGLKQNRQPAEFFCKTLTASDTSTHGGFSVPRRAAEKIFPPLDYSMQPPAQELVARDLHDNSWTFRHIYRDEKSQLLLGIRRANRQQPALSSSVISSDSMHIGILASAAHAAANNSPFTIFYNPRASPSEFVIPLAKYNKAMYAQVSLGMRFRMMFETEESGVRRYMGTITGISDMDSVRWKNSQWRNLQVGWDESAAGERPNRVSIWEVEPVVTPFYICPPPFFRPKFPKQPGMPDDESEIENAFKRAMPWFGDEFGMKDASSSIFPGLSLVQWMSMQHNNQFPAAQSGILPSMVAPSALHGTLTNDESSKLLSFQAPLIYHHCLCPISSSSILVEGAYKQKNITAMAFQLPPLPLSNQQQFNTSGGSLQTEKHNSNGFSSLGLMQSQQAPITHSHNQFKPTMAIRAYSGLTDGDAPSCSTSPSTNNCQVSASNMLNKNQQGAATLGGDPVAEPATNLAQDLQRKPDLRIKHEFPSSKGLDQLKYKGTVPDQLEACSSGTSYCLDAGTIQQTFPLPTCLDNDVQSHPRNNIPFSNSMDGLAPDTLLSRGYDSQKDLQNLLSNYGGGVPRDIETELSTAAISSQSFGVPNLPFKSDCSNDVNVNEAGALSSGLWANHSQRMRTYTKGNHRTFVVSRNEEEMRDLVIDPQRTDWKLVYVDHETDILLVGDDPWEEFVSCVQSIKILSSAEVQQMSLDGNLGHIQAPNQACSGTDSGNAWRGQYDDNSAASFNR

>CpARF22

MACNGGDSYTQSCVSTQGSGRNDLYTELWRACAGPLVDVPRVDERVFYFPQGHMEQLEASTNLELNKRIPLCNLSSKILCRVIHIELLADHESDEVYAQITLMPESNQNEPNSLDPCRPEPARPVVHSFCKVLTASDTSTHGQPRRHLLTTGWSTFVTSKRLSAGDSFVFLRGDNGEMHVGVRRRARQQSSMPSSVISSQSMHLGVLATASHAVTTQTRFVVYYKPRTCQFIISLNKYLEAVNNKFSVGMRFNMSFEGEDSPERRFSGTIIGATDISPHWPNSSWRSLRVQWDEQTSILRPDRVSPWDIEPLTSSAVTGLSQPISKNKRPRQSTPAHDGADLPKPALWDSGLAESHDGKQSSNAAESRKGENNELCHHRETDAISNRTCVSRTQADGTWLSSTQSNSHKHPVNDMAQDYKTVPGCGWSFLSGPSTSHLVKLSDEQILDSTENGRKGETVASCRLFGIDLNHLAKLPAEKASYQPSSVSSDTDGRSSTLSVAQSDPKSDNLEVSVERKSEPLQASLKETQSNQSSSANTRSRTKVHMHGMAVGRAVDLTILEGYDQLIDELEKMFDVRGQLCARDKWEIVYTDDEGDMMLVGDDPWEEFCNMVRRIFICSREQVKKMSSGSKQLTSIEVEGAAIIPDSPAV

>CpARF23

YWGKLDMKLSTSGFGQQDHEDDLYWGKLDMKLSTSGFGQQDHEGGEKKCLNSELWHACAGPLVSLPTAGTRVVYFPQGHSEQVAATTNKEVDGHIPNYPNLPPQLICQLHNVTMHADVETDEVYAQMTLQPLTAQEQKDTFLPMELGIPSRQPTNYFCKTLTASDTSTHGGFSVPRRAAEKVFPPLDFSQQPPAQELIARDLHDVEWKNEKNQLLLGIRRATRPQTVMPSSVLSSDSMHIGLLAAAAHAAATNSCFTVFYNPRASPSEFVIPLTKYVKAVFHTRVSVGMRFRMLFETEESSVRRYMGTITGISDLDPVRWPNSHWRSVKVGWDESAAGERQPRVSLWEIEPLTTFPMYPSLFPLRLKRPWHPGVSSVHDNRDDASNGLMWLRGGVGDQGLHSLNLQSMGSLPWLQQRLDSPMFGNDHNQQYQAMLAAGMPNLGGVDMLRQQIMHLQQPFQYIQQAGFHNSLLQQQQQLVQQSMPQNILQTPSQVMAENIPQHILQQTPQNQSEDIPNQQQRTYHDTLQVQSNQFHQGENSNVPSPPFPRTELMDSNTNYSESMISRRNILASSCAEGTGNLSNIYRSGQSILTEQLPQQSPVSKNAHPQVDAHSNSMTFPPPFSGRDSILELGNCNSDSPSRTLFGVNIDSSGLLLPSNVPTYASPSIGPDSSSMPMGDSGFQNSMYSCVQDSSELLHSSGQVDPLNPTRTFVKVYKSGSVGRSLDISRFSSYQELREELAQMFGIEGQLVDPHRSGWQLVFVDRENDVLLLGDDPWEAFVNNVWYIKILSPQDFQKLGVQAIESFNPVGGQRLTSGGNEAENVSGLPSVGRNSSI

>CpARF24

MTRGMDSVVEPMRNHEKYLDSQLWHACAGGLIELPTIDSKVVYFPQGHAEHAQENVDFGVAQIPSLIPCRVSGIRHMADSETDEVFANIRLIPLRNNEFSLDDDDELLGHNDIRTREKPASFAKTLTQSDANNGGGFSVPRYCAETMFPRLDYSAEPPVQTILAKDVHGEIWKFRHIYRGTPRRHLLTTGWSNFVNQKKLVAGDSIVFLRAETGDLFIGVRRAKRGNGYGIDYETGWDPTNSASPLVGYSDFMRENEGRLVRRISNGNSSGRVTVESVIEAAMLAASGKPFEIVYYPCTGTPEFVVKASCLRSAMQVHWYPAMRFKMPFETKDSSRISWFMGTISSIQVADPVRWPDSPWRMLQVAWDEPDLLQNVKSINPWLVEVVVNMPAIHVSPFSPPRKKQRFHPAETAVFGHLPMPSFSTNFFETTSSLQSVTGNNIPAGIQGARHTQFGLSSPDFQLSKLNLGPCSAGFKHLDEATPLPGSPGENMFGGMKSPDNSHWLTMGNNTESSKDSKETKPDHIILFGQLILPNQLISNSCSADTTNASYENQGKSSNPSDGSGLSSQQNGSLENSSEGGSTSYNGHEKTGFSLNTGQCKVFMEFEDIGRTLNLSLLRSYDELYGKLANMFGLGSSEMLNCVLYEDALGTTKQAGDEPFSTFLKTARKLTILTDSRSSDNSKT

>CpARF25

MANRGGGSFSSQGGGGDGLYTELWKASAGPLVEVPRVNDKVFYFPQGHMEQLEASTNHELNQKHPLFKLSSKILCRVVDVRLMAEQETDEVYAQITLMPDSNQEEPTNPDPSPPECRKPKVHSFCKVLTASDTSTHGGFSVLRKHATECLPPLDMTQQTPTQELVAKDLHSYEWRFKHIFRGQPRRHLLTTGWSTFVTSKRLVAGDSFVFLRGENGELRVGVRRLARQQTSMPSSVISSDSMHLGVLATASHAVSTLTRFVVYYKPRASQFIVSLNKYIEAMNRKFLVGMRFKMRFEGEESPERRFSGTIVGVDDISPHWPNSKWRSLRIQWDELASIPRPDRVSPWEIEPFVAPTSPSIPQSVSVKNKRLRPPLDIPDSDNSTVTTLRHPGSTQSHDDRTQLSVTAAEMKRFENHATWNYKQTDVSSIGNSISRTPKEGSWLASPNGSVSQHRLQNLTDDRNSNYVWSTVFSGAPAAQSTCPAPHPSNPKSSDEVNDLGEKGRKTEVAPSCRLFGIDIIGHSKSPVPPEMAADQPISAPNEITDAEQNSDQPKASKERKLGLLQVPPKEIQHKQSSSTNSRSRTKVRLYEIYYTDYSCLELYGHTLIYRLLVGCQYMKVQMQGMAVGRAVDLTMLEGYGQLIDELEKMFDIKGELHPRDKWEIVFTDDEGDTMLMGDYPWQEFCNMVRRIYIWSSQDVKMMSSVSKLTMSAMECDGTVITSESADS

>CpARF26

MGIQMKEAEKSLDPQLWHACAGGMVQMPAINSKVFYFPQGHAEHAQATVDFTSSLRIPPLILCRVLAVKFLADPETDEVFANVSLVPLANNDLNFEEEGGFGSNGSDNNMEKPASFAKTLTQSDANNGGGFSVPRYCAETIFPRLDYTADPPVQTVIAKDVHGEVWKFRHIYRGTPRRHLLTTGWSSFVNQKKLVAGDSIVFLRSKNGDLCVGIRRAKRAIGCAADHPYGWNPGGGNCFPPCGGLTMFLRDEDNNKLSRKGSVSSSGGGGNLRGKSKIRPESVIEAAALAASGQPFEVVYYPRASTPEFCVKASSVRAAMRIQWCSGMRFKMPFETEDSSRISWFMGTTSSVQVADPIRWPNSPWRLLQVTWDEPDLLQNVKRVSPWLVELVSNMPVIQLSPFSSPRKKFRLPQHPDVPLDSQFPLSSSSSSFSSNTLRPSSPMCCLSDNTSVGIQGARHTHFGISLSDFHLNNKLQLGLVPSSFQQLHFHSRISNTDHTRSSSALLNGEKTGPKLERSDSAKKKHQFVLFGQPILTEQQICCSSSSDIHQVSPNKSDTELGLDIGHCKVFMESEDVGRTLNLSVIGSYEELYRRLANMFGMEKPDILSHVLYQDATGAVKQAGDKPFSDFIKTARRLTILTDESGSDKMGRRTLMDGVLSGENGRLDASNKTGPLSILA

>CpARF27

MEIDLNQTASEEGKNAYCHGNCEEGRCNCCLSSSTSSCSSNSSSTPASSSTYLELWHACAGPLTSLPKKGNAVVYFPQGHLEQIASASPFSPMEMATFDLQPQILCRVINVHLLANKENDEVYTQLTLLPLPELLGTGVEGKELEELALNRAADGDGSGGSPTRSTPHMFCKTLTASDTSTHGGFSVPRRAAEDCFPPLDYTQLRPSQELIAKDLHGVEWRFKHIYRGILGENGELRLGIRRAVRPRNGLPDSIVGNQNSCANDLTRVVKAVSTKSTFDVFYNPRAYHAQFVVSCQKYVKSINNPVNVGTRFKMRFEMDDSPERRFNGVVVGIGDMDPFRWPNSKWRCLTVRWDKDSDHQERVSPWEIDPSVSLPPLSVQSSPRLKKLRTSLQAAPPNNAFNGRGGFMDFEDSVRSSKVLQGQENVGMVSPFYGCDNTAKRSLEFEVRSSAQQNQASGGVEKLNIGDYVTSFTGFMESDRFLKVLQGQEICSLRPQTRKPEPSLGVWGKFNLSDNSFNPFQSPNSSFYHMMSNGARNMHYPRRDIYSTGQAAMMSSNDINFPRESALFNPSAKMERANSTPPTLGSTMRNSKDENVHKQGSLVGRAIDLSRLNGYTDLLSELERLFSMEGLLKDPDKGWRVLYTDNENDVMVVGDYPWHDFCDAVSKIHIYTEEEVEKMTNGVISDDTQSCLDQAALCMEASKSSSVGQPDSSPTVVRV

>CpARF28

MTLSEQNPAEFRKGLEGEGLFEELWKACAGPLVEVPFNGERVFYFPQGHMEQLEESTNHELNHQIPHFDLPSKILCRVVNIRLLAEKETDEVYAQITLQPEADQSEPQCPDPEPPERTRPTVHSFCKILTASDTSTHGGFSVLKKHATECLPPLDMSQSTPTQELAAKDLHGHEWKFKHIFRGQPRRHLLTTGWSTFVVSKRLVAGDAFVFLRGDNGELRVGVRRQARQQSLMPSSVISSHSMHLGVLATASHAVRTQTFFVVYYKPRTSQFIIGLNKYLETIKNRYEVGMRFKMRFEGEESPERRFTGTIVGVGDISPIWSDSKWRSLKIQWDEAAAIQRPERVSPWEIEPFVPSASLNFTHPSIKSKRARPVEVPPPENTSSSTPTGFWLHGSTIPHEIPQLSGANDVPSSGNQVVWSLWQQTLDVNVDSSRSHCNPMAHVEGIWPSPPPPLCNNSLKPLPSPYSPSSTSKPSSELIEHDQSEKGNKPDISLGCRIFGIDLKKNSSIVPSLEKKSCCQTTVATDIAKDPVPIAAVTSQADAGKEQQQVASELSMKGTQTNHIPNSSSRTRTKVQMQGVAVGRAVDLTTLKGYEDLIDELENVFEIKGELREMNKWSIVFTDDEYDMMLVGDDPWPEFCQMVKRIFIYSSEEVKTMSSNSKLVSPPSLDSLDSERKTES

>CpARF29

MASSEVSINPNSASVPFNDHADSTKLTSDPPNALSARDADFALFTELWNACAGPLVSVPRENDRVFYFPQGHIEQVEASTSQVADQQMPVYDLPSKILCRVINVHLKAEPDTDEVFAQITLVPEANQDEHAVDKEPPPPPPRRFHVHSFCKTLTASDTSTHGGFSVLRRHADECLPPLDMSRQPPTQELVAKDLHGNEWRFRHIFRGQPRRHLLQSGWSVFVSSKRLVAGDAFIFLRGENGELRVGVRRAMRQHGNVPSSVISSHSMHLGVLATAWHAISTGTMFTVYYKPRTSPSEFIVPYDQYMESIKKSYTIGMRFKMRFEGEEAPEQRFTGTIIGCEDADPKRWKDSKWRCLKVRWDETSTISRPEKVSPWKIEPALAPPALNPLPMTRPKRPRSNMVPTSPDSSVLTREGSSRVTVDPSPVSAFTRVLQGQEFSTLRGNFIDGNDPDAAEKSVMWPPSLDDEKIDVVSTSKKHGADCWITPGRSEPTYADLLSGFGTNIDSSHGVRAGMGDPAVVTANSIRKHVMDQDGKFNFLGGSSWSVLPSSLSLNLVDSSQKGHIQAGDLSYQVRGNATFNGFGDHSVAHCHRTEQPHGNWLMAPLSSHFDYPVHSTELMSMPMVFQNQDIMKPKDGNCKLFGISLIKNPAIPDPAGLNRNMNGADVTHLNIHQIHSNESDLKSEPFRGSMLADKSLAINDADKLQQTCTQNLKDAHCKSQGTSARSCTKVQKQGIALGRSVDLSRFNNYDELVAELDQLFEFGGELLAPKKNWLIVYTDDEGDMMLVGDDPWQEFCGMVRKIFIYTREEVQKMNRGSLNLKGDENPSVEGKEAKEIEGQAVPSISAPESS

>CpARF30

MITFMDSKEKVKEMEKCLDPQLWHACAGGMVQMPPVNARVFYFPQGHAEHACAPVDFRNCSKVPPYTLCRVSAIKFLADPDTDEVFAKLRLIPINGSELEFEDDGIGRLNGSEQDKPTSFAKTLTQSDANNGGGFSVPRYCAETIFPRLDYSADPPVQTILAKDVHGETWKFRHIYRGTPRRHLLTTGWSTFVNHKKLVAGDSIVFLRAENGDLCVGIRRAKRGIGDGPEPPCGWNPAGGNCAVPYGAFSTFLREDENRVNRTNGKGKVKAESVIEAATLAANGQPFEIVYYPRASTPEFCVKAGLVKAALQIRWCSGMRFKMAFETEDSLRISWFMGTINTVQAADPLRWPESPWRLLQVTWDEPDLLQNVKRVSPWLVELVSNISPIHLAPFSPPRKKFRYPQHPDFPHDNQPSMSPFASYLHGPGSPFGCPPDNNPAGMQGARHAHFGLSLSDFHLSKLQSGLFPIRYRSLDPAAGSTRLSGNAMTEKPSMSENVSCLLTMAHSTQTSKKFDNVKTPQLILFGRPILTELQMSQSCSGDTVSPVGTGNSSSDGNGSGSALHQQGLPERSSCENFQWYKDNRQDVEPNLDTGHCKVFMESEDVGRTLDLSSLGSYEELYRKLGNMFGIDNSEMLNHVLYRDISGAVKHVGDEQFSEFMKTARRLTILTDSGSNNVILVLNVNGSGFKFKEVSRDTSRPLQCDV

>CpARF31

MAALIDLNTTEEDDAPSSAASSASSSSASALTSSPSPSLTSSICLELWHACAGPLTSLPNKGSLVVYFPQGHLEQMQEFPATAAYDLPPHILCRVIDVQLHAEAGSDEVYAQVSLFPENEPIEHKMQEEMTNDSEGEDFEGSEKTTTPHMFCKTLTASDTSTHGGFSVPRRAAEDCFPPLDYSQQRPSQELVAKDLLGIKWKFRHIYRGQPRRHLLTTGWSAFVNKKRLVSGDAVLFLRGNDGELRLGIRRAAQLKSGSPFSNICSQQLNSSSIMDVFNAISSKTSFSVYYNPRATSSQFVLPFHKFLKSINHSFSAGMRFSLSFETDDAADRRCTGRITGVGDVDPIRWPGSRWRSLVVRWDDVETKRHGRVSPWEIEPSGSVSVASNVVPPGLKRTRIGLSPTELEFPVPNGIGASDFGESLRFQKVLQGQEILGYSTPIDGDDNIRHPPEKRRSFPGLLGSGIALMRNGPRIPVTNSETSSRGFMFDESFQFHKVLQGQEMYPSPFFGIATATNEVKASGGYGPVDSVPLSRSKDGWPMTMQSENFLTRSSIPSVQVSSPSSVFMFQQSMVPVPSFNSYNRGNFTEQRTMNKSTSHNSGTAFMTDHNSSTEVPQGMRPTSLGVQKQLGLSHPSTTEPAFTGNKAGCRLFGFSLTEGKNVGNTTDKASPATTPINAGTTSVLSSNSGLRCPLKSPLMNKVVGSNCTKGAVQYHFANCSTYY

>CpARF32

MKSVIEEIDFGASNLGVNTSLGLLDLTEMRLSAAGFSPQPPEGEKRVLNSELWHACAGPLVSLPAVGSRAVYFPQGHSEQVAISTNKEVDAHIPSYPSLPPQLICQLHNVTMHADIETDEVYAQMTLQPLSAQEQKEPYLPAELGAPSKQPTNYFCKTLTASDTSTHGGFSVPRRAAEKVFPPLVILKMNEKNQLLLGIRRASRPQTMMPSSVLSSDSMHLGLLAAAAHAAATNSRFTIFYNPRASPSEFIIPLAKYVKAVYHTRVSVGMRFRMLFETEESSVRRYMGTITGISDLDPARWPNSHWRSVKVGWDESTAGERQPRVSLWEIEPLTTFPMYPSPFSLRLKRPWPTGLPSFHGLKEDDLGLNSQLMWLRGDALDRGIQPLNLHGIGVAPWMQPRLDASMVGLQPEIYQAMAAAALQEMRTVDPAKAQAASLLQFQQTQNLPNRPATFMSPQMLQQPQPHQTFIQNDHEIQHLPHSQAPTQPTVLRQEMKHQTFNNEQQQQQQQQQQPQQQVFDHQQISSSISTMTQFGSVSQSLQTIPSFCRQQSFSDSNGIHMTNPIISPLHSLLGSFPQDESSQLLNLPRTHPMIHSSTWPSKRAAIDPHISSGNSQFVHLQEENMGTAQANISQNVSLPPFPGRECSLDQRNGDPQSNLLFGVNIEPSSLLMQNGMPNIRGICSDSDSTAIPFSSNYVNTAGTNFSANPAGTPSNCIEDSGILPNFVKVYKSGTFGRSLDISKFSSYHQLRSELAHKFNLEGELEDPLRSGWQLVFVDRENDVLLLGDDPWQEFVNSVWCIKILSPQEVQEMGRVDSYADRQESSRNLSSGITSVGSLEY

>CpARF33

MRLSAAGFSPQPPEGERRVLNSELWHACAGPLVSLPAVGTRVVYFPQGHSEQVAASTNKEVDAQIPNYPSLPPQLICQLHNLTMHADAETDEVYAQMTLQPLSAQELKEAYLPAELGSPSRQPTNYFCKTLTASDTSTHGGFSVPRRAAEKVFPPLDFSMQPPAQELIARDLHDNEWKFRHIFRGQPKRHLLTTGWSVFVSAKRLVAGDAVLFIWNEKNQLLLGIRRASRPQTVMPSSVLSSDSMHLGLLAAAAHAAATNSRFTIFFNPRASPSEFVIPLTKYVKAVYHTRVSVGMRFRMLFETEESSVRRYMGTITGISDLDPVRWQNSHWRSVKVGWDESTAGERQPRVSLWEIEPLTTFPMYPSPFPLRLKRPWPTGLPSFGIKDSDLGMNSPFMWLRGDNCLNFQGNGVSPWMQPRLDTSMMAMQPDMYQAMATAALQEMRAIDYSKIAPASVLQFQQSQGLPCQSSSLMQPQMMHQSQPQQAFLQSIQENQQHHLQPQLQPQQFNNQQQQPRQPQPPQLDHQQIPCTIPAAISQFASCSQSQLPSLQTIPSLCQQPSFSDSNCNPVTTTVSPLHSLAGSSSVQDESSQLLNLQRANSIIPSAGWPAKRAAIDPLTTGASQYFLPQGEILGTSQSSIPQNTVALPPFPGRECSNGDRDDGSDPQNHVLFGVNIESSSLLMQNGASHLRGVGNDSASTTLPFSSNYMSTAGTDFSVNPTMTSSNCIDESGFLQSHENVGQVNHPPNGTFVKVHKSGTYSRSLDITKFNSYPELRSELACMFGLEGELEDPLRSGWQLVFVDRENDVLLLGDGPWPEFVSSVWCIKILSPEEVHEMGKRGLELLNSVPIQRLSNSTCDDYGSRQDSRNLVSGITSVGPLDY
