# Supplementary material for: Identification of ARF genes in Cucurbita pepo L and analysis of expression patterns, and functional analysis of CpARF22 under drought, salt stress
Source: BMC Genomics. 2024 Jan 25;25:112. doi: 10.1186/s12864-024-09992-8 (PMC10809590; doi:10.1186/s12864-024-09992-8)
Supplement: Supplementary file 2 — Additional file 2: Table S2. Amino acid sequence information of 101 ARF genes. [file 12864_2024_9992_MOESM2_ESM.docx]

Table S2 The 101 ARF gene-coding protein sequence information in this study

>AT1G19220.1

MKAPSNGFLPSSNEGEKKPINSQLWHACAGPLVSLPPVGSLVVYFPQGHSEQVAASMQKQ

TDFIPNYPNLPSKLICLLHSVTLHADTETDEVYAQMTLQPVNKYDREALLASDMGLKLNR

QPTEFFCKTLTASDTSTHGGFSVPRRAAEKIFPPLDFSMQPPAQEIVAKDLHDTTWTFRH

IYRGQPKRHLLTTGWSVFVSTKRLFAGDSVLFVRDEKSQLMLGIRRANRQTPTLSSSVIS

SDSMHIGILAAAAHANANSSPFTIFFNPRASPSEFVVPLAKYNKALYAQVSLGMRFRMMF

ETEDCGVRRYMGTVTGISDLDPVRWKGSQWRNLQVGWDESTAGDRPSRVSIWEIEPVITP

FYICPPPFFRPKYPRQPGMPDDELDMENAFKRAMPWMGEDFGMKDAQSSMFPGLSLVQWM

SMQQNNPLSGSATPQLPSALSSFNLPNNFASNDPSKLLNFQSPNLSSANSQFNKPNTVNH

ISQQMQAQPAMVKSQQQQQQQQQQHQHQQQQLQQQQQLQMSQQQVQQQGIYNNGTIAVAN

QVSCQSPNQPTGFSQSQLQQQSMLPTGAKMTHQNINSMGNKGLSQMTSFAQEMQFQQQLE

MHNSSQLLRNQQEQSSLHSLQQNLSQNPQQLQMQQQSSKPSPSQQLQLQLLQKLQQQQQQ

QSIPPVSSSLQPQLSALQQTQSHQLQQLLSSQNQQPLAHGNNSFPASTFMQPPQIQVSPQ

QQGQMSNKNLVAAGRSHSGHTDGEAPSCSTSPSANNTGHDNVSPTNFLSRNQQQGQAASV

SASDSVFERASNPVQELYTKTESRISQGMMNMKSAGEHFRFKSAVTDQIDVSTAGTTYCP

DVVGPVQQQQTFPLPSFGFDGDCQSHHPRNNLAFPGNLEAVTSDPLYSQKDFQNLVPNYG

NTPRDIETELSSAAISSQSFGIPSIPFKPGCSNEVGGINDSGIMNGGGLWPNQTQRMRTY

TKVQKRGSVGRSIDVTRYSGYDELRHDLARMFGIEGQLEDPLTSDWKLVYTDHENDILLV

GDDPWEEFVNCVQNIKILSSVEVQQMSLDGDLAAIPTTNQACSETDSGNAWKVHYEDTSA

AASFNR

>AT1G19850.1

MMASLSCVEDKMKTSCLVNGGGTITTTTSQSTLLEEMKLLKDQSGTRKPVINSELWHACA

GPLVCLPQVGSLVYYFSQGHSEQVAVSTRRSATTQVPNYPNLPSQLMCQVHNVTLHADKD

SDEIYAQMSLQPVHSERDVFPVPDFGMLRGSKHPTEFFCKTLTASDTSTHGGFSVPRRAA

EKLFPPLDYSAQPPTQELVVRDLHENTWTFRHIYRGQPKRHLLTTGWSLFVGSKRLRAGD

SVLFIRDEKSQLMVGVRRANRQQTALPSSVLSADSMHIGVLAAAAHATANRTPFLIFYNP

RACPAEFVIPLAKYRKAICGSQLSVGMRFGMMFETEDSGKRRYMGTIVGISDLDPLRWPG

SKWRNLQVEWDEPGCNDKPTRVSPWDIETPESLFIFPSLTSGLKRQLHPSYFAGETEWGS

LIKRPLIRVPDSANGIMPYASFPSMASEQLMKMMMRPHNNQNVPSFMSEMQQNIVMGNGG

LLGDMKMQQPLMMNQKSEMVQPQNKLTVNPSASNTSGQEQNLSQSMSAPAKPENSTLSGC

SSGRVQHGLEQSMEQASQVTTSTVCNEEKVNQLLQKPGASSPVQADQCLDITHQIYQPQS

DPINGFSFLETDELTSQVSSFQSLAGSYKQPFILSSQDSSAVVLPDSTNSPLFHDVWDTQ

LNGLKFDQFSPLMQQDLYASQNICMSNSTTSNILDPPLSNTVLDDFCAIKDTDFQNHPSG

CLVGNNNTSFAQDVQSQITSASFADSQAFSRQDFPDNSGGTGTSSSNVDFDDCSLRQNSK

GSSWQKIATPRVRTYTKVQKTGSVGRSIDVTSFKDYEELKSAIECMFGLEGLLTHPQSSG

WKLVYVDYESDVLLVGDDPWEEFVGCVRCIRILSPTEVQQMSEEGMKLLNSAGINDLKTS

VS

>AT1G30330.1

MRLSSAGFNPQPHEGEKRVLNSELWHACAGPLVSLPPVGSRVVYFPQGHSEQVAASTNKE

VDAHIPNYPSLHPQLICQLHNVTMHADVETDEVYAQMTLQPLNAQEQKDPYLPAELGVPS

RQPTNYFCKTLTASDTSTHGGFSVPRRAAEKVFPPLDYSQQPPAQELMARDLHDNEWKFR

HIFRGQPKRHLLTTGWSVFVSAKRLVAGDSVLFIWNDKNQLLLGIRRANRPQTVMPSSVL

SSDSMHLGLLAAAAHAAATNSRFTIFYNPRASPSEFVIPLAKYVKAVYHTRVSVGMRFRM

LFETEESSVRRYMGTITGICDLDPTRWANSHWRSVKVGWDESTAGERQPRVSLWEIEPLT

TFPMYPSPFPLRLKRPWPPGLPSFHGLKEDDMGMSMSSPLMWDRGLQSLNFQGMGVNPWM

QPRLDTSGLLGMQNDVYQAMAAAALQDMRGIDPAKAAASLLQFQNSPGFSMQSPSLVQPQ

MLQQQLSQQQQQLSQQQQQQQQLSQQQQQQLSQQQQQQLSQQQQQQLSQQQQQQAYLGVP

ETHQPQSQAQSQSNNHLSQQQQQVVDNHNPSASSAAVVSAMSQFGSASQPNTSPLQSMTS

LCHQQSFSDTNGGNNPISPLHTLLSNFSQDESSQLLHLTRTNSAMTSSGWPSKRPAVDSS

FQHSGAGNNNTQSVLEQLGQSHTSNVPPNAVSLPPFPGGRECSIEQEGSASDPHSHLLFG

VNIDSSSLLMPNGMSNLRSIGIEGGDSTTLPFTSSNFNNDFSGNLAMTTPSSCIDESGFL

QSSENLGSENPQSNTFVKVYKSGSFGRSLDISKFSSYHELRSELARMFGLEGQLEDPVRS

GWQLVFVDRENDVLLLGDDPWPEFVSSVWCIKILSPQEVQQMGKRGLELLNSAPSSNNVD

KLPSNGNCDDFGNRSDPRNLGNGIASVGGSFNY

>AT1G30330.2

MRLSSAGFNPQPHEVTGEKRVLNSELWHACAGPLVSLPPVGSRVVYFPQGHSEQVAASTN

KEVDAHIPNYPSLHPQLICQLHNVTMHADVETDEVYAQMTLQPLNAQEQKDPYLPAELGV

PSRQPTNYFCKTLTASDTSTHGGFSVPRRAAEKVFPPLDYSQQPPAQELMARDLHDNEWK

FRHIFRGQPKRHLLTTGWSVFVSAKRLVAGDSVLFIWNDKNQLLLGIRRANRPQTVMPSS

VLSSDSMHLGLLAAAAHAAATNSRFTIFYNPRASPSEFVIPLAKYVKAVYHTRVSVGMRF

RMLFETEESSVRRYMGTITGICDLDPTRWANSHWRSVKVGWDESTAGERQPRVSLWEIEP

LTTFPMYPSPFPLRLKRPWPPGLPSFHGLKEDDMGMSMSSPLMWDRGLQSLNFQGMGVNP

WMQPRLDTSGLLGMQNDVYQAMAAAALQDMRGIDPAKAAASLLQFQNSPGFSMQSPSLVQ

PQMLQQQLSQQQQQLSQQQQQQQQLSQQQQQQLSQQQQQQLSQQQQQQLSQQQQQQAYLG

VPETHQPQSQAQSQSNNHLSQQQQQVVDNHNPSASSAAVVSAMSQFGSASQPNTSPLQSM

TSLCHQQSFSDTNGGNNPISPLHTLLSNFSQDESSQLLHLTRTNSAMTSSGWPSKRPAVD

SSFQHSGAGNNNTQSVLEQLGQSHTSNVPPNAVSLPPFPGGRECSIEQEGSASDPHSHLL

FGVNIDSSSLLMPNGMSNLRSIGIEGGDSTTLPFTSSNFNNDFSGNLAMTTPSSCIDESG

FLQSSENLGSENPQSNTFVKVYKSGSFGRSLDISKFSSYHELRSELARMFGLEGQLEDPV

RSGWQLVFVDRENDVLLLGDDPWPEFVSSVWCIKILSPQEVQQMGKRGLELLNSAPSSNN

VDKLPSNGNCDDFGNRSDPRNLGNGIASVGGSFNY

>AT1G34170.1

MENNGEMNAQPELSVDITKTYMYEKLWNICAGPLCVLPKPGEKVYYFPQGHIELIENSTR

DELDHIRPIFDLPSKLRCRVVAIDRKVDKNTDEVYAQISLMPDTTEVMTHNTTMDTRRPI

VYFFSKILTASDVSLSGGLIIPKQYAIECFPPLDMSQPISTQNLVAKDLYGQEWSFKHVF

RGTPQRHMFTSGGGWSVFATTKRLIVGDIFVLLRGENGELRFGIRRAKHQQGHIPSSVIS

ANCMQHGVIASVVNAFKTKCMFNVVYKPSSSQFVISYDKFVDAMNNNYIVGSRFRMQFEG

KDFSEKRYDGTIIGVNDMSPHWKDSEWRSLKVQWDELSPFLRPNQVSPWDIEHLIPSSDI

SQSSLKKKKHWLQLNEIGATLSNLWTCQEIGQRSMNSPISVPEFSYPNAIEDSKFLSGLL

LNHSLLAIPNENYNSDQMIQPRKEDITTEATTSCLLFGVDLTKVSKSKDSICPIESCKKS

EISKLKNQKATTSCLKIKSLTKPNL

>AT1G34170.2

MENNGEMNAQPELSVDITKTYMYEKLWNICAGPLCVLPKPGEKVYYFPQGHIELIENSTR

DELDHIRPIFDLPSKLRCRVVAIDRKVDKNTDEVYAQISLMPDTTEVMTHNTTMDTRRPI

VYFFSKILTASDVSLSGGLIIPKQYAIECFPPLDMSQPISTQNLVAKDLYGQEWSFKHVF

RGTPQRHMFTSGGGWSVFATTKRLIVGDIFVLLRGENGELRFGIRRAKHQQGHIPSSVIS

ANCMQHGVIASVVNAFKTKCMFNVVYKPRMQFEGKDFSEKRYDGTIIGVNDMSPHWKDSE

WRSLKVQWDELSPFLRPNQVSPWDIEHLIPSSDISQSSLKKKKHWLQLNEIGATLSNLWT

CQEIGQRSMNSPISVPEFSYPNAIEDSKFLSGLLLNHSLLAIPNENYNSDQMIQPRKEDI

TTEATTSCLLFGVDLTKVSKSKDSICPIESCKKSEISKLKNQKATTSCLKIKSLTKPNL

>AT1G34170.3

MENNGEMNAQPELSVDITKTYMYEKLWNICAGPLCVLPKPGEKVYYFPQGHIELIENSTR

DELDHIRPIFDLPSKLRCRVVAIDRKVDKNTDEVYAQISLMPDTTEVMTHNTTMDTRRPI

VYFFSKILTASDVSLSGGLIIPKQYAIECFPPLDMSQPISTQNLVAKDLYGQEWSFKHVF

RGTPQRHMFTSGGGWSVFATTKRLIVGDIFVLLRGENGELRFGIRRAKHQQGHIPSSVIS

ANCMQHGVIASVVNAFKTKCMFNVVYKPRMQFEGKDFSEKRYDGTIIGVNDMSPHWKDSE

WRSLKVQWDELSPFLRPNQVSPWDIEHLIPSSDISQSSLKKKKHWLQLNEIGATLSNLWT

CQEIGQRSMNSPISVPEFSYPNAIEDSKFLSGLLLNHSLLAIPNENYNSDQMIQPRKEDI

TTEATTSCLLFGVDLTKVHMQGVAISRAVDLTAMHGYNQLIQKLEELFDLKDELRTRNQW

EIVFTNNEGAEMLVGDDPWPEFCNMAKRIFICSKEEIKKMKLKNKFFQPESKALTSSDVP

PNVTDN

>AT1G34310.1

MESGNVVNAQPELSGIIDGSKSYVYEQLWKLCAGPLCDIPKLGEKVYYFPQGHIELVETS

TREELNELQPICDLPSKLQCRVIAIHLKVENNSDETYAEITLMPDTTQVVIPTQNENQFR

PLVNSFTKVLTASDTSAHGGFFVPKKHAIECLPSLDMSQPLPAQELLAIDLHGNQWRFNH

NYRGTPQRHLLTTGWNAFTTSKKLVAGDVIVFVRGETGELRVGIRRARHQQGNIPSSIVS

IDCMRHGVVASAKHAFDNQCMFTVVYKPRSSKFIVSYDKFLDAVNNKFNVGSRFTMRLEG

DDFSERRCFGTIIGVSDFSPHWKCSEWRSLEVQWDEFTSFPGPKKVSPWDIEHLMPAINV

PRSFLLKNKRLREVNEIGSSSSHLLPPILTQGQENEQLSVASPMNISLRYRDATEDAMNP

SKLLMSYPVQPMPKLNYNNQMVTEMEENITTKTGTNFRLFGVTLDTPPVIKDPIEEIGSE

ISKLTEGKKFGLSQTLRSPTEIQNKQFSSSRTCTKVQMQGVTIGRAVDLSVLNGYDQLIL

ELEKLFDIKGQLQTRNQWEIAFTDSDEDKMLVGDDPWPEFCNMVKKIFIQKRR

>AT1G34390.1

MESGNIVNAQPELSGIIDGSKSYMYEQLWKLCAGPLCDIPKLGEKIYYFPQGNIELVEAS

TREELNELKPICDLPSKLQCRVIAIQLKVENNSDETYAEITLMPDTTQVVIPTQNENQFR

PLVNSFTKVLTASDTSGGFFVPKKHAIECLPPLDMSQPLPTQELLATDLHGNQWRFNHNY

RGTPQRHLLTTGWNAFTTSKKLVAGDVIVFVRGETGELRVGIRRAGHQQGNIPSSIISIE

SMRHGVIASAKHAFDNQCMFIVVYKPRSSQFIVSYDKFLDAVNNKFNVGSRFTMRFEGDD

FSERRYFGTIIGVSDFSPHWKCSEWRNLEVQWDEFASFSRPNKVSPWEIEHLMPALNVPR

PSLLKNKRLREVNEIGSSSSHLLPPILTQGQEIGQLSVASPMNISLTYRDTTEDVMNPSR

LLMSYPVQPMPKLNYNNQMVTQIEENITTKTGTNFRLFGVSLVTPSVIKDPIEEIGSEIS

KLTEGKKFGQSQTLRSPTEIQSKQFSSTRTCTKVQMQGVTIERAVDLSVLNGYDQLILEL

EELFDLKGQLQTRNQWEIAFTDSDDDKMLVGDDPWPEFCNMVKKILIFKRGGQKLEVQ

>AT1G34410.1

MESGNIVNAQPKLSGIIDGSKSYMYEQLWKLCAGPLCDIPKLGENVYYFPQGNIELVQAS

TREELNELQPICDLPSKLQCRVIAIHLKVENNSDEIYAEITLMPDTTQVVIPTQSENRFR

PLVNSFTKVLTASDTSAYGGFSVPKKHAIECLPPLDMSQPLPAQEILAIDLHDNQWRFRH

NYRGTPQRHSLTTGWNEFITSKKLVKGDVIVFVRGETGELRVGIRRARHQQGNIPSSIVS

IDCMRHGVIASAKHAFDNQCIFIVVYKPRSSQFIVSYDKFLDAVNNKFNVGSRFTMRFEG

DDFSERRYFGTIIGVSDFSPHWKCSEWRSLEVQWDEFASFSRPNKVSPWEIEHLVPALNV

PRSSLLKNKRLREVNEFGSSSSHLLPPILTQGQEIGQLSVASPMNISLRYRDTTEAAMNP

SRLLMSYPVQPMPKLNYNNQMVTQIEENITTKAGTNFRLFGVTLDTPPMIKDPIKQIGSD

ISKLTERKKFGQSQTLRSPIEIQSKQFSSSRTCTKVQMQGVTIGRAVDLSVLNGYDQLIL

ELEKLFDIKGQLQTRNQWKIAFTDSDGYEMLVGDDPWPEFCKMVKKILIYSKEEVKNLKS

SKSLSS

>AT1G35240.1

METGNVVNAQPELSGIIDGSKSYMYEQLWKLCAGPLCDIPKLGENVYYFPQGNIELVDAS

TREELNELQPICDLPSKLQCRVIAIHLKVENNSDETYAEITLMPDTTQVVIPTQSENQFR

PLVNSFTKVLTASDTSAYGGFFVPKKHAIECLPPLPLPAQELLAKDLHGNQWRFRHSYRG

TPQRHSLTTGWNEFTTSKKLVKGDVIVFVRGETGELRVGIRRARHQQGNIPSSIVSIDCM

RHGVIASAKHALDNQCIFIVVYKPSIRSSQFIVSYDKFLDAMNNKFIVGSRFTMRFEGDD

FSERRYFGTIIGVNDFSPHWKCSEWRSLEVQWDEFASFSRPNKVSPWEIEHLMSALNVPR

SSLLKNKRLREVNEFGQEIGQLSVASPMNTSLRYRDTTEDAMNPSRLLMSYPVQPMPKLN

YNNQMVTQIEENITTKAVTNFRLFGVSLAIPLVIKDPIEEIGSDISKLTEGKKFGQSQTL

RSPIEIQSKQFGSTRTCTKVQMQGVTIGRAVDLSVLNGYDQLILELEKLFDLKGQLQTRN

QWKIAFTDSDGYEMLVGDDPWPEFCKMVKKILIYSKEEVKNLKSSKSLSS

>AT1G35520.1

METGNVVNAQPELSGIIDRSKSYMYEQLWKLCAGPLCDIPKLGEKVYYFPQGNIELVEAS

TREELNELQPICDLPSKLQCRVIAIHLKVENNSDETYAKITLMPDTTVSENLQVVIPTQN

ENQFRPLVNSFTKVLTASDISANGVFSVPKKHAIECLPPLDMSQPLPAQELLAIDLHGNQ

WSFRHSYRGTPQRHLLTTGWNEFTTSKKLVKGDVIVFVRGETGELRVGIRRARHQQGNIP

SSIVSIDCMRHGVIASAKHAFDNQCMFIVVYKPRSSQFIVSYDKFLDAVNNKFNVGSRFT

MRFEGDDLSERRYFGTIIGVSNFSPHWKCSDWRSLEVQWDEFASFLRPNKVSPWEIEHLM

PALNVPRSSFLKNKRLREVNEFGSSSSHLLPPILTQGQEIGQLSVASPMNISLLYRETTE

DAMNPSRLLMSYPVQPMPKRNYNNQMVTQIEENITTKAGTNFRLFGVSLATPPVIKDPIE

QIGSDISKLTEGKKFGQSQTLRSPTKIQSKQFSSTRTCTKVQMQGVTIGRAVDLSVLNGY

DQLILELEKLFDLKGQLQTRNQWKIIFTGSDEDEMLVGDDPWPEFCNMVKRIYIQKRR

>AT1G35540.1

MESGNVVNTQPELSGIIDGSKSYMYEQLWKLCAGPLCDIPKLGEKVYYFPQGHIELVEAS

TREELNELQPICDFPSKLQCRVIAIQLKVENNSDETYAEITLMPDTTQVVIPTQNQNQFR

PLVNSFTKVLTASDTSVHGGFSVPKKHAIECLPPLDMSQPLPTQEILAIDLHGNQWRFRH

IYRGTAQRHLLTIGWNAFTTSKKLVEGDVIVFVRGETGELRVGIRRAGHQQGNIPSSIVS

IESMRHGIIASAKHAFDNQCMFIVVYKPRSSQFIVSYDKFLDVVNNKFNVGSRFTMRFEG

DDFSERRSFGTIIGVSDFSPHWKCSEWRSLEVQWDEFASFPRPNQVSPWDIEHLTPWSNV

SRSSFLKNKRSREVNEIGSSSSHLLPPTLTQGQEIGQQSMATPMNISLRYRDITEDAMTP

SRLLMSYPVQPMAKLNYNNVVTPIEENITTNAVASFRLFGVSLATPSVIKDPVEQIGLEI

SRLTQEKKFGQSQILRSPTEIQSKQFSSTRTCTKVQMQGVTIGRAVDLSVLNGYDQLILE

LEKLFDLKGQLQARNQWEIAFTNNEEDKMLVGEDPWPEFCNMVKKIFIYSKEEVKNLKSR

KSLSS

>AT1G59750.1

MAASNHSSGKPGGVLSDALCRELWHACAGPLVTLPREGERVYYFPEGHMEQLEASMHQGL

EQQMPSFNLPSKILCKVINIQRRAEPETDEVYAQITLLPELDQSEPTSPDAPVQEPEKCT

VHSFCKTLTASDTSTHGGFSVLRRHADDCLPPLDMSQQPPWQELVATDLHNSEWHFRHIF

RGQPRRHLLTTGWSVFVSSKKLVAGDAFIFLRGENEELRVGVRRHMRQQTNIPSSVISSH

SMHIGVLATAAHAITTGTIFSVFYKPRTSRSEFIVSVNRYLEAKTQKLSVGMRFKMRFEG

EEAPEKRFSGTIVGVQENKSSVWHDSEWRSLKVQWDEPSSVFRPERVSPWELEPLVANST

PSSQPQPPQRNKRPRPPGLPSPATGPSGPVTPDGVWKSPADTPSSVPLFSPPAKAATFGH

GGNKSFGVSIGSAFWPTNADSAAESFASAFNNESTEKKQTNGNVCRLFGFELVENVNVDE

CFSAASVSGAVAVDQPVPSNEFDSGQQSEPLNINQSDIPSGSGDPEKSSLRSPQESQSRQ

IRSCTKVHMQGSAVGRAIDLTRSECYEDLFKKLEEMFDIKGELLESTKKWQVVYTDDEDD

MMMVGDDPWNEFCGMVRKIFIYTPEEVKKLSPKNKLAVNARMQLKADAEENGNTEGRSSS

MAGSR

>AT1G59750.2

MAASNHSSGKPGGVLSDALCRELWHACAGPLVTLPREGERVYYFPEGHMEQLEASMHQGL

EQQMPSFNLPSKILCKVINIQRRAEPETDEVYAQITLLPELDQSEPTSPDAPVQEPEKCT

VHSFCKTLTASDTSTHGGFSVLRRHADDCLPPLDMSQQPPWQELVATDLHNSEWHFRHIF

RGQPRRHLLTTGWSVFVSSKKLVAGDAFIFLRGENEELRVGVRRHMRQQTNIPSSVISSH

SMHIGVLATAAHAITTGTIFSVFYKPRTSRSEFIVSVNRYLEAKTQKLSVGMRFKMRFEG

EEAPEKRFSGTIVGVQENKSSVWHDSEWRSLKVQWDEPSSVFRPERVSPWELEPLVANST

PSSQPQPPQRNKRPRPPGLPSPATGPSGPDGVWKSPADTPSSVPLFSPPAKAATFGHGGN

KSFGVSIGSAFWPTNADSAAESFASAFNNESTEKKQTNGNVCRLFGFELVENVNVDECFS

AASVSGAVAVDQPVPSNEFDSGQQSEPLNINQSDIPSGSGDPEKSSLRSPQESQSRQIRS

CTKVHMQGSAVGRAIDLTRSECYEDLFKKLEEMFDIKGELLESTKKWQVVYTDDEDDMMM

VGDDPWNEFCGMVRKIFIYTPEEVKKLSPKNKLAVNARMQLKADAEENGNTEGRSSSMAG

SR

>AT1G59750.4

MAASNHSSGKPGGVLSDALCRELWHACAGPLVTLPREGERVYYFPEGHMEQLEASMHQGL

EQQMPSFNLPSKILCKVINIQRRAEPETDEVYAQITLLPELDQSEPTSPDAPVQEPEKCT

VHSFCKTLTASDTSTHGGFSVLRRHADDCLPPLDMSQQPPWQELVATDLHNSEWHFRHIF

RGQPRRHLLTTGWSVFVSSKKLVAGDAFIFLRGENEELRVGVRRHMRQQTNIPSSVISSH

SMHIGVLATAAHAITTGTIFSVFYKPRTSRSEFIVSVNRYLEAKTQKLSVGMRFKMRFEG

EEAPEKRFSGTIVGVQENKSSVWHDSEWRSLKVQWDEPSSVFRPERVSPWELEPLVANST

PSSQPQPPQRNKRPRPPGLPSPATGPSDGVWKSPADTPSSVPLFSPPAKAATFGHGGNKS

FGVSIGSAFWPTNADSAAESFASAFNNESTEKKQTNGNVCRLFGFELVENVNVDECFSAA

SVSGAVAVDQPVPSNEFDSGQQSEPLNINQSDIPSGSGDPEKSSLRSPQESQSRQIRSCT

KVHMQGSAVGRAIDLTRSECYEDLFKKLEEMFDIKGELLESTKKWQVVYTDDEDDMMMVG

DDPWNEFCGMVRKIFIYTPEEVKKLSPKNKLAVNARMQLKADAEENGNTEGRSSSMAGSR

>AT1G77850.1

MSPPSATAGDINHREVDPTIWRACAGASVQIPVLHSRVYYFPQGHVEHCCPLLSTLPSST

SPVPCIITSIQLLADPVTDEVFAHLILQPMTQQQFTPTNYSRFGRFDGDVDDNNKVTTFA

KILTPSDANNGGGFSVPRFCADSVFPLLNFQIDPPVQKLYVTDIHGAVWDFRHIYRGTPR

RHLLTTGWSKFVNSKKLIAGDSVVFMRKSADEMFIGVRRTPISSSDGGSSYYGGDEYNGY

YSQSSVAKEDDGSPKKTFRRSGNGKLTAEAVTDAINRASQGLPFEVVFYPAAGWSEFVVR

AEDVESSMSMYWTPGTRVKMAMETEDSSRITWFQGIVSSTYQETGPWRGSPWKQLQITWD

EPEILQNVKRVNPWQVEIAAHATQLHTPFPPAKRLKYPQPGGGFLSGDDGEILYPQSGLS

SAAAPDPSPSMFSYSTFPAGMQGARQYDFGSFNPTGFIGGNPPQLFTNNFLSPLPDLGKV

STEMMNFGSPPSDNLSPNSNTTNLSSGNDLVGNRGPLSKKVNSIQLFGKIITVEEHSESG

PAESGLCEEDGSKESSDNETQLSLSHAPPSVPKHSNSNAGSSSQG

>AT2G28350.1

MEQEKSLDPQLWHACAGSMVQIPSLNSTVFYFAQGHTEHAHAPPDFHAPRVPPLILCRVV

SVKFLADAETDEVFAKITLLPLPGNDLDLENDAVLGLTPPSSDGNGNGKEKPASFAKTLT

QSDANNGGGFSVPRYCAETIFPRLDYSAEPPVQTVIAKDIHGETWKFRHIYRGTPRRHLL

TTGWSTFVNQKKLIAGDSIVFLRSESGDLCVGIRRAKRGGLGSNAGSDNPYPGFSGFLRD

DESTTTTSKLMMMKRNGNNDGNAAATGRVRVEAVAEAVARAACGQAFEVVYYPRASTPEF

CVKAADVRSAMRIRWCSGMRFKMAFETEDSSRISWFMGTVSAVQVADPIRWPNSPWRLLQ

VAWDEPDLLQNVKRVSPWLVELVSNMPTIHLSPFSPRKKIRIPQPFEFPFHGTKFPIFSP

GFANNGGGESMCYLSNDNNNAPAGIQGARQAQQLFGSPSPSLLSDLNLSSYTGNNKLHSP

AMFLSSFNPRHHHYQARDSENSNNISCSLTMGNPAMVQDKKKSVGSVKTHQFVLFGQPIL

TEQQVMNRKRFLEEEAEAEEEKGLVARGLTWNYSLQGLETGHCKVFMESEDVGRTLDLSV

IGSYQELYRKLAEMFHIEERSDLLTHVVYRDANGVIKRIGDEPFSDFMKATKRLTIKMDI

GGDNVRKTWITGIRTGENGIDASTKTGPLSIFA

>AT2G33860.1

MGGLIDLNVMETEEDETQTQTPSSASGSVSPTSSSSASVSVVSSNSAGGGVCLELWHACA

GPLISLPKRGSLVLYFPQGHLEQAPDFSAAIYGLPPHVFCRILDVKLHAETTTDEVYAQV

SLLPESEDIERKVREGIIDVDGGEEDYEVLKRSNTPHMFCKTLTASDTSTHGGFSVPRRA

AEDCFPPLDYSQPRPSQELLARDLHGLEWRFRHIYRGQPRRHLLTTGWSAFVNKKKLVSG

DAVLFLRGDDGKLRLGVRRASQIEGTAALSAQYNQNMNHNNFSEVAHAISTHSVFSISYN

PKASWSNFIIPAPKFLKVVDYPFCIGMRFKARVESEDASERRSPGIISGISDLDPIRWPG

SKWRCLLVRWDDIVANGHQQRVSPWEIEPSGSISNSGSFVTTGPKRSRIGFSSGKPDIPV

SEGIRATDFEESLRFQRVLQGQEIFPGFINTCSDGGAGARRGRFKGTEFGDSYGFHKVLQ

GQETVPAYSITDHRQQHGLSQRNIWCGPFQNFSTRILPPSVSSSPSSVLLTNSNSPNGRL

EDHHGGSGRCRLFGFPLTDETTAVASATAVPCVEGNSMKGASAVQSNHHHSQGRDIYAMR

DMLLDIAL

>AT2G46530.1

MANVEADFRTSGSNDDELYTELWKACAGPLVEVPRYGERVFYFPQGHMEQLVASTNQGVV

DQEIPVFNLPPKILCRVLSVTLKAEHETDEVYAQITLQPEEDQSEPTSLDPPLVEPAKPT

VDSFVKILTASDTSTHGGFSVLRKHATECLPSLDMTQPTPTQELVARDLHGYEWRFKHIF

RGQPRRHLLTTGWSTFVTSKRLVAGDAFVFLRGETGDLRVGVRRLAKQQSTMPASVISSQ

SMRLGVLATASHAVTTTTIFVVFYKPRISQFIISVNKYMMAMKNGFSLGMRYRMRFEGEE

SPERIFTGTIIGSGDLSSQWPASKWRSLQIQWDEPSSIQRPNKVSPWEIEPFSPSALTPT

PTQQQSKSKRSRPISEITGSPVASSFLSSFSQSHESNPSVKLLFQDPATERNSNKSVFSS

GLQCKITEAPVTSSCRLFGFDLTSKPASATIPHDKQLISVDSNISDSTTKCQDPNSSNSP

KEQKQQTSTRSRIKVQMQGTAVGRAVDLTLLRSYDELIKELEKMFEIEGELSPKDKWAIV

FTDDEGDRMLVGDDPWNEFCKMAKKLFIYPSDEVKKMRSKSLLGDKGTIVNLESDQRTVH

V

>AT2G46530.2

MRPMRFTLRSHYNQKKIEPTSLDPPLVEPAKPTVDSFVKILTASDTSTHGGFSVLRKHAT

ECLPSLDMTQPTPTQELVARDLHGYEWRFKHIFRGQPRRHLLTTGWSTFVTSKRLVAGDA

FVFLRGETGDLRVGVRRLAKQQSTMPASVISSQSMRLGVLATASHAVTTTTIFVVFYKPR

ISQFIISVNKYMMAMKNGFSLGMRYRMRFEGEESPERIFTGTIIGSGDLSSQWPASKWRS

LQIQWDEPSSIQRPNKVSPWEIEPFSPSALTPTPTQQQSKSKRSRPISEITGSPVASSFL

SSFSQSHESNPSVKLLFQDPATERNSNKSVFSSGLQCKITEAPVTSSCRLFGFDLTSKPA

SATIPHDKQLISVDSNISDSTTKCQDPNSSNSPKEQKQQTSTRSRIKVQMQGTAVGRAVD

LTLLRSYDELIKELEKMFEIEGELSPKDKWAIVFTDDEGDRMLVGDDPWNEFCKMAKKLF

IYPSDEVKKMRSKSLLGDKGTIVNLESDQRTVHV

>AT2G46530.3

MSQTSLEPLIISIIKLQILQLWLKLIAVGWNLGSNDDELYTELWKACAGPLVEVPRYGER

VFYFPQGHMEQLVASTNQGVVDQEIPVFNLPPKILCRVLSVTLKAEHETDEVYAQITLQP

EEDQSEPTSLDPPLVEPAKPTVDSFVKILTASDTSTHGGFSVLRKHATECLPSLDMTQPT

PTQELVARDLHGYEWRFKHIFRGQPRRHLLTTGWSTFVTSKRLVAGDAFVFLRGETGDLR

VGVRRLAKQQSTMPASVISSQSMRLGVLATASHAVTTTTIFVVFYKPRISQFIISVNKYM

MAMKNGFSLGMRYRMRFEGEESPERIFTGTIIGSGDLSSQWPASKWRSLQIQWDEPSSIQ

RPNKVSPWEIEPFSPSALTPTPTQQQSKSKRSRPISEITGSPVASSFLSSFSQSHESNPS

VKLLFQDPATERNSNKSVFSSGLQCKITEAPVTSSCRLFGFDLTSKPASATIPHDKQLIS

VDSNISDSTTKCQDPNSSNSPKEQKQQTSTRSRIKVQMQGTAVGRAVDLTLLRSYDELIK

ELEKMFEIEGELSPKDKWAIVFTDDEGDRMLVGDDPWNEFCKMAKKLFIYPSDEVKKMRS

KSLLGDKGTIVNLESDQRTVHV

>AT3G61830.1

MASVEGDDDFGSSSSRSYQDQLYTELWKVCAGPLVEVPRAQERVFYFPQGHMEQLVASTN

QGINSEEIPVFDLPPKILCRVLDVTLKAEHETDEVYAQITLQPEEDQSEPTSLDPPIVGP

TKQEFHSFVKILTASDTSTHGGFSVLRKHATECLPSLDMTQATPTQELVTRDLHGFEWRF

KHIFRGQPRRHLLTTGWSTFVSSKRLVAGDAFVFLRGENGDLRVGVRRLARHQSTMPTSV

ISSQSMHLGVLATASHAVRTTTIFVVFYKPRISQFIVGVNKYMEAIKHGFSLGTRFRMRF

EGEESPERIFTGTIVGSGDLSSQWPASKWRSLQVQWDEPTTVQRPDKVSPWEIEPFLATS

PISTPAQQPQSKCKRSRPIEPSVKTPAPPSFLYSLPQSQDSINASLKLFQDPSLERISGG

YSSNNSFKPETPPPPTNCSYRLFGFDLTSNSPAPIPQDKQPMDTCGAAKCQEPITPTSMS

EQKKQQTSRSRTKVQMQGIAVGRAVDLTLLKSYDELIDELEEMFEIQGQLLARDKWIVVF

TDDEGDMMLAGDDPWNEFCKMAKKIFIYSSDEVKKMTTKLKISSSLENEEYGNESFENRS

RG

>AT4G23980.1

MANRGGEYLYDELWKLCAGPLVDVPQAQERVYYFPQGHMEQLEASTQQVDLNTMKPLFVL

PPKILCNVMNVSLQAEKDTDEVYAQITLIPVGTEVDEPMSPDPSPPELQRPKVHSFSKVL

TASDTSTHGGFSVLRKHATECLPPLDMTQQTPTQELVAEDVHGYQWKFKHIFRGQPRRHL

LTTGWSTFVTSKRLVAGDTFVFLRGENGELRVGVRRANLQQSSMPSSVISSHSMHLGVLA

TARHATQTKTMFIVYYKPRTSQFIISLNKYLEAMSNKFSVGMRFKMRFEGEDSPERRYSG

TVIGVKDCSPHWKDSKWRCLEVHWDEPASISRPNKVSPWEIEPFVNSENVPKSVMLKNKR

PRQVSEVSALDVGITASNLWSSVLTQPHEFAQSCITSQWSSPQQCHRDANEDAKKSDWLN

NSYSVSNVAKDSTLNDQMVSPVEQKKPETTANYRLFGIDLMSSSLAVPEEKTAPMRPINI

SKPTMDSHSDPKSEISKVSEEKKQEPAEGSPKEVQSKQSSSTRSRTKVQMQGVPVGRAVD

LNALKGYNELIDDIEKLFDIKGELRSRNQWEIVFTDDEGDMMLVGDDPWPEFCNMVKRIF

IWSKEEVKKMTPGNQLRMLLREVETTLTTTSKTDNHSN

>AT4G23980.2

MANRGGEYLYDELWKLCAGPLVDVPQAQERVYYFPQGHMEQLEASTQQVDLNTMKPLFVL

PPKILCNVMNVSLQAEKDTDEVYAQITLIPVGTEVDEPMSPDPSPPELQRPKVHSFSKVL

TASDTSTHGGFSVLRKHATECLPPLDMTQQTPTQELVAEDVHGYQWKFKHIFRGQPRRHL

LTTGWSTFVTSKRLVAGDTFVFLRGENGELRVGVRRANLQQSSMPSSVISSHSMHLGVLA

TARHATQTKTMFIVYYKPRTSQFIISLNKYLEAMSNKFSVGMRFKMRFEGEDSPERRYSG

TVIGVKDCSPHWKDSKWRCLEVHWDEPASISRPNKVSPWEIEPFVNSENVPKSVMLKNKR

PRQVSEVSALGITASNLWSSVLTQPHEFAQSCITSQWSSPQQCHRDANEDAKKSDWLNNS

YSVSNVAKDSTLNDQMVSPVEQKKPETTANYRLFGIDLMSSSLAVPEEKTAPMRPINISK

PTMDSHSDPKSEISKVSEEKKQEPAEGSPKEVQSKQSSSTRSRTKVQMQGVPVGRAVDLN

ALKGYNELIDDIEKLFDIKGELRSRNQWEIVFTDDEGDMMLVGDDPWPEFCNMVKRIFIW

SKEEVKKMTPGNQLRMLLREVETTLTTTSKTDNHSN

>AT4G30080.1

MINVMNPMKGGTEKGLDPQLWHACAGGMVRMPPMNSKVFYFPQGHAENAYDCVDFGNLPI

PPMVLCRVLAIKYMADAESDEVFAKLRLIPLKDDEYVDHEYGDGEDSNGFESNSEKTPSF

AKTLTQSDANNGGGFSVPRYCAETIFPRLDYNAEPPVQTILAKDVHGDVWKFRHIYRGTP

RRHLLTTGWSNFVNQKKLVAGDSIVFMRAENGDLCVGIRRAKRGGIGNGPEYSAGWNPIG

GSCGYSSLLREDESNSLRRSNCSLADRKGKVTAESVIEAATLAISGRPFEVVYYPRASTS

EFCVKALDARAAMRIPWCSGMRFKMAFETEDSSRISWFMGTVSAVNVSDPIRWPNSPWRL

LQVAWDEPDLLQNVKRVNPWLVELVSNVHPIPLTSFSPPRKKMRLPQHPDYNNLINSIPV

PSFPSNPLIRSSPLSSVLDNVPVGLQGARHNAHQYYGLSSSDLHHYYLNRPPPPPPPSSL

QLSPSLGLRNIDTKNEKGFCFLTMGTTPCNDTKSKKSHIVLFGKLILPEEQLSEKGSTDT

ANIEKTQISSGGSNQNGVAGREFSSSDEGSPCSKKVHDASGLETGHCKVFMESDDVGRTL

DLSVLGSYEELSRKLSDMFGIKKSEMLSSVLYRDASGAIKYAGNEPFSEFLKTARRLTIL

TEQGSESVVV

>AT5G20730.1

MKAPSSNGVSPNPVEGERRNINSELWHACAGPLISLPPAGSLVVYFPQGHSEQVAASMQK

QTDFIPSYPNLPSKLICMLHNVTLNADPETDEVYAQMTLQPVNKYDRDALLASDMGLKLN

RQPNEFFCKTLTASDTSTHGGFSVPRRAAEKIFPALDFSMQPPCQELVAKDIHDNTWTFR

HIYRGQPKRHLLTTGWSVFVSTKRLFAGDSVLFIRDGKAQLLLGIRRANRQQPALSSSVI

SSDSMHIGVLAAAAHANANNSPFTIFYNPRWAAPAEFVVPLAKYTKAMYAQVSLGMRFRM

IFETEECGVRRYMGTVTGISDLDPVRWKNSQWRNLQIGWDESAAGDRPSRVSVWDIEPVL

TPFYICPPPFFRPRFSGQPGMPDDETDMESALKRAMPWLDNSLEMKDPSSTIFPGLSLVQ

WMNMQQQNGQLPSAAAQPGFFPSMLSPTAALHNNLGGTDDPSKLLSFQTPHGGISSSNLQ

FNKQNQQAPMSQLPQPPTTLSQQQQLQQLLHSSLNHQQQQSQSQQQQQQQQLLQQQQQLQ

SQQHSNNNQSQSQQQQQLLQQQQQQQLQQQHQQPLQQQTQQQQLRTQPLQSHSHPQPQQL

QQHKLQQLQVPQNQLYNGQQAAQQHQSQQASTHHLQPQLVSGSMASSVITPPSSSLNQSF

QQQQQQSKQLQQAHHHLGASTSQSSVIETSKSSSNLMSAPPQETQFSRQVEQQQPPGLNG

QNQQTLLQQKAHQAQAQQIFQQSLLEQPHIQFQLLQRLQQQQQQQFLSPQSQLPHHQLQS

QQLQQLPTLSQGHQFPSSCTNNGLSTLQPPQMLVSRPQEKQNPPVGGGVKAYSGITDGGD

APSSSTSPSTNNCQISSSGFLNRSQSGPAILIPDAAIDMSGNLVQDLYSKSDMRLKQELV

GQQKSKASLTDHQLEASASGTSYGLDGGENNRQQNFLAPTFGLDGDSRNSLLGGANVDNG

FVPDTLLSRGYDSQKDLQNMLSNYGGVTNDIGTEMSTSAVRTQSFGVPNVPAISNDLAVN

DAGVLGGGLWPAQTQRMRTYTKVQKRGSVGRSIDVNRYRGYDELRHDLARMFGIEGQLED

PQTSDWKLVYVDHENDILLVGDDPWEEFVNCVQSIKILSSAEVQQMSLDGNFAGVPVTNQ

ACSGGDSGNAWRGHYDDNSATSFNR

>AT5G20730.2

MKAPSSNGVSPNPVEGERRNINSELWHACAGPLISLPPAGSLVVYFPQGHSEQVAASMQK

QTDFIPSYPNLPSKLICMLHNVTLNADPETDEVYAQMTLQPVNKYDRDALLASDMGLKLN

RQPNEFFCKTLTASDTSTHGGFSVPRRAAEKIFPALDFSMQPPCQELVAKDIHDNTWTFR

HIYRGQPKRHLLTTGWSVFVSTKRLFAGDSVLFIRDGKAQLLLGIRRANRQQPALSSSVI

SSDSMHIGVLAAAAHANANNSPFTIFYNPRAAPAEFVVPLAKYTKAMYAQVSLGMRFRMI

FETEECGVRRYMGTVTGISDLDPVRWKNSQWRNLQIGWDESAAGDRPSRVSVWDIEPVLT

PFYICPPPFFRPRFSGQPGMPDDETDMESALKRAMPWLDNSLEMKDPSSTIFPGLSLVQW

MNMQQQNGQLPSAAAQPGFFPSMLSPTAALHNNLGGTDDPSKLLSFQTPHGGISSSNLQF

NKQNQQAPMSQLPQPPTTLSQQQQLQQLLHSSLNHQQQQSQSQQQQQQQQLLQQQQQLQS

QQHSNNNQSQSQQQQQLLQQQQQQQLQQQHQQPLQQQTQQQQLRTQPLQSHSHPQPQQLQ

QHKLQQLQVPQNQLYNGQQAAQQHQSQQASTHHLQPQLVSGSMASSVITPPSSSLNQSFQ

QQQQQSKQLQQAHHHLGASTSQSSVIETSKSSSNLMSAPPQETQFSRQVEQQQPPGLNGQ

NQQTLLQQKAHQAQAQQIFQQSLLEQPHIQFQLLQRLQQQQQQQFLSPQSQLPHHQLQSQ

QLQQLPTLSQGHQFPSSCTNNGLSTLQPPQMLVSRPQEKQNPPVGGGVKAYSGITDGGDA

PSSSTSPSTNNCQISSSGFLNRSQSGPAILIPDAAIDMSGNLVQDLYSKSDMRLKQELVG

QQKSKASLTDHQLEASASGTSYGLDGGENNRQQNFLAPTFGLDGDSRNSLLGGANVDNGF

VPDTLLSRGYDSQKDLQNMLSNYGGVTNDIGTEMSTSAVRTQSFGVPNVPAISNDLAVND

AGVLGGGLWPAQTQRMRTYTKVQKRGSVGRSIDVNRYRGYDELRHDLARMFGIEGQLEDP

QTSDWKLVYVDHENDILLVGDDPWEEFVNCVQSIKILSSAEVQQMSLDGNFAGVPVTNQA

CSGGDSGNAWRGHYDDNSATSFNR

>AT5G20730.3

MKAPSSNGVSPNPVEGERRNINSELWHACAGPLISLPPAGSLVVYFPQGHSEQVAASMQK

QTDFIPSYPNLPSKLICMLHNVTLNADPETDEVYAQMTLQPVNKYDRDALLASDMGLKLN

RQPNEFFCKTLTASDTSTHGGFSVPRRAAEKIFPALDFSMQPPCQELVAKDIHDNTWTFR

HIYRGQPKRHLLTTGWSVFVSTKRLFAGDSVLFIRDGKAQLLLGIRRANRQQPALSSSVI

SSDSMHIGVLAAAAHANANNSPFTIFYNPRWAAPAEFVVPLAKYTKAMYAQVSLGMRFRM

IFETEECGVRRYMGTVTGISDLDPVRWKNSQWRNLQIGWDESAAGDRPSRVSVWDIEPVL

TPFYICPPPFFRPRFSGQPGMPDDETDMESALKRAMPWLDNSLEMKDPSSTIFPGLSLVQ

WMNMQQQNGQLPSAAAQPGFFPSMLSPTAALHNNLGGTDDPSKLLSFQTPHGGISSSNLQ

FNKQNQQAPMSQLPQPPTTLSQQQQLQQLLHSSLNHQQQQSQSQQQQQQQQLLQQQQQLQ

SQQHSNNNQSQSQQQQQLLQQQQQQQLQQQHQQPLQQQTQQQQLRTQPLQSHSHPQPQQL

QQHKLQQLQVPQNQLYNGQQAAQQHQSQQASTHHLQPQLVSGSMASSVITPPSSSLNQSF

QQQQQQSKQLQQAHHHLGASTSQSSVIETSKSSSNLMSAPPQETQFSRQVEQQQPPGLNG

QNQQTLLQQKAHQAQAQQIFQQSLLEQPHIQFQLLQRLQQQQQQQFLSPQSQLPHHQLQS

QQLQQLPTLSQGHQFPSSCTNNGLSTLQPPQMLVSRPQEKQNPPVGGGVKAYSGITDGGD

APSSSTSPSTNNCQISSSGFLNRSQSGPAILIPDAAIDMSGNLVQDLYSKSDMRLKQELV

GQQKSKASLTDHQLEASASGTSYGLDGGENNRQQNFLAPTFGLDGDSRNSLLGGANVDNG

FVPDTLLSRGYDSQKDLQNMLSNYGGVTNDIGTEMSTSAVRTQSFGVPNVPAISNDLAVN

DAGVLGGGLWPAQTQRMRTYTKVQKRGSVGRSIDVNRYRGYDELRHDLARMFGIEGQLED

PQTSDWKLVYVDHENDILLVGDDPWEEFVNCVQSIKILSSAEVQQMSLDGNFAGVPVTNQ

ACSGATSFNR

>AT5G37020.1

MKLSTSGLGQQGHEGEKCLNSELWHACAGPLVSLPSSGSRVVYFPQGHSEQVAATTNKEV

DGHIPNYPSLPPQLICQLHNVTMHADVETDEVYAQMTLQPLTPEEQKETFVPIELGIPSK

QPSNYFCKTLTASDTSTHGGFSVPRRAAEKVFPPLDYTLQPPAQELIARDLHDVEWKFRH

IFRGQPKRHLLTTGWSVFVSAKRLVAGDSVIFIRNEKNQLFLGIRHATRPQTIVPSSVLS

SDSMHIGLLAAAAHASATNSCFTVFFHPRASQSEFVIQLSKYIKAVFHTRISVGMRFRML

FETEESSVRRYMGTITGISDLDSVRWPNSHWRSVKVGWDESTAGERQPRVSLWEIEPLTT

FPMYPSLFPLRLKRPWHAGTSSLPDGRGDLGSGLTWLRGGGGEQQGLLPLNYPSVGLFPW

MQQRLDLSQMGTDNNQQYQAMLAAGLQNIGGGDPLRQQFVQLQEPHHQYLQQSASHNSDL

MLQQQQQQQASRHLMHAQTQIMSENLPQQNMRQEVSNQPAGQQQQLQQPDQNAYLNAFKM

QNGHLQQWQQQSEMPSPSFMKSDFTDSSNKFATTASPASGDGNLLNFSITGQSVLPEQLT

TEGWSPKASNTFSEPLSLPQAYPGKSLALEPGNPQNPSLFGVDPDSGLFLPSTVPRFASS

SGDAEASPMSLTDSGFQNSLYSCMQDTTHELLHGAGQINSSNQTKNFVKVYKSGSVGRSL

DISRFSSYHELREELGKMFAIEGLLEDPLRSGWQLVFVDKENDILLLGDDPWESFVNNVW

YIKILSPEDVHQMGDHGEGSGGLFPQNPTHL

>AT5G37020.2

MKLSTSGLGQQGHEGEKCLNSELWHACAGPLVSLPSSGSRVVYFPQGHSEQVAATTNKEV

DGHIPNYPSLPPQLICQLHNVTMHADVETDEVYAQMTLQPLTPEEQKETFVPIELGIPSK

QPSNYFCKTLTASDTSTHGGFSVPRRAAEKVFPPLDYTLQPPAQELIARDLHDVEWKFRH

IFRGQPKRHLLTTGWSVFVSAKRLVAGDSVIFIRNEKNQLFLGIRHATRPQTIVPSSVLS

SDSMHIGLLAAAAHASATNSCFTVFFHPRASQSEFVIQLSKYIKAVFHTRISVGMRFRML

FETEESSVRRYMGTITGISDLDSVRWPNSHWRSVKVGWDESTAGERQPRVSLWEIEPLTT

FPMYPSLFPLRLKRPWHAGTSSLPDGRGDLGSGLTWLRGGGGEQQGLLPLNYPSVGLFPW

MQQRLDLSQMGTDNNQQYQAMLAAGLQNIGGGDPLRQQFVQLQEPHHQYLQQSASHNSDL

MLQQQQQQQASRHLMHAQTQIMSENLPQQNMRQEVSNQPAGQQQQLQQPDQNAYLNAFKM

QNGHLQQWQQQSEMPSPSFMKSDFTDSSNKFATTASPASGDGNLLNFSITGQSVLPEQLT

TEGWSPKASNTFSEPLSLPQAYPGKSLALEPGNPQNPSLFGVDPDSGLFLPSTVPRFASS

SGDAEASPMSLTDSGFQNSLYSCMQDTTHELLHGAGQINSSNQTKNFVKVYKSGSVGRSL

DISRFSSYHELREELGKMFAIEGLLEDPLRSGWQLVFVDKENDILLLGDDPWE

>AT5G60450.1

MEFDLNTEIAEVEEEENDDVGVGVGGGTRIDKGRLGISPSSSSSCSSGSSSSSSSTGSAS

SIYSELWHACAGPLTCLPKKGNVVVYFPQGHLEQDAMVSYSSPLEIPKFDLNPQIVCRVV

NVQLLANKDTDEVYTQVTLLPLQEFSMLNGEGKEVKELGGEEERNGSSSVKRTPHMFCKT

LTASDTSTHGGFSVPRRAAEDCFAPLDYKQQRPSQELIAKDLHGVEWKFRHIYRGQPRRH

LLTTGWSIFVSQKNLVSGDAVLFLRDEGGELRLGIRRAARPRNGLPDSIIEKNSCSNILS

LVANAVSTKSMFHVFYSPRATHAEFVIPYEKYITSIRSPVCIGTRFRMRFEMDDSPERRC

AGVVTGVCDLDPYRWPNSKWRCLLVRWDESFVSDHQERVSPWEIDPSVSLPHLSIQSSPR

PKRPWAGLLDTTPPGNPITKRGGFLDFEESVRPSKVLQGQENIGSASPSQGFDVMNRRIL

DFAMQSHANPVLVSSRVKDRFGEFVDATGVNPACSGVMDLDRFPRVLQGQEICSLKSFPQ

FAGFSPAAAPNPFAYQANKSSYYPLALHGIRSTHVPYQNPYNAGNQSSGPPSRAINFGEE

TRKFDAQNEGGLPNNVTADLPFKIDMMGKQKGSELNMNASSGCKLFGFSLPVETPASKPQ

SSSKRICTKVHKQGSQVGRAIDLSRLNGYDDLLMELERLFNMEGLLRDPEKGWRILYTDS

ENDMMVVGDDPWHDFCNVVWKIHLYTKEEVENANDDNKSCLEQAALMMEASKSSSVSQPD

SSPTITRV

>AT5G62000.1

MASSEVSMKGNRGGDNFSSSGFSDPKETRNVSVAGEGQKSNSTRSAAAERALDPEAALYR

ELWHACAGPLVTVPRQDDRVFYFPQGHIEQVEASTNQAAEQQMPLYDLPSKLLCRVINVD

LKAEADTDEVYAQITLLPEANQDENAIEKEAPLPPPPRFQVHSFCKTLTASDTSTHGGFS

VLRRHADECLPPLDMSRQPPTQELVAKDLHANEWRFRHIFRGQPRRHLLQSGWSVFVSSK

RLVAGDAFIFLRGENGELRVGVRRAMRQQGNVPSSVISSHSMHLGVLATAWHAISTGTMF

TVYYKPRTSPSEFIVPFDQYMESVKNNYSIGMRFKMRFEGEEAPEQRFTGTIVGIEESDP

TRWPKSKWRSLKVRWDETSSIPRPDRVSPWKVEPALAPPALSPVPMPRPKRPRSNIAPSS

PDSSMLTREGTTKANMDPLPASGLSRVLQGQEYSTLRTKHTESVECDAPENSVVWQSSAD

DDKVDVVSGSRRYGSENWMSSARHEPTYTDLLSGFGTNIDPSHGQRIPFYDHSSSPSMPA

KRILSDSEGKFDYLANQWQMIHSGLSLKLHESPKVPAATDASLQGRCNVKYSEYPVLNGL

STENAGGNWPIRPRALNYYEEVVNAQAQAQAREQVTKQPFTIQEETAKSREGNCRLFGIP

LTNNMNGTDSTMSQRNNLNDAAGLTQIASPKVQDLSDQSKGSKSTNDHREQGRPFQTNNP

HPKDAQTKTNSSRSCTKVHKQGIALGRSVDLSKFQNYEELVAELDRLFEFNGELMAPKKD

WLIVYTDEENDMMLVGDDPWQEFCCMVRKIFIYTKEEVRKMNPGTLSCRSEEEAVVGEGS

DAKDAKSASNPSLSSAGNS

>AT5G62000.2

MASSEVSMKGNRGGDNFSSSGFSDPKETRNVSVAGEGQKSNSTRSAAAERALDPEAALYR

ELWHACAGPLVTVPRQDDRVFYFPQGHIEQVEASTNQAAEQQMPLYDLPSKLLCRVINVD

LKAEADTDEVYAQITLLPEANQDENAIEKEAPLPPPPRFQVHSFCKTLTASDTSTHGGFS

VLRRHADECLPPLDMSRQPPTQELVAKDLHANEWRFRHIFRGQPRRHLLQSGWSVFVSSK

RLVAGDAFIFLRGENGELRVGVRRAMRQQGNVPSSVISSHSMHLGVLATAWHAISTGTMF

TVYYKPRTSPSEFIVPFDQYMESVKNNYSIGMRFKMRFEGEEAPEQRFTGTIVGIEESDP

TRWPKSKWRSLKVRWDETSSIPRPDRVSPWKVEPALAPPALSPVPMPRPKRPRSNIAPSS

PDSSMLTREGTTKANMDPLPASGLSRVLQGQEYSTLRTKHTESVECDAPENSVVWQSSAD

DDKVDVVSGSRRYGSENWMSSARHEPTYTDLLSGFGTNIDPSHGQRIPFYDHSSSPSMPA

KRILSDSEGKFDYLANQWQMIHSGLSLKLHESPKVPAATDASLQGRCNVKYSEYPVLNGL

STENAGGNWPIRPRALNYYEEVVNAQAQAQAREQVTKQPFTIQEETAKSREGNCRLFGIP

LTNNMNGTDSTMSQRNNLNDAAGLTQIASPKVQDLSDQSKGSKSTNDHREQGRPFQTNNP

HPKDAQTKTNSSRSCTKVHKQGIALGRSVDLSKFQNYEELVAELDRLFEFNGELMAPKKD

WLIVYTDEENDMMLVGDDPWQEFCCMVRKIFIYTKEEVRKMNPGTLSCRSEEEAVVGEGS

DAKDAKSASNPSLSSAGNS

>AT5G62000.3

MASSEVSMKGNRGGDNFSSSGFSDPKETRNVSVAGEGQKSNSTRSAAAERALDPEAALYR

ELWHACAGPLVTVPRQDDRVFYFPQGHIEQVEASTNQAAEQQMPLYDLPSKLLCRVINVD

LKAEADTDEVYAQITLLPEANQDENAIEKEAPLPPPPRFQVHSFCKTLTASDTSTHGGFS

VLRRHADECLPPLDMSRQPPTQELVAKDLHANEWRFRHIFRGQPRRHLLQSGWSVFVSSK

RLVAGDAFIFLRGENGELRVGVRRAMRQQGNVPSSVISSHSMHLGVLATAWHAISTGTMF

TVYYKPRTSPSEFIVPFDQYMESVKNNYSIGMRFKMRFEGEEAPEQRFTGTIVGIEESDP

TRWPKSKWRSLKVRWDETSSIPRPDRVSPWKVEPALAPPALSPVPMPRPKRPRSNIAPSS

PDSSMLTREGTTKANMDPLPASGLSRVLQGQEYSTLRTKHTESVECDAPENSVVWQSSAD

DDKVDVVSGSRRYGSENWMSSARHEPTYTDLLSGFGTNIDPSHGQRIPFYDHSSSPSMPA

KRILSDSEGKFDYLANQWQMIHSGLSLKLHESPKVPAATDASLQGRCNVKYSEYPVLNGL

STENAGGNWPIRPRALNYYEEVVNAQAQAQAREQVTKQPFTIQEETAKSREGNCRLFGIP

LTNNMNGTDSTMSQRNNLNDAAGLTQIASPKVQDLSDQSKGSKSTNDHREQGRPFQTNNP

HPKDAQTKTNSSRSCTKVHKQGIALGRSVDLSKFQNYEELVAELDRLFEFNGELMAPKKD

WLIVYTDEENDMMLVGDDPWQEFCCMVRKIFIYTKEEVRKMNPGTLSCRSEEEAVVGEGS

DAKDAKSASNPSLSSAGNS

>AT5G62000.4

MASSEVSMKGNRGGDNFSSSGFSDPKETRNVSVAGEGQKSNSTRSAAAERALDPEAALYR

ELWHACAGPLVTVPRQDDRVFYFPQGHIEQVEASTNQAAEQQMPLYDLPSKLLCRVINVD

LKAEADTDEVYAQITLLPEANQDENAIEKEAPLPPPPRFQVHSFCKTLTASDTSTHGGFS

VLRRHADECLPPLDMSRQPPTQELVAKDLHANEWRFRHIFRGQPRRHLLQSGWSVFVSSK

RLVAGDAFIFLRGENGELRVGVRRAMRQQGNVPSSVISSHSMHLGVLATAWHAISTGTMF

TVYYKPRTSPSEFIVPFDQYMESVKNNYSIGMRFKMRFEGEEAPEQRFTGTIVGIEESDP

TRWPKSKWRSLKVRWDETSSIPRPDRVSPWKVEPALAPPALSPVPMPRPKRPRSNIAPSS

PDSSMLTREGTTKANMDPLPASGLSRVLQGQEYSTLRTKHTESVECDAPENSVVWQSSAD

DDKVDVVSGSRRYGSENWMSSARHEPTYTDLLSGFGTNIDPSHGQRIPFYDHSSSPSMPA

KRILSDSEGKFDYLANQWQMIHSGLSLKLHESPKVPAATDASLQGRCNVKYSEYPVLNGL

STENAGGNWPIRPRALNYYEEVVNAQAQAQAREQVTKQPFTIQEETAKSREGNCRLFGIP

LTNNMNGTDSTMSQRNNLNDAAGLTQIASPKVQDLSDQSKGSKSTNDHREQGRPFQTNNP

HPKDAQTKTNSSRSCTKVHKQGIALGRSVDLSKFQNYEELVAELDRLFEFNGELMAPKKD

WLIVYTDEENDMMLVGDDPWQEFCCMVRKIFIYTKEEVRKMNPGTLSCRSEEEAVDGGRL

VETGEQNGMMGLR

>CmARF18

MITFMDSKEKVKEMEKCLDPQLWHACAGGMVQMPPVNARVFYFPQGHAEHACAPVDFSNCSKVPPYTLCRVSAIKFLADPDTDEVFAKLRLIPINGSELEFEDDGIGRLNGSEQDKPTSFAKTLTQSDANNGGGFSVPRYCAETIFPRLDYSADPPVQTILAKDVHGETWKFRHIYRGTPRRHLLTTGWSTFVNHKKLVAGDSIVFLRAENGDLCVGIRRAKRGIGDGPEPPCGWNPAGGNCAVPYGAFSTFLREDENRVNRTNGKGKVKAESVIEAATLAANGQPFEIVYYPRASTPEFCVKAGLVKAALQIRWCSGMRFKMAFETEDSLRISWFMGTINTVQAADPLRWPESPWRLLQVTWDEPDLLQNVKRVSPWLVELVSNISPIHLAPFSPPRKKFRYPQHPDFPLDNQPSMSPFASYLHGPGSPFGCPPDNNPAGMQGARHAHFGLSLSDFHLSKLQSGLFPIRYRSLDPAAGSTRLSGNAMTEKPSMSENVSCLLTMAHSTQTSKKFDNVKTPQLILFGRPILTELQMSQSCSGDTVSPVGTGNSSSDGNGSGSALHQQGLPERSSCENFQWYKDNRQDVEPNLDTGHCKVFMESEDVGRTLDLSSLGSYEELYRKLGNMFGIDNSEMLNHVLYRDISGAVKHVGDEQFSEFIKTARRLTILTDSGSNNVGATT

>CmARF02

MEIDLNLTASDVGKNAYCNGNCEEGRCNCCLSSSTSSCSSNSSPALVSSSTYLELWHACAGPLTSLPKKGNVVVYFPQGHLEQIASASPFSPMEMRTFDLQPQILCRVINVHLLANKENDEVYTQLTLLPLPEFLGTGLEGKELEELALNGADGDGSGVSPTRSTPHMFCKTLTASDTSTHGGFSVPRRAAEDCFPPLDYTQLRPSQELIAKDLHGVEWRFKHIYRGQPRRHLLTTGWSLFVSQKNLISGDAVLFLRGENGELRLGIRRAVRPRNGLPNSIVGNQNSCADDLARVVKAISTKSIFDVFYNPRAYHAQFVISCQKYVKSINNPVTVGTRFKMRFEMDDSPERRFNGVVLGIGDMDPLRWPNSKWRCLTVRWDKDGDHQERVSPWEIESSVSLPPLSVQSSPRLKKLRTSLQAAPPNAFAVGRGGFMDFEDSIRSSKVLQGQENVGIVSPFYGRDTTKRSLEFEVRSSAHQASGGAEKYRGDYVKVHPNSSFTGFMESERFLKVLQGQEICSLRPQTRKPEHCLGVWGKFNLSDNSFNTFHSPNSSFYHMASNGAQNMYFPPSEFYSTGQAAAVMRSNDGNFPRESALFGPSVDASVMSTTSGSNIRNSKDENVNENSTGCRLFGFSLTTETATNMQSSGKRSCTKVHKQGSLVGRAIDLSRLNGYSDLLSELERLFCMEGLLKDPDKGWRVLYTDNENDVMVVGDYPWLDFCDAVSKIHIYTQEEVEKMTNGVISDDTQSCLDQAPLCMEASKSSSVGQPDSSPTVISITFASSKSAQGAAHEYRL

>CmARF03

MLKRKKKKGNGFVLDGFLFFLVFFVYWALHLGENAIIRMKEADKNLDPQLWHACAGGMVQMPAINSKVFYFPQGHAEHAQAAVDFTSSLRIPPLIPCRVLAVKFLADHETDEVYANVRLVPLANNEINCEEEGGFGSNGSENNMEKPASFAKTLTQSDANNGGGFSVPRYCAETIFPRLDYTADPPVQTVIAKDVHGEVWKFRHIYRGTPRRHLLTTGWSSFVNQKKLVAGDSIVFLRSKNGDLCVGIRRAKRAIGCAADHPYGWNPGGGNSIPPYGGLTMFLRDEDNKLSRKGSLSSNGGGNLRGKGKVRPESVIEAAALAASGQAFEVVYYPRASTPEFCVKASSVRAAMRIQWCSGMRFKMPFETEDSSRISWFMGTISSVQVADPIRWPNSPWRLLQWFIGPLSCHFLYQPPRSKFQCILKRLVYYYRLNGKVTWDEPDLLQNVKRVSPWLVELVSNMPVIQLSPFSPPRKKFRLPQHPDFPLDSQFQLSSFPSNTLRPSSPMCCLSDNTSVGIQGARHTQFGISSSDFHLNSKLQLGLLPSSFQQLDFHSRISHRSVTDHRGSGSQNSSALLNAENTGLKLERSDSVKKYQFLLFGQPILTEQQITRSSSGDVHSPRTEKSSLDVNLERVKFLSDGSGYTFKQQQISPNKSPGAGFPWYQGYQATELGLDIGHCKVFMESEDVGRTLNLSVIGSYEELYRRLANMFGMEKPDILSHVLYQDATGAVKQAGDKPFSDFIKTARRLTILTDSGSDKMVGRRLMDGLRSGENGLDSSNKTGPLSIFA

>CmARF23

MTFHGGDSYTQSCVSAQGRGRDDLYMELWRACAGPLVDIPRVDEKVFYFPQGHMEQLEASTNLELNKRIPLFNLSSKILCRVIHIDPLADHESDEVYAQITLLPESNQSEPRSLDPCPPEPVKPVVHSFCKVLTASDTSTHGGFSVLRKHANECLPPLVRPDMTQATPTQDLVAKDLHGYEWRFKHIFRGQPRRHLLTTGWSTFVTSKRLTAGDSFVFLSLSMYANDCNFFLLTLLEKLYNDLYAKLACKYLFLWTLWGDNGQLRVGVRRRAQQQSSMPPSVISSQSMHLGVLATASHAVTTQTRFVVYYKPRTCQFIISLNKYLEAVNYKFSLGMRFNMSFEGEDSPERRFSGTIIGAGDISPHWANSSWRSLRVQWDEQTSILRPDRVSPWDIEPLTSSAVSSLSQPVSKNKRPRQSTETPAHDGADLTKSAHWDSGLAQSHDGKQFGNATESRLGENNELCHHSEIDTISNSTCVSRTQTDGTWLFSTQSNGYKDPVNDTAQDYKTVPVCGWSILSGPSKVSDDQILDPTENGRKGETVASCRLFGIDLNHLAKLPAEKASSQPSSMSSHTDGRISTLSVAQSESKSDNIEASIDRKLEPLQASLKETQSYSLLLMFGVQLCQKVHMHGMAVGRAVDLTILEGYDQLIDELEKMFDVRGQLSARDKWEIVYTDDEGDMMLVGDDPWEEFCNMVRRIFICSREQVKKMSSGSKQLASIEVEGAVVIPDSPAI

>CmARF10

MLSIESDWVKLDMKLSTSGFGQQDHEGGEKKCLNSELWHACAGPLVSLPTSGTRVVYFPQGHSEQVAATTNKEVDGHIPNYPNLPPQLICQLHNVTMHADVETDEVYAQMTLQPLTAQEQKDTFLPMELGIPSRQPTNYFCKTLTASDTSTHGGFSVPRRAAEKVFPPLDFSQQPPAQELIARDLHDVEWKFRHIFRGKSPYVLSICGNKCQSFSVEGQPKRHLLTTGWSVFVSAKRLVAGDSVLFIWNEKNQLLLGIRRATRPQTVMPSSVLSSDSMHIGLLAAAAHAAATNSCFTVFYNPRASPSEFVIPLTKYVKAVFHTRVSVGMRFRMLFETEESSVRRYMGTITGISDLDPVRWSNSHWRSVKVFQLYFYNSSTKTLAPFAITLSFSLLNVVRYAKIFASSRNDYFLYWCSNDFVLKLQQFSVSFSTMKVGWDESTAGERQPRVSLWEIEPLTTFPMYPSLFPLRLKRPWHPGVSSVHGIAYAHGSRRFVMVYFLTDNRDDASNGLMWLRGGVGEQALHSLNLQSAGSLPWLQQRLDSSMFGNDHNQQYQAMLAAGMPNLGGVDMLRQQIMHLQQPFQYAQQAGLHNSLLQLQQQQLVQQSMPQNILQAPTQVMAENLPQHILQQTLQNQPEDLPNQQQHTYHDTVQVQSNQFHQGGHSNVPSPTFPRSNLMDSNSNYPESISSRRNIIASSCAEGTGNLSNIYRSGQSILTEQLPQQSPVSKNAHSPVDAHPNSMSFPFSGRDSILELGNCNSDSPSPTLFGVNIDSSGLLLPSNVPTYASPSIAPDSTSMPLGDSGFQNSLYNCVQGSSELLHNSGQVDPSNPTQTFVKVYKTGSIGRSLDISRFSSYQELREELAQMFGIEGQLEDPRRSGWQLVFVDRENDALLLGDDPWEAFVNNVWYIKILSPQDVQELREQVIESFNPIDGQRLTSGGNEAENVSGLPSVDRDQDEASLCTSMAELSPISVSNLSNRIHFSSSSPPINSSQLFSITAKRCSNCRSISRRLGLGLIALMASSSVASTPVRIANALDLKLIAPEQSFEEAESGIRGHAEAVLQVRGLIESESWKEAQKQLRTSSPLLKQDLYTIIEYKPPAERPPLRKLYSLLFNNVTRMDYAARDKDAERVRQCYDNIVMAISDILSRI

>CmARF11

MNRGMDSVIEPMRNVEKCLDSQLWHACAGGMVQMPSVNSKVVYFPQGHAEHAQGNVDFGNARIPSLIPCRVSATKYMADLETDEIFAKISLLPLRNNEFNLDDDDELLGHDDIRNQEKPSSFAKTLTQSDANNGGGFSVPRYCAETIFPPLDYSAEPPVQTILAKDVHGEIWKFRHIYRGTPRRHLLTTGWSNFVNQKKLVAGDSIVFLRAETGDLCVGVRRAKRGIGCGIDYSPGWNPANSGSSLMGYSDFMRENEGRMVRRHSNGNLNGRVKVESVIEAATLAASGKRFEIVYYPCAGTPEFVVRASTVRSAMQIHWYSAMRFKMPFETEDSSRISWFMGTISSIQVADPVRWPDSPWRMLQVSWDEPDLLQNVKSVNPWLVEVVVNMPAIHVSPFLPPRKKPRYPLPAETAVFGHLPMPSFSTNFFEATNPFQSITASNIPAGIQGARHTQFGLSSSNLQISIPPLGQFPASLKHLEGATPIPVVRGEDSFGGTKSPDNQSHWLTVGNHIQSSKESKETKPDHIILFGQLILPNQQSSNSSSGDTTNASDANLEKASNLSDGSGLSSQQNGSLDNSSDGGSTSYNGPNKTGLSLDIGHCEVFMELENVSHTLDLSVLGSYEELYRKLGNMFSVGRSEMLNSVLYQDALGATKQAGDEPFR

>CmARF12

MTSAEASIKPNSASAFKNLADSTKAPSDPPNALSAKDADIALYTELWNACAGPLVSVPRENERVFYFPQGHIEQVEASTNQVADQQMPVYDLPSKILCRIINVCLKAEPDTDEVFAQITLLPEANQDEDSVDKEPPPPPPRRFHVHSFCKTLTASDTSTHGGFSVLRRHADECLPPLDMSQQPPTQELVAKDLLGNEWRFRHIFRGQPRRHLLQSGWSVFVSSKRLVAGDAFIFLRGENRELRVGVRRAMRQHGNVPSSVISSHSMHLGVLATAWHAISTGTMFTVYYKPRTSPSEFVVPYDQYMESIKKSYTIGTRFKMRFEGEEAPEQRFTGTVIGCEDADPKRWKDSKWRCLKVRWDETSTISRPEKVSPWKIEPALAPPALNPLPMTRPKRHRSNMVPTSPDSSVLTREGPSRMIIDHSPASAFTRVLQGQEFSTLRGNLIDGNESDTAEKSVMWSPSLDDEKIDVVSTSKKHGADSWMPAGSGEPTYADLLSGFGANIDSSCGVRATLGDPAIVTANSIRKHAVYQDGKFNFLGGGSSWSVLPSGLSLNLVDSSQKAHIQAGDLPYQMRGNATFNGVGDHSMAQCYRIDHSMAHCYRIEQPNGNWSMPPPSSHFDYPVHSTELTSKPMLFQNQDILNLKDGNCKLFGISLVKNPAISDPAELHRNVMNEADVIHPNRHPIHLNESELSRGSNLADKSLAINVADKLQQACTPNLKDSQSKSQGSSTRSCTKVHKQGIALGRSVDLSKFNNYDELIAELDQLFEFGGELLPPKKSWLVVYTDDEGDMMLVGDDPWQEFCGMVRKIFIYTREEVQKMNPRSLTLKGDENPSVEGEEAKEIKSPAVPSMSAPES

>CmARF01

MGSVDEKLKTIGGFISNAPQPNLLDDMKLLKEMQDQTGARKAINSELWHACAGPLVSLPHMGSLVYYFPQGHSEQVAVSTKRTATSQIPNYPNLPSQLMCQVHNVTLHADKDSDEIYAQMSLQPVNSEKDVFIVPDFGLRPSKHPNEFFCKTLTASDTSTHGGFSVPRRAAEKLFPPLDYTMQPPTQELVVRDLHDNTWTFRHIYRGQPKRHLLTTGWSLFVGAKRLRAGDSVLFIRDEKSQLLVGVRRANRQQSTLPSSVLSADSMHIGVLAAAAHAAANRSPFTIFYNPRACPSEFVIPLAKYRKCVYGTQLSAGMRFGMMFETEESGKRRYMGTIVGISDLDPLRWPGSKWRNLQYSLACFSAATLIILSQVEWDEPGCCDKQNRVSSWDIETPESLFIFPSLTSGLKRPLHGGFLGETDWGSLVKRPMLRVPENVSYAPTLCSEPLMKMLLRPQLVNLHGTTLQQDMMGGMQPVTNPKMQQQQVTSSSPQHQNHHQPAPSPSTDPIISNSSPKANEPGKVQDSAAIESEAPTGAEADKSKYEREVSTCQSNPLPPVECGEDKLTGNEVNMQTLVNQLSFVNQNQIPMQLQSVSSWPMQPQLESLIQHPQPIDTPQPEFPNSNGLISSMDGDGCLINPTCLPLSGVMRSPGNLSMLGLQESSTVFSEALNFPIPSTGQEMWDPLSNIRFSSQTNHLVSFSHPDASNLICMANANVTRDVSDESNNQSGIYGCSNLEISNGGNTLVDPAVSSTILDDYCTLKDADFPHPSDCLAGNFSSSQDVQSQITSASLGDSQAFSRQEFHDNSGGTSSCNVDFDEGNLLQNGSWKQVVPPMRTYTKVGICGFSTNYLPYYMVQKAGSVGRSIDVTSFKNYEELCSAIECMFGLEGLLNDPRGSGWKLVYVDYENDVLLIGDDPWEEFVSCVRCIRILSPSEVQQMSEEGMKLLNSAMMQGMNCSISEGGRA

>CmARF15

MSVFWGDSFAPELFSLTYMRLGMKAPPNGFLPNSGEGERKNINSELWHACAGPLVSLPPVGSLVVYFPQGHSEQVAASMNKETDFIPSYPNLPSKLICMLHNVTLHADLETDEVYAQMTLQPVNKYEKEGLLASDIGLKQNRQPAEFFCKTLTASDTSTHGGFSVPRRAAEKIFPPLDYSMQPPAQELVARDLHDNSWTFRHIYRGQPKRHLLTTGWSVFVSTKRLFAGDSVLFIRDEKSQLLLGIRRANRQQPALSSSVISSDSMHIGILASAAHAAANNSPFTIFYNPRASPSEFVIPLAKYNKAMYAQVSLGMRFRMMFETEESGVRRYMGTITGISDMDSVRWKNSQWRNLQVGWDESAAGERPNRVSIWEVEPVVTPFYICPPPFFRPKFPKQPGMPDDESEIENAFKRAMPWFGDEFGMKDASSSIFPGLSLVQWMSMQHNNQFPAAQSGILPSMVAPSALHGTLTNDESSKLLSFQAPVMSSPNLQFSKANQQNQVAQLPPTTWSQQQQVQQLLQVPASQQLQQQQLPLQQELQQQVPQQQQLQMQQPQRQQQASQSALLNNGVTAPNHLPNQTLQQPLVYSQLQQQQLLAGNIQSHQTSQPSNKNLFQTTSLSQEVQFQPQIEQQPSLLPKHQQPTQVQQAPLQLMQQSLSQRAQQQPQVQQFSQPVPTEQQLQLQLLQKLQQQQQQHPLLSPASPLLPPQLLQQQNVHQQNQQLPPLPLSNQHQFSTSGGSLQTEKLNSNGFSSLGLMQSQQAPITHSHNQFKPTMAIRAYSGLTDGDAPSCSTSPSTNNCQVSASNMLNKNQQGAATLGGDPVAEPATNLAQDLQSKPDLRIKHEFPSSKGLDQLKYKGTVPDQLEACSSGTSYCLDAGTIQQTFPLPTCLDNDVQSQPRNNIPFSNSMDGLAPDTLLSRGYDSQKDLQNLLSNYGGGVARDIETELSTAAISSQSFGVPNLPFKSDCSNDVNVNEAGALSSGLWANHSQRMRTYTKVQKRGSVGRCIDVTRYKGYDELRHDLARMFGIEGQLEDPQRTDWKLVYVDHETDILLVGDDPWEEFVSCVQSIKILSSAEVQQMSLDGNLGHIQAPNQACSGTDSGNAWRGQYDDNSAASFNR

>CmARF19

MTLSEQNPAEFCKETKEFCKFRPVFGVFDCFCLVGLEGEGLFEELWKACAGPLVEVPFNGERVFYFPQGHMEQLEESTNHELNHQIPHFDLPSKILCRVVNIRLLAEKETDEVYAQITLQPEADQSEPQCPDPEPPERTRPTVHSFCKILTASDTSTHGGFSVLKKHATECLPPLDMSQSTPTQELAAKDLHGHEWKFKHIFRGTLQLIMMTKAVCQPRRHLLTTGWSTFVVSKRLVAGDAFVFLRGDNGELRVGVRRQARQKSVMPSSVISSHSMHLGVLATASHAVRTQTFFVVYYKPRTSQFIIGLNKYLETIKNRYEVGMRFKMRFEGEESPERRFTGTIVGVGDISPVWSDSKWRSLKVNIIQWDEAAAIQRPERVSPWEIEPFVPSASLNFTHPSIKSKRARPVEVPPPGKRENELLFRSKRLASSLSLCLIPMYVDCGVVENTSSSTPTGFWLHGSTIPHEIPQLSGANDVPSSGNQVVWSLWQQTLDVNVDSSRSHCNPMAHVEGIWPSPPPPVCNNSLKPLPSPYSPSSTSKPSSELIEHDQSEKGNKPDISLGCRIFGIDLKKNSSIVPSLEKKSCCQTTVATDIAKDPVPIAAVTSQADAGKEQQQVASELSMKGTQTNHIPNSSSRTRTKVQMQGVAVGRAVDLTTLKGYEDLIDELENVFEIKGELREMNKWSIVFTDDEYDMMLVGDDPWPEFCKMVKRIFIYSSEEVKTMSSSSKLVSPPSLDSLDSERKTES

>CmARF26

MDSVIEPMRNHEKYLDSQLWHACAGGLIELPTIDSKVVYFPQGHAEHAQENVDFGVAQIPSLIPCRVSGIRHMADSETDEVFANIRLIPLRNNEFSLDDDDELLGHNDIRTREKPASFAKTLTQSDANNGGGFSVPRYCAETMFPRLDYSAEPPVQTILAKDVHGEIWKFRHIYRGTPRRHLLTTGWSNFVNQKKLVAGDSIVFLRAETGDLFIGVRRAKRGNGYGIDYETGWDPTNSASPLVGYSDFMRDNEGRLVRRISNGNSSGRVTVESVIEAAMLAASGKPFEIVYYPCTGTPEFVVKASCSLVSCDEIQDAFRNQGFVSDKLVADPVRWPDSPWRMLQVAWDEPDLLQNVKSINPWLVEVVVNMPAIHVSPFSPPRKKQRFHPAETAVFGHLPMPSFSTNFFETTSSLQSVTGNNIPAGIQGARHTQFGLSSPDFQLSKLNLGPCSAGFKHLDEATPLPGTPGENMFGGMKSPDNSHWLTMGNNTESSKDSKETKPDHIILFGQLILPNQLISNSCSADTTNASYENQGKSSNPSDGSGLSSQQNGSLENSSEGGSTSYNGHEKTGFSLNTGQCKVFMEFEDIGRTLNLSLLRSYDELYGKLANMFGVGSSEMLNCVLYEDALGTTKQAGDEPFSTFLKTARKLTILTDSRSSDNSKT

>CmARF27

MKLSTSGFGQQDHEGGEKKCLNSELWHACAGPLVSLPTAGTRVVYFPQGHSEQVAATTNKEVDGHIPNYPNLPPQLICQLHNVTMHADVETDEVYAQMTLQPLTAQEQKDTFLPMELGIPSRQPTNYFCKTLTASDTSTHGGFSVPRRAAEKVFPPLDFSQQPPAQELIARDLHDVEWKFRHIFRGQPKRHLLTTGWSVFVSAKRLVAGDSVLFIWNEKNQLLLGIRRATRPQTVMPSSVLSSDSMHIGLLAAAAHAAATNSCFTVFYNPRASPSEFVIPLTKYVKAVFHTRVSVGMRFRMLFETEESSVRRYMGTITGISDLDPVRWPNSHWRSVKVGWDESAAGERQPRVSLWEIEPLTTFPMYPSLFPLRLKRPWHPGVSSVHDELILVYWLEYNRDDASNGLMWLRGGVGDQGLHSLNLQSMGSLPWLQQRLDSPMFGNDHNQQYQAMLAAGMPNLGGVDMLRQQIMHLQQPFQYIQQAGFHNSLLQQQQQLVQQSMPQNILKTPSQVMAENIPQHILQQTPQNQSEDIPNQQQRTYHETLQVQSNQFHQGENSNVPSPPFPRTELMDSNTNYSESMISRRNILASSCAEGTGNLSNIYRSGQSILTEQLPQQSPVSKNAHPQVDAHSNSMTFPPPFSGRDSILELGNCNSDSPSRTLFGVNIDSSGLLLPSNVPTYASPSIGPDSSSMPMGDSGFQNSMYSCVQDSSELLHSSGQVDPLNPTRTFVKVYKSGSVGRSLDISRFSSYQELREELAQMFGIEGQLVDPHRSGWQLVFVDRENDVLLLGDDPWEAFVNNVWYIKILSPQDFQKLGVQAIESFNPVGGQRLTSGGNEAENVSGLPSVGSLEY

>CmARF21

MASSEVSINPNSASVPFNDHADSTKLTSDPPNALSARDADFALFTELWNACAGPLVSVPRENDRVFYFPQGHIEQVEASTSQVADQQMPVYDLPSKILCRVINVHLKAEPDTDEVFAQITLVPEANQDEHAVDKEPPPPPPRRFHVHSFCKTLTASDTSTHGGFSVLRRHADECLPPLVGTSVFPGQPRRHLLQSGWSVFVSSKRLVAGDAFIFLRGENGELRVGVRRAMRQHGNVPSSVISSHSMHLGVLATAWHAISTGTMFTVYYKPRTSPSEFIVPYDQYMESIKKSYTIGMRFKMRFEGEEAPEQRFTGTIIGCEDADPKRWKDSKWRCLKVRWDETSTISRPEKVSPWKIEPALAPPALNPLPMTRPKRPRSNMVPTSPDSSVLTREGSSRVTVDPSPVSAFTRVLQGQEFSTLRGNFIDGNDPDAAEKSVMWPPSLDDEKIDVVSTSKKHGADCWITPGRSEPTYADLLSGFGTNLDSSHGVRAGMGDPAVVTANSIRKHVMDQDGKFNFLGGSSWSVLPSGLSLNLVDSSQKGHIQAGDLSYQVRGNATFNGFGDHSVAHCHRTEQPHGNWLMAPLSSHFDYPVQSTELMSMPMVFQNQDIMKPKDGNCKLFGISLIKNPAIPDSAGLNRNMNGADVTHLNIHQIHSNESDLKSEPSRGSMLADKSLAINDADKLQQTCTQNLKDAHCKSQGTSARSCTKVQKQGIALGRSVDLSRFNNYDELVAELDQLFEFGGELLAPKKNWLIVYTDDEGDMMLVGDDPWQEFCGMVRKIFIYTREEVQKMNRGSLNLEGDENPSVEGKEAKEIEGQAVPSISAPESFWCGTEGVEQSNAARPEGCGL

>CmARF20

MSSVEDKLKTPMGLVNNTPQTNLLDEMKLLKQMQDQTGARKAINSELWHACAGPLVSLPHVGSLVYYFPQGHSEQVAVSTKRTATSQIPNYPNLPSQLMCQVQSITLHADKDSDEIYAQMSLQPVNSERDVFLVPDFGLRPSKHPNEFFCKTLTASDTSTHGGFSVPRRAAEKLFPPLDYTMQPPTQELVVRDLHDNTWTFRHIYRGQPKRHLLTTGWSLFVGSKRLRAGDSVLFIRDEKSQLLIGVRRANRQQTTLPSSVLSADSMHIGVLAAAAHAAANRSPFTIFYNPRACPSEFVIPLAKYRKCVFGTQLSAGMRFGMMFETEESGKRRYMGTIVGISDLDPLRWPGSKWRNLQVEWDEPGCCDKQNRVSSWEIETPESLFIFPSLTSGLKRPLHGGFLGETDWESLVKRPMLRVPENIRGDLSYAAPNLCSEPLMKMLLRPQLVSLHGATLHQDSANNLVKIQDMKDMQPETNSKMKQLIPTETASPGNQNPTQSDPINPNSSPKESLPGKVHTSAAAESEPPTGADDVSTNQSNTLPPVGDCVGEKPTAKEVNMQTLVNQLSFMNQNQIPMQSWPLQPQLESLIQHPQPIDMPQPEFTLDGDGCLINPSCLPLPGVMISPGNLSMLALQESSTVFPEALNFPLPSTGQDMWDPLNNIRFSSNLNCMGNANVMRAVSDESNNQSGIYSCSNLEISNGGNTLVDPAVSSTILDDYCTLKDADFPHPSDCLVGNFSSSQDVQSQITSAASLGDSQAFSRQDFHDNSGGTSSCNVDFDEGSLLHNGSWNQVVPPMRTYTKVQKAGSVGRSIDVTSFKNYDELCSAIECMFGLEGLLNDPRGSGWKLVYVDYENDVLLIGDDPWEEFVSCVRCIRILSPSEVKQMSEEGMKLLNSAMMQGINCSMPEGGRG

>CmARF07

MRLSSAGFSPQPPEGERRVLNSELWHACAGPLVSLPAVGSRVVYFPQGHSEQVAISTNREVDAHIPSYPSLPPQLICQLHNVTMHADVETDEVYAQMTLQPLSAQEQKEPCLPAELGAPSKQPTNYFCKTLTASDTSTHGGFSVPRRAAEKVFPPLDFSQQPPAQELIARDLHENEWKFRHIFRGQPKRHLLTTGWSVFVSAKRLVAGDSVIFIWNEKNQLLLGIRRANRPQTVMPSSVLSSDSMHLGLLAAAAHAAATNSRFTIFYNPRACPSEFIIPLAKYVKAVYHTRVSVGMRFRMLFETEESSVRRYMGTITGISDLDSTRWPNSHWRSVKVGWDESTAGERQPRVSLWEIEPLTTFPLYPSPFSLRLKRPWPTGFPSFHGLKEDDLGLNSQLMWLRGDGLDRGIQAMNFPGIGVAPWMQPRLDASMVGMQPEIYQAMAAAALQEMRTVDPAKVQAASLLQFQQTQTLPNRPTTFMPPQILQQPQPTFIQGDDNQRLSHSQAQTLPTVLQQEIKHQTFNNQQQQQQQQVFDHQQIPSTITTMSQFSSASQSQAQSLQTTPPLCRQQSFSDSNPNHVTSPIISPLHSLLGSFPQDESSQLLNLPRTNPMVHSSTWPSKRAAVDPLLSSVNSQFVLPQGENMGTTQTNISQNAISLPPFPGRECSLDQGNTDPQSNLLFGVNIESSSLLMQSGMPNLRGICSDSDSTAIPFSSNYVNTAASNFSANPTGTPSNCIEDSGFLQSPENTGQVNPSTRTFVKVYKSGSLGRSLDISKFSSYHQLRSELAHMFGLEGELEDPVRSGWQLVFVDRENDVLLLGDDPWPEFVNSVWCIKILSPQEVQEMGKQGLELLKSVPIQRLSNGSCDNYANRQESSRNLSTGITSVGSLEY

>CmARF06

MGALIDLNTTEEDESPSSAASSVSSSSASALTSSPSPSLTSSICLELWHACAGPLTSLPKKGNLVVYLPQGHLEKMQEFPATPSDLPPHILCRVIDVQLHAEAGSDEVYAQVSLLPENEQMEHKMQEEMSNDIEEEDVEEGEKTTTPHMFCKTLTASDTSTHGGFSVPRRAAEDCFPPLDYNQQRPSQELVAKDLLGLKWKFRHIYRGQPRRHLLTTGWSAFVNKKRLVSGDAVLFLRGNDGELRLGIRRAAQLKSGSTFSNICSQKLNSSSIMDVVNAISSKSSFSVCYNPRATSSQFVLPFHKFLKSMNRSFSVGMRFRLSFETEDAADLRHTGHITGIGDVDPIGWPGSRWRSLMVRWDDGEINRHGRVSPWEIEPSGSVSMSSNLVPPGLKRSRIGLSSTKLEFPVPHGIGATDFGESLRFHKVLQGQEILGYNTPTDCDDNTRYHPPEKRRLLPDLHGSGIALMRNGPRNRLINSETSSRGFVFDESFQFQKVLQGQEIFPGPFYGRTTATNEVKESGGCGPVDGFRLSTTRDGWPTPMQCENFHTRSSIPSAQVSSPSSVFMFQQSMVPVLSFDSHNRGSMNNSTSHNSGTAFMTDNSMCPTSLGELNLLGLSHPSTTTASAFSGSKDLSSTTKAGCRLFGFSLTEEKNVGNTDKGSPPTTPIHAGTTIGAQCPLKSPLMSKVVGSNCTKVSNISARAVEAIYFA

>CmARF05

MITFMDSKEKSKEMDKCLDPQLWHACAGGMVQMPPVNARVFYFPQGHAEHACASPVDFRTCPRLPSYTLCRVSAIKFLADPDTDEVFAKLRLIPINGSELDFDDGIGRLTGSVQDKPTSFAKTLTQSDANNGGGFSVPRYCAETIFPPLDYSADPPVQTILAKDVHGETWKFRHIYRGTPRRHLLTTGWSSFVNHKKLVAGDSIVFLRAENGDLCVGIRRAKRGAGDGPDLSCGWNGAVPYGAFSANESLMGKGKVKAESVIEAATLAANGQPFEIVFYPRASTPEFCVKAALVKAALQIRWCSGMRFKMAFETEDSSRISWFMGTVNSVQVADPLRWPESPWRLLLVTWDEPDLLQNVKRVSPWLVESVSNMSPIHIAPFSSPRKKLRHPQHPDFPLDNQPPMPLFSSYLHGSGSPFGCPPDNNSAGMQGARHAHFGLSLSDFHLSKLHSGLFPIGYRSPDPAAESATLSCNAMTEKPSMSENVSCLLTMAHSTQASKKCDGMKTPQLILFGRPILTELHMSQTYSGDTVSSVGTGNSSPDGNGDRTEKRADGSGSSLHQQRPLEGSSCENFQCYKDDRQEVEPNVDPGHCKVFMESEDVGRTLDLSSLGSYEELYTQLGNMFEIDNSETLNHVLYRDVSGAVKHVGDEQFSDFIKTARRLTIL

>CmARF30

MRLSAAGFSPQPPEVHARFMSFCCLGVLFCERRVLNSELWHACAGPLVSLPAVGSRVVYFPQGHSEQVAASTNKEVDTQIPNYPSLPPQLICQLHNLTMHADADTDEVYAQMTLQPLSAQELKEAYLPAELGTPSRQPTNYFCKTLTASDTSTHGGFSVPRRAAEKVFPPLDFSMQPPAQELIARDLHDNEWKFRHIFRGGNEKNQLLLGIRRASRPQTVMPSSVLSSDSMHLGLLAAAAHAAATNSRFTIFFNPRCEASPSEFVIPLAKYVKAVYHTRVSVGMRFRMLFETEESSVRRYMGTITGISDLDPVRWQNSHWRSVKVSWLGRVNSRRAAAKSVLVGNRTINNISNVSITVSTSTEATMANWTAFFYGYSCLTSLYVFLLGIKDSDLGMNSPFMWLRGDNSDRGIQCLNFQGNGVSPWMHPRLDPSMVGMQSDVYQAMAAAALQEMRAIDYSKLAPASMLQFQQPQGLPCQPSTLMQPQMLHQSQSQHAFLQSVQEHQQHSQSQTQTQSHHLPPQLQPEPSFNNQQPQHQQQPRQPQPQPPPQPQPLVDHQPIPCTIPAISQFASCSQSQSPSLQTVPSLCQQPSFSDSNGNPVTSPTVSPFHSLAGSFVQDESSQLLNIQRANSVIPSAGWPSKRAAIDPLSTGASQYFLPQVESGMSQNTVALPPFPGRECAIGDRDEGADPENHVLFGVNIESSSLLMQNGTSNLRGVGNDNVSTTLPFSSNYMSSSGTDFSVNPTTNCIDESGFLQPHENVGQVNQPNGTFVKVHKSGTYSRSLDITKFNNYLELRSELARMFGLEGELEDPLRSGWQLVFVDRENDVLLLGDGPWPEFVNSVWCIKILSPDEVQEMGKRGLELLNSMPIQRHSNNTCDDYGSRQDSRNLISGITSVGPFDY

>CmARF13

MDPKLWRAFAGDLAHLHTVGSEVYYFVQGHVEQATYAPKLSPAVLSNPVSKCLVTGVGLDADALTDEVLIKINLHPIRPGEGRSEVVSRLGCSEVNVISKFAKVLTSSDANNGGGFSVPRYCAVSIFPPLNFQADPPVQTLAITDVHGVVWKFRHIYRGTPRRHLLTTGWSKFVNHKKLIAGDSVVFAKNSRGEMFVGIRRARSNRPFTGSECSRVEENPSGDGDPRNFSRRTIGRVPPEVVATAAELAAQFKPFEVVFYPRTGLSQFVVPVEIVNNSMKYQWYPGIRVKLPTETEDTLRTQWHQGTIISVSIPEHDPWKGSPWRLLEITWEETDAPPNGKYVCPWEVELAGPAPPIQPSLHIAKRPRGHSKSGQLNGEAEPFSPMMRVGDSSMEQFNQALLSFNSFPAGMQGARQNFLCESGLFDNPYKETTNAEPTTQMVSQMVSQVVSTDLHIGSAQSDTLSPDSQASVLSFATESADNQLSNSTEAGVTSFQLFGQIIHLNPPAENGANTDDVDMTSNQSD

>CmARF09

MSFIQPSVALLNMYLVFLFSGSSSDALYRELWHACAGPLVTLPRQDERLEASMNQGLEQQMPSFNLASKILCKVVNVVLRAEPDTDEVYAQITLLPESNQSEVTTPDPALPEPTRCNVHSFCKTLTASDTSTHGGFSVLRRHADDCLPPLDMSQQPPWQELVATDLHGNQWHFRHIFRGQPRRHLLTTGWSVFVSSKKLVAGDAFIFLRGENGELRVGVRRLMRQLNNMPSSVISSHSMHLGVLATASHAVATGTLFSVFYKPRTSRSTFLVSLNKYLEAQNHKLSVGMRFKMRFEGEEVPERSFSGTIVGLGDNASPGWANSDWRSLKVQWDEPSSILRPDRVSAWELEPLVASNPLSSQPTQRNKRPRPTVLPSPTADAAVLGGWKPTVESSAFSFTEPPQRGRDVYSSPKFSTAASNSLGFNGNSSLGAVSSNNYWSNTNRVENIIDTSSHGANREPIEKKQNTRNGCRLFGIQLLGNSDVGEASPDTTPKMVSEDRSVPPVDTEFDQNSERSNVHRLNIPSVSCDADKSCLISPLESQSRQIRSCTKVHMQGIAVGRAVDLTRFNQYDDLLGKLEEMFDIEGELCGSVKKWQVVYTDDEDDMMMVGDDPWKYLNHVWIRNLGFMYGFLNDCSEFVSMVRKIFIYTTEEVKRLSPKIKLPLGGETKLSKPDSDTTANHTEDQSSIVGGRSNGICNKAFVYFHLETLKIPTTTKLAAGAATYMKGVGESVFVWVVDDNGGRKKLKTSSMLPM

>CmARF28

MACNGGDSYTQSCVSTQGSGRNDLYTELWRACAGPLVDVPRVDERVFYFPQGHMEQLEASTNLELNKRIPLCNLSSKILCRVIHIELLADHESDEVYAQITLMPESNQNEPNSLDPCPPEPARPVVHSFCKVLTASDTSTHGGFSVLRKHATECLPPLDMTQATPTQDLVAKDLHGYEWRFKHIFRGQPRRHLLTTGWSTFVTSKRLSAGDSFVFLRGDNGELHVGVRRRARQQSSMPSSVISSQSMHLGVLATASHAVTTQTRFVVYYKPRTCQFIISLNKYLEAVNNKFSVGMRFNMSFEGEDSPERRFSGTIIGATDISPHWPNSSWRSLRVQWDEQTSILRPDRVSPWDIEPLTSSAVTGLSQPISKNKRPRQSTPAHDGADLPKPALWDSGLAESHDGKQSSNAAESRKGENNELCHHRETDAISNRTCVSRTQADGTWLSSIQSNSHKHPVNDMAQDYKTVPGCGWSFLSGPSTSHLVKLSDEQILDSTENGRKGETVASCRLFGIDLNHLAKLPTEKASYQPSSVSSDTDGRSSTLSVAQSDPKSDNLEVSVERKSEPLQASLKETQSNQSSSANTRSRTKVHMHGMAVGRAVDLAILEGYDQLIDELEKMFDVRGQLCARDKWEIVYTDDEGDMMLVGDDPWEEFCNMVRRIFICSREQVKKMSSGSKQLTSIEVEGAAIIPDSPAV

>CmARF32

MKEAEKSLDPQLWHACAGGMVQMPAINSKVFYFPQGHAEHAQATVDFTSSLRIPPLILCRVLAVKFLADPETDEVFANVSLVPLANNDLNFEEEGGFGGNGSDNNMEKPASFAKTLTQSDANNGGGFSVPRYCAETIFPRLDYTADPPVQTVIAKDVHGEVWKFRHIYRGTPRRHLLTTGWSSFVNQKKLVAGDSIVFLRSKNGDLCVGIRRAKRAIGCAVDHPYGWNPGGGNCFPPCGGLTMFLRDEDNNKLSRKGSVSSSGGGGNLRGKSKIRPESVIEAAALAASGQPFEVVYYPRASTPEFCVKASSVRAAMRIQWCSGMRFKMPFETEDSSRISWFMGTTSSVQVADPIRWPNSPWRLLQVTWDEPDLLQNVKRVSPWLVELVSNMPVIQLSPFSSPRKKFRLPQHPDVPLDSQFPLSSSSSSFSSNTLRPSSPMCCLSDNTSVGIQGARHTHFGISLSDFHLNNKLQLGLVPSSFQQLHFHSRFSNTDHTRSSSALLNGEKTGPKLERSDSVKKKHQFVLFGQPILTEQQICCSSSSDIHQVSPNKSDTKLGLDIGHCKVFMESEDVGRTLNLSVIGSYEELYRRLANMFGMEKPDILSHVLYQDATGAVKQAGDKPFSDFIKTARRLTILTDESGSNKMGRRTLMDGVLSGENARLDASNKTGPLSILA

>CmARF31

MEIDLNQTASEVGKNAYCHGNCEEGRCNCCLSSSTSSCSSNSSSTPASSSTYLELWHACAGPLTSLPKKGNAVVYFPQGHLEQIASASPFSPMNMATFDLQPQILCKVINVHLLANKENDEVYTQLTLLPLPELLGTGVEGKELEELALNRAADGDGSGGSPTRSTPHMFCKTLTASDTSTHGGFSVPRRAAEDCFPPLDYTQLRPSQELIAKDLHGVEWRFKHIYRGQPRRHLLTTGWSIFVSQKNLISGDAVLFLRGENGELRLGIRRAVRPRNGLPDSIVGNQNSCANDLTRVVKAVSTKSAFDVFYNPRAYHAQFVVSCQKYVKSINNPVNVGTRFKMRFEMDDSPERRFNGVVVGIGDMDPFRWSNSKWRCLTVRWDKDSDHQERVSPWEIDPSVSLPPLSVQSSPRLKKLRTSLQEAPPNNAFNGRGGFMDFEDSVRSSKVLQGQENVGMVSPFYGCDTAKRSLEFEVRSSAQQNQASGGVEKLNIGDYVTSFTGFMESDRFLKVLQGQEICSLRPQTRKPEPSLGVWGKFNLSDNSFNPFQSPNSSFYHMASNGARNMYYPHRDIYSTGQAAMMSSNDINFPRESALSNPSAKMERANSTPPTLGSNMRNSKDENVNENRTGCKLFGFSLTTETATNMQSSGKRSCTKVHKQGSLVGRAIDLSRLNGYTDLLSELERLFSMEGLLKDPDKGWRVLYTDNENDVMVVGDYPWHDFCDAVSKIHIYTEEEVEKMTNGVISDDTQSCLDQAALCMEASKSSSVGQPDSSPTVVRV

>CmARF14

MKTPANGAAAMPNSSEGGLEKKIINPELWQACAGPLANLPPAGYHVVYFPQGHSEQVAASLKKDVDGQVPNYPNLPSKLLCLLHNVTLHADPETDEVYAQMTLLPVPSFDKDALLRSDLALKSNKPQPEFFCKTLTASDTSTHGGFSVPRRAAEKIFPPLDFSMQPPAQELVAKDLHDNVWTFRHIYRGQPKRHLLTTGWSLFVSGKRLLAGDSVLFIRDEKQQLLLGIRRANRQPTNLSSSVLSTDSMHIGILAAAAHAAANNSPFTVFYNPRASPSEFVVPLAKYYKAVSANQISLGMRFRMMFETEESGTRRYMGTITGISDLDSVRWKGSQWRNLQVGWDESTGGERRNRVSIWEIEPVIAPFFICPPPFLRSKRPRQPGMPDDDSSDLDGICKRTMFGDDFCMKDPQGYPGLNLVQWMNMQNPSLSNAMQPSYMHSFSGSMLPNLGGVDISRQIGLSNTQIPQSNNIQFNAQRLLSQAQQLDQLPKLPSSMNSLGSVVQPSQQLDDMSQQTRSNLINQNAVSSQIQAQIMQQQQPHTNGIVLQQNPQLQRSLPQNQPQTMNQSPLPEPMIHQLQMSDNQVPMQMLQKLQQQQQQQSLLAQQSVVLQPGQLPEQLRQSVDASFSRSMSSNQMLDIPQSTPAAVPPSNVSPQQAAKSNGTTNNRFSNQHLQPKLPQLQQPAPSTVLSDMSRPVGLPPTQTYNQLSAATSSVITGAAAAGQSGVTDDIPSCSTSPSTNNGPSLIQPVNNGRVHRSTGLVEDVAQSSATIFSTNTLTAVKPSLNISKNQSHGIFAQQTFLSGVVSQTDFLDTSSSTTSACLSQNEAQLQQNNVMSFNSQPMLFRDSTQDLEVPTDLHNVPYGTNVDGPLVAQLNSDPLLNKGIGELGKDFSNNFSSGAMLTTYDAQKDPQQEISSSIVSQSFGIPDMTFNSMDSAMNDNAFLNRNQWAPPPPFQRMRTYTKVYKRGAVGRSIDITSYSGYDELKQDLARRFGIEGQLEDRQKIGWKLVYVDHENDVLLVGDDPWEDFVNCVRSIKILSPQEVQQMSLDGDFGNGVLLNQACSSSDVGSSKLSKAMISQQWRRYSQPQHSAMGFSHCLVKFSWTSKLRQLQKQS

>CmARF25

MRLSAAGFSPQPPEGERRVLNSELWHACAGPLVSLPAVGTRVVYFPQGHSEQVAASTNKEVDAQIPNYPSLPPQLICQLHNLTMHADAETDEVYAQMTLQPLSAQELKEAYLPAELGSPSRQPTNYFCKTLTASDTSTHGGFSVPRRAAEKVFPPLFMISSDDIGAMQDFSMQPPAQELIARDLHDNEWKFRHIFRVGLGEMESWLPCFNCLDVLDMFVAGQPKRHLLTTGWSVFVSAKRLVAGDAVLFIWNEKNQLLLGIRRASRPQTVMPSSVLSSDSMHLGLLAAAAHAAATNSRFTIFFNPRASPSEFVIPLTKYVKAVYHTRVSVGMRFRMLFETEESSVRRLLSASLVEYRRFDNWDSMCIHIFVIFLRYMGTITGISDLDPVRWQNSHWRSVKVGWDESTAGERQPRVSLWEIEPLTTFPMYPSPFPLRLKRPWPTGLPSFGKLAPSLQHQQCVNMTAFYFLFFLTMKFILMLTPLYVSLLGIKDSDLGMNSPFMWLRGDNCLNFQGNGVSPWMQPRLDTSMMAMQPDMYQAMATAALQEMRAIDYSKIAPASVLQFQQSQGLPCQSSSLMQPQMMHQSQPQQAFLQSIQENQQHHLQPQLQPQQFNNQQQQPRQPQPPQLDHQQIPCTIPAAISQFASCSQSQLPSLQTIPSLCQQPSFSDSNCNPVTTTVSPLHSLAGSSSVQDESSQLLNLQRANSIIPSAGWPAKRAAIDPLTTGASQYFLPQGEILGTSQSSIPQNTVALPPFPGRECSNDDRDDGSDPQNHVLFGVNIESSSLLMQNGASHLRGVGNDSVSTTLPFSSNYMSTAGTDFSVNPTMTSSNCIDESGFLQSHENVGQVNHPPNGTFVKYMLNPSGHTFFVQVHKSGTYSRSLDITKFNSYPELRSELACMFGLEGELEDPLRSGWQLVFVDRENDVLLLGDGPWPEFVSSVWCIKILSPEEVHEMGKRGLELLNSVPIQRLSNSTCDDYGSRQDSRNLVSGITSVGPLDY

>CmARF16

MNKGLSSSKAFGCIDFGASNLGVNSSLVLLDLTEMRLSAAGFSPQPPEGEKRVLNSELWHACAGPLVSLPAVGSRAVYFPQGHSEQRINNSTHFSSKFKICLLSTCIWSSFLFLVIAFARCLAVAISTNKEVDAHIPSYPSLPPQLICQLHNVTMHADIETDEVYAQMTLQPLSAQEQKEPYLPAELGAPSKQPTNYFCKTLTASDTSTHGGFSVPRRAAEKDFTQQPPAQELMARDLHDNEWKFRHIFRGEFCEVNVLKTASTPRLAAVFMSNYIFDAGQPKRHLLTTGWSVFVSAKRLVAGDSVIFIWFVFMPFLTRYLFIQIYIIFAMLGFLRNEKNQLLLGIRRASRPQTMMPSSVLSSDSMHLGLLAAAAHAAATNSRFTIFYNPRASPSEFIIPLAKYVKAVYHTRVSVGMRFRMLFETEESSVRRYMGTITGISDLDPARWPNSHWRSVKVGWDESTAGERQPRVSLWEIEPLTTFPMYPSPFPLRLKRPWPTGLPSFHGLKEDDLGLNSQLMWLRGDALDRGIQPLNLHGIGVAPWMQPRLDASMVGLQPEIYQAMAAAALQEMRTVDPAKAQAASLLQFQQTQNLPNRPATFMSPQMLQQPPQPPQTQPHQTFLQNDHEIQHLPHSQAKTQPTVLRQEMKQQTFNNEQQQQQQQQQPQQQVFDHQQIPSSISTMTQFGSVSQSLQTIPSFCRQQSFSDSNGIHMTNPIISPLHSLLGSFPQDESSQLLNLPRTHPMIHSSAWPSKRAAIDPLISSGNSQFVYLQEENMGTAQANISQNVSLPPFPGRECSLDQRNGDPQSNLLFGVNIEPSSLLMQNGMPNLRGICSDSDSTAIPFSSNYVNTAGTNFSANPAGTPSNCIEDSGILPNFVKVYKSGTFGRSLDISKFSSYHQLRSELAHKFNLEGELEDPLRSGWQLVFVDRENDILLLGDDPWQEFVNSVWCIKILSPQEVQEMGRVDSYADRQESSRNLSSGITSVGSLEY

>CmARF17

MAALIDLNTTEEDDAPSSAASSASSSSASALTSSPSPSLTSSICLELWHACAGPLTSLPKKGSLVVYFPQGHLEQMQEFPATAAYDLPPHILCRVIDVQLHAEAGSDEVYAQVSLFPENEPIEHKMQEEMTNDSEGEDFEGSEKTTTPHMFCKTLTASDTSTHGGFSVPRRAAEDCFPPLDYSQQRPSQELVAKDLLGIKWKFRHIYRGMKCKSQIFGALLTLAVCFPYYVLLSSLSGQPRRHLLTTGWSAFVNKKRLVSGDAVLFLRGNDGELRLGIRRAAQLKTGSAFSNICSQQLNSSSIMDVVNAISSKTSFSVYYNPRATSSQFVLPFHKFLKSINHSFSAGMRFSLSFETDDAADRRVLESSITFLSYSYRRCTGRITGVGDVDPIRWPGSRWRSLVVRWDNVETKRHGRVSPWEIEPSGSVSVASNMVPPGLKRTRIGLSPTELEFPAPNGIGASDFGESLRFQKVLQGQEILGYSTPIDGDNNIRHPPEKRRSFPGLHGSGIAIMRNGPRIPVTNSETSSRGFMFDESFQFHKVLQGQEIYPSPFFGIATATNEVKASGGYGPVDSVPLSRSKDGWPITMQSENFLTRSSIPSVQVSSPSSVFMFQQSMVPVQSFNSYNRGNFTEQRTMNKSTSHNSGTAFMTTSLGVQKQLALSHPSTTEPAFTGNKAGCRLFGFSLTEGKNVGNTDNASPATTPINAGTASVLSSNSGLRCPLKSPLMSKVVGSNCTEGAVQYHFANCSTYY

>CmARF08

MGFPSFIFFFHNPILGLISFSVNGDFQGSGRDGLYVELWKASAGPLVEVPRVHEHVFYFPQGHMEQLEASTNQELNQKLPSFNLPSKILCHVVDVRLLAEQETDEVYAQISLIPEGNQEEPATPGPSPGECRKSKVHSFCKVLTASDTSTHGGFSVLRKHATECLPPLDMTQQTPTQELIAKDLHGYEWRFKHIFRGQPRRHLLTTGWSTFVTSKRLVAGDSFVFLRGENGELRVGVRRLSRQQSTMPSSVISSHSMHLGVLATASHAAATLTRFVVYYKPRASQFLVSLSKYKEAMNTKFLVGMRFKMRFEGEDSPERRFSGTIIGVDDISPYWPNSQWRSLRVQWDELASIQRPERVSPWEIEPFVAPASQSIPQSISVKNKRPRPPLDIPDSDNSTVTTLRHPVSTQSYDDKTQLSVPAGWHYKQTDISSNGNSVSRTHNEGSWLTVPNGSVSQHRLKDSTEDHKSNSVWSAVFPGVPTAHSTCPTPRTSNPMSDQINEFGEEGRKTEVAPSCRLFGIELIDHSKRPVPPERTADQSNSAANEVTESHVNTLLSSNAEQQGSNLPKASSKERKLGLLQALPKEIQHKHNASTNCRSRTKVQMQGMAVGRAVDLTTLVGYDQLIDEMEKMFDIKGELRPRDKWEIVFTDDEGDTMLMGDYPWQEFCNMVRRIYIWSSQDVKTSSGRRLTMSAIEFDGTAITSESADS

>CmARF24

MAFDFPESSLCHHHLHPLDSFAIHFSFSFFFTTQTGSLYSLWGSSALPRLLRFFSFREPCCCFQRRRDWHGCVLAFTELVIRFPLDLWVISYSMKAPPNGFVPNSGEGERKNINSELWHACAGPLVSLPPVGSLVVYFPQGHSEQVAASMNKETDFIPNYPNLPSKLVCMLHNVILHADPETDEVYAQMTLQPVNKYEKEALLASDIGLKQNRQPAEFFCKTLTASDTSTHGGFSVPRRAAEKIFPPLDYSMQPPAQELVARDLHDNSWTFRHIYRGQPKRHLLTTGWSVFVSTKRVFAGDSVLFIRDEKSQLLLGIRHANRQQPALSSSVISSDSMHIGILASAAHAAANNSPFTIFYNPRASPSEFVIPLAKYTKAMYTQVSLGMRFRMMFETEESGVRRYMGTITGISDMDSVRWKNSQWRNLQVGWDESAAGERPSRVSIWEVEPVVTPFYICPPPFFRPKFPKQPGMPDDESDIENAFKRAMPWFGDDFGMKDTPSSIFPGLSLVQWMSMQHNSQFPAAQSGILPSMVAPSALHGTLTNDDPSKLLCFQAPVMSSSNLQFSKANQQNQVGQLPATSWSQQQQLQQLPPQPELRQQQQQPKQSQQQQQTSQSALPNNGVGGANHLSNQSLQQPLVYSQLQQQQQQLLAGNVQSHQTSQPSNKNSIETTSLLQETQFQPQIDQQPTLVSKHHQQTQFQQAPLQLLQQSLSQRTQQQPQVQQFSQPIIPSEQQLQWQLLQKLQQQQQQQQQQQPLVSPASPLFPPQMIQPHPVHQQNQQLPPLPLSNQPQFNTSGGSFQTEKHNSNGFSSLGLMQSQQVPITQSHNQFKPTTAIRGYSGLTDGDAPSCSTSPSTNNCQVSVSNLLNKNQQGAATLGGDLVVEPATNLPQELPSKPDLRNKPEFPNSKGLDQLKYKGTVPDQVEASSSGTSYCLDAGTIQQTFPLPTCLDNDVQSHPQTNIPFSNSIDGWAPDTLLSRGYDSQKDLQNLLSNYEGGVPRDIETELSTAAIRSQSFGVPNLPFKPGCSNDVNVNEAGALSSGLWANHSQRMRTYTKVQKRGSVGRCIDVTRYKGYDELRHDLARMFGIEGQLEDPQRTDWKLVYVDHENDILLVGDDPWEEFVSCVQSIKILSSSEVQQMSLNGNLGHIQAPNQACSGTDSGNAWRGQYDDNSAASFN

>CmARF04

MKTPANGVASSAAAPNSCEGGLEKKIINPELWQACAGPLVNLPPAGYHVVYFPQGHSEQVAASLKKDVDAQIPNYPNLPSKLLCLLHNVTLHADPETDEVYAQMTLQPVPSFDKDALLRSDLALKSNKPQPEFFCKTLTASDTSTHGGFSVPRRAADKIFPPLDFSMQPPAQELVAKDLHDTVWTFRHIYRGQPKRHLLTTGWSLFVSGKRLLAGDSVLFIRDEKQQLLLGIRRANRQPTTLSSSVLSSDSMHIGILAAAAHAAANNSPFTVFYNPRASPSEFVIPLAKYYKAVSANQISLGMRFRMMFETEESGTRRYMGTITGISDLDPVRWKGSQWRNLQVGWDESTGGERRNRVSIWEIEPVIAPFFICPPPFFRSKRPRQPGMPDDESSDLDSIFKRTMFGDDFCMKDPQGYPGLNLVQWMNMQNPSLSNMQPNYMHSFSGSMLPNLGGVDISRQLGMSNAQNPQSNNIQFNAQRLLPQAQQLDQLPKIPSTMNSMASVVQPPQQFDDISRQTRQNVVSSQIQSQIMQQPHGNGILQQQTSLQNQQLQRSLPQNMLMQQHQQILGQNQQQNMNQPPIPDLSNHPLQMSDNQIQMQMLQKFQQQQQSLLAQQSVLQPAQLAQLPEQQSFSISMSTNQTIDVPHSAPAAGPPPSAQQAPKSHSSNQHLQPKFPQLQQQPHDMSRPMGHNNQFSAATSSAITGAAGAGQSGITDDIPSCSTSPSTNNCPSLVQPVTNGRLHRTTGLVDDVAQSAATIFSTNTLDSISPNANLVKNIPHKAAVKPSLNISKNQSHGVLTQQTFLTGAVPQTEFLDTSSSTTSACLSQNDAQLQQNNIMSFNSQPMLFRDGSQDLEPPTDLHNIPYGTNVDGQLVAHLNSDPLMNKGIGGLGKDFSNNFSSGDMLATYDAQKDPQQEISSSIVSQSFGIPDMTFNSMDSTINDSSFLNRNQWAPPPPFQRMRTFTKVYKRGAVGRSIDITRYSGYDELKQDLARRFGIEGQLEDRQKIGWKLVYVDHENDVLLVGDDPWEDFVNCVRSIKILSPQEVQQMSLDGDFGNGVLPNQACSSSDAQSSSLRLRFPVSNGGDIIGNQHSLSNSLVIVWLNFLETLSLRQL

>CmARF29

MANRGGGSFSSQGGGGDGLYTELWKASAGPLVEVPQVNDKVFYFPQGHMEQLEASTNHELNQKHPLFKLSSKILCRVVDVRLLAEQETDEVYAQITLMPDSNQEEPTNPDPSPPECRKPKVHSFCKVLTASDTSTHGGFSVLRKHATECLPPLDMTQQTPTQELVAKDLHGNEWRFKHIFRGQPRRHLLTTGWSTFVTSKRLVAGDSFVFLRGENGELRVGVRHLARQQTSMPSSVISSHSMHLGVLATASHAVLTLTRFIVYYKPRASQFIVSLNKYIEAMNRKFLVGMRFKMRFEGEESPERRFSGTIVGVDDISPHWPNSKWRSLRIQWDELASIPRPDRVSPWEIEPFVAPTSPSIPQSVSVKNKRLRPPLDIPDSDNSTVTTLRHPGSTQSHDDRTQLSGTAAEMKRFENHAMWNYKQTDVSSIGNSISRTPKEGSWLASPNRSVSQHRLQNLTDDRNSNYVWSTVFSGAPAAQSTCPAPHPSNPKSSDQVNDLGEKGRKTEVAPSCRLFGIDIIGHSKSPVPPEMAADQPISAPNEITDAEQNSDQPKASKERKLGLLQVPPKEIQHKQSSSTNSRSRTKVQMQGMAVGRAVDLTMLEGYGELIDELEKMFDIKGELHPRDKWEIVFTDDEGDTMLMGDYPWQEFCNMVRRIYIWSSQDVKMMSSVSKLTMSAMECDGTVITSKSADS

>CmARF22

MDRQLWQAFAGKSVHIHTVGSEVYYFVQGHVEQATYIPSLSQSVFSNPATKCLVTGTDFHADSLSDEVCIKLNLHPIRPGRDESVVVSRFGRCDDNGGERDKIESFAKILTSSDANNGGGFSVPRCCADSVFPPLNYQADPPVQNLSITDVHGVVWNFRHIYRGTPRRHLLTTGWSKFVNHKKLIAGDSVVFAKNLRGDMFVGIRRASTSITRAIAGGDCGRWNSQNGGARCSLEENCSGDGGTKVFSRRNIGKLPAEAVANAAELAAQFKPFELVYYPRAGRSEFVVQVEKVNESKNYQWYSGTRVKMAMETEYSKMTWYQGTVTSASVPEHGPWMGSPWRMLEVTWDEIDTLQSAKYVSPWEVELATPTPPIQPPLHPAKKFRGLPKSGMLNGEAELFSPLMNFVDSTMEQFNPSLLNLNSFPAGMQGARQKLFCESRLFNPCKEMTPLTCDESSMSELNAAKKMQMVSTDLLIGSVQFDTLSPDSQASVLSFATGTAENQNCCNSAKAGALINIYREAFLDGDQKTISKVEAKLKIFEREKDGLFQKVSNTSAEITSGKENYIRLQADFDNFRKRSEKERLTVKNNAQKEVIENLLPMIDSFEKARQQIVPQTDKEKKIDVSYQGIYKQFVEVLRSWRISAVAAVGRPFDPSLHEAVAREESQEIKEGIIIQELRRGFLLGERLLRPARVKVSKGPGKKNSPTTNSEKPTEHPATATARVDEH

>CpARF01

MANRGGGSFSPSNVSSQGSGRDGLYVELWKASAGPLVEVPRVHEHVFYFPQGHMEQLEASTNQELNQKLPSFNLPSKILCHVVDVRLLAEQETDEVYAQISLIPEGNQEEPATPGPSPGECRKSKVHSFCKVLTASDTSTHGGFSVLRKHATECLPPLDMTQQTPTQELIAKDLHGYEWRFKHIFRGQPRRHLLTTGWSTFVTSKRLVAGDSFVFLRGENGELRVGVRRLSRQQSTMPSSVISSHSMHLGVLATASHAAATLTRFVVYYKPRASQFLVSLSKYKEAMNTKFLVGMRFKMRFEGEDSPERRFSGTIIGVDDISPYWPNSQWRSLRVQWDELASIQRPERVSPWEIEPFVAPVSQSIPQSISVKNKRPRPPLDIPESDNSTVTTLRHPVSTQPHDDKTQLSVPAGWHYKQTDISSNGNSVSRTHNEGSWLTVPNGSVSQHRLKDSTEDHKSNSVWSAVFPGVPTAHSTCPTPRTSNPMSDQINEFGEEGRKTEVAPSCRLFGIELIDHSKRPVPPERTSDQSNCAANEVTESHVNTLLSSNAEQQGSNLPKASSKERKLGLLQALPKEIQHKHNASTNCRSRTKVQMQGMAVGRAVDLTTLVGYDQLIDEMEKMFDIKGELRPRDKWEIVFTDDEGDTMLMGDYPWQEFCNMVRRIYIWSSQDVKTSSGRRLTMSAIEFDGTAITSESADS

>CpARF02

MSFSAASNLPSGGPHSGSSSDALYRELWHACAGPLVTLPRQDERLEASMNQGLEQQMPSFNLASKILCKVVNVVLRAEPDTDEVYAQITLLPESNQSEVTTPDPALPEPTRCNVHSFCKTLTASDTSTHGGFSVLRRHADDCLPPLDMSQQPPWQELVATDLHGNQWHFRHIFRGMVYKVEQLLYLTPSQLFAFAIALLIYLEQLANSRFLNILPYDRTSRSTFLVSLNKYLEAQNHKLSVGMRFKMRFEGEEVPERSFSGTIVGLGDNASPGWANSDWRSLKVQWDEPSSILRPDRVSAWELEPLVASNPLSSQPTQRNKRPRPTVLPSPTADAAVLGGWKPTVESSAFSFTEPPQRGRDVYSSPKFSTAASNSLGFNGNSSLGAVSSNNYWSNTNRVENIIDTSSHGANREPIEKKQNTKNGCRLFGIQLLGNSDVGEASPDTTPKMVSVDRKLEEMFDIEGELCGSVKKWQVVYTDDEDDMMMVGDDPWNEFVSMVRKIFIYTTEEVKRLSPKIKLPLGGETKLSKPDSDTTANHTEDQSSIVGSDC

>CpARF03

MLSIESDWVKLDMKLSTSGFGQQDHEGGEKKCLNSELWHACAGPLVSLPTSGTRVVYFPQGHSEQVAATTNKEVDGHIPNYPNLPPQLICQLHNVTMHADVETDEVYAQMTLQPLTAQEQKDTFLPMELGIPSRQPTNYFCKTLTASDTSTHGGFSVPRRAAEKVFPPLDFSQQPPAQELIARDLHDVEWKFRHIFRGKSPYVRNEKNQLLLGIRRATRPQTVMPSSVLSSDSMHIGLLAAAAHAAATNSCFTVFYNPRASPSEFVIPLTKYVKAVFHTRVSVGMRFRMLFETEESSVRRYMGTITGISDLDPVRWSNSHWRSVKFSVSFSTVKVGWDESTAGERQPRVSLWEIEPLTTFPMYPSLFPLRLKRPWHPGVSSVHDNRDDASNGLMWLRGGVGEQALHSLNLQSAGSLPWLQQRLDSSMFGNDHNQQYQAMLAAGMPNLGGVDMLRQQIMHLQQPFQYAQQAGLHNSLLQLQQHQLVQQSMSQNILQAPSQVMAENLPQHILQQTLQNQPEDLPNQQQHTYHDTVQVQSNQFHQGGHSNVPSPTFPRSNLVDSNSNYPESISSRRNIIASSCAEGTGNLSNIYRSGQSILTEQLPQQSPVSKNAHSPVDAHPNSMSFPFSGRDSILELGNCNSDSPSPTLFGVNIDSSGLLLPSNVPTYASPSIAPDSTSMPLGDSGFQNSLYNCVQGSSELLHSSGQVDPSNPTQTFVKVYKTGSIGRSLDISRFSSYQELREELAQMFGIEGQLEDPRRSGWQLVFVDRENDALLLGDDPWEAFVNNVWYIKILSPQDVQELREQVIESFNPIDGQRLTSGSNEAENVSGLPSVGSLEY

>CpARF04

MNRGMDSVKEPMRNVEKCLDSQLWHACAGGMVQMPSVNSKVVYFPQGHAEHAQGNVDFGNARIPSLIPCRVSATKYMADLETDEIFAKISLLPLRNNEFNLDDDYELLGHDDIRNQEKPSSFAKTLTQSDANNGGGFSVPRYCAETIFPPLDYSAEPPVQTILAKDVHGEIWKFRHIYRGTPRRHLLTTGWSNFVNQKKLVAGDSIVFLRAETGDLCVGVRRAKRGIGCGIDYSPGWNPANSGSSLMGYSDFMRENEGRMVRRHSNGNLNGRVKVESVIEAATLAASGKRFEIVYYPCAGTPEFVVRASTVRSAMQIHWYSAMRFKMPFETEDSSRISWFMGTISSIQVADPVRWPDSPWRMLQVSWDEPDLLQNVKSVNPWLVEVVVNMPAIHVSPFLPPRKKPRYPLQAETAVFGHLPMPSFSTNFFEATNPFQSITASNIPAGIQGARHTQFGLSSSNLQISIPPLRQFPAGLKHLEGATPIPVVRGEDSFGGTKSPDNQSHWLTVGNHIQSSKETKETKPDHIILFGQLILPNQQSSNSSSGDTTNASDANLEKASNLSDGSGLSSQQNGSLDNSSDGGSTSYNGPNKTGLSLDIGHCEVFMELENVSHTLDLSVLGSYEELYRKLGNMFSVGRSEMLNSVLYQDALGATKQAGDEPFSKFMRTARRLTILANSRSRFSKKRQY

>CpARF05

MTSAEASIKPNSASAFKNHADSTKAPSDPPNALSAKDADIALYTELWNACAGPLVSVPRENERVFYFPQGHIEQVEASTNQVPDQQMPVYDLPSKILCRIINVCLKAEADTDEVFAQITLLPEANQDEDSVDKEPPPPPPRRFHVHSFCKTLTASDTSTHGGFSVLRRHADECLPPLDMSQQPPTQELVAKDLLGNEWRFRHIFRDANGSKFMFRTSPSEFVVPYDQYMESIKKSYTIGTRFKMRFEGEEAPEQRFTGTVIGCEDADPKRWKDSKWRCLKVRWDETSTISRPEKVSPWKIEPALAPPALNPLPMTRPKRHRSNMVPTSPDSSVLTREGPSRMIIDHSPASAFTRVLQGQEFSTLRGNLIDGNESDTAEKSVMWSPSLDDEKIDVVSTSKKHGADSWMPAGSGEPTYADLLSGFGANIDSSCGVRATLGDPAIVTANSIRKHAVYQDGKFNFLGGGSSWSVLPSGLSLNLVDSSQKAHIQAGDLPYQMRGNATFNGVGDHSMAQCYRIDHSMAHCSRIEQPNGNWSMPPPSSHFDYPVHSTELTSKPMLFQNQDILNLKDGNCKLFGISLVKNPAISDPAELHRNVMNEADVIHANRHPIHLHESELSRGSNLADKSLAINVADKLQHACTPNLKDSQSKSQGSSTRSCTKVHKQGIALGRSVDLSKFNNYDELIAELDQLFEFGGELLPPKKSWLVVYTDDEGDMMLVGDDPWQEFCGMVRKIFIYRREEVQKMNPRSLTLKGDENPSVEGEEAKEIKSPAVPSMSAPES

>CpARF06

MGSVDEKLKTIGGFITHAPQTNLLDDMKLLKEMQDQTGARKAINSELWHACAGPLVSLPHMGSLVYYFPQGHSEQVAVSTKRTATSQIPNYPNLPSQLMCQVHNVTLHADKDSDEIYAQMSLQPVNSEKDVFIVPDFGLRQSKHPNEFFCKTLTASDTSTHGGFSVPRRAAEKLFPPLDYTMQPPTQELVVRDLHDNTWTFRHIYRGQPKRHLLTTGWSLFVGAKRLRAGDSVLFIRDEKSQLLVGVRRANRQQSMLPSSVLSADSMHIGVLAAAAHAAANRSPFTIFYNPRACPSEFVIPLAKYRKCVYGTQLSAGMRFGMMFETEESGKRRYMGTIVGISDLDPLRWPGSKWRNLQVEWDEPGCCDKQNRVSSWDIETPESLFIFPSLTSGLKRPLHGGFLGETDWGSLVKRPMLRVPENVSYASTLCSEPLMKMLLRPQLVNLHGTTLQQQDMMGVMQPVTNPKIQQQVTPSSQHQNHHQPAPSPSTDPINSNSSPKANEPGKVQDPAAIESEAPTGAEADKSKYEREVSTCQSNPLPPVDCGEDKLTGNEVNMQTLVNQLSFVNQNQIPMQLQSVSSWPMQPQLESLIQHPQPIDMPQPEFPNSNGLISSMDGDGCLINPTCLPLSGVMRSPGNLSMLGLQDSSTVFSEALNFPIPSTGQEMWDPLSNIRFSSQNNHLVSFSHPDASNLNCMANANVTRDVSDESNNQSGIYGCSNLEISNGGNTLVDPAVSSTILDDYCTLKDADFPHPSDCLAGNFSSSQDVQSQITSASLGDSQAFSRQEFHDNSGGTSSCNVDFDEGNLLQNGSWKQVVPPMRTYTKVQKAGSVGRSIDVTSFKNYEELCSAIECMFGLEGLLNDPRGSGWKLVYVDYENDVLLIGDDPWEEFVSCVRCIRILSPSEVQQMSEEGMKLLNSAMMQGMNCSISEGGRA

>CpARF07

MTFHGGDSYTQSCVSAQGRGRDDLYMELWRACAGPLVDIPRVDEKVFYFPQGHMEQLEASTNLELNKRIPLFNLSSKILCRVIYIEPRADHESDEVYAQITLLPEANQSEPRSLDPCPPEPVKPVVHSFCKVLTASDTSTHGGFSVLRKHANECLPPLDMTQATPTQDLVAKDLHGYEWRFKHIFRGQPRRHLLTTGWSTFVTSKRLTAGDSFVFLRGDNGQLRVGVRRRAQQQSSMPPSVISSQSMHLGVLATASHAVTTQTRFVVYYKPRTCQFIISLNKYLEAVNYKFSLGMRFNMRFEGEDSPERRFSGTIIGAGDISPHWANSSWRSLRVSYSYSIPHREHHYGADLTKSAHWDSGLAQSHDGKQCGNATESRKGENNELCHHSEIDTISNSTCVSRTPTDGTWLFSTQSNGYKDPVNDTAQDYKTVPVCGWSILSGPSKVSDDQILDPTENGWKGETVASCRLFGIDLNHFAKLPAEKASSQPSSMSSDTDGRISTLSLAQSDSKSDNIEASIERKLEPLQASLKETQSSSTNTRSRTKVHMHGMAVGRAVDLTILEGYDQLIDELEKMFDVRGQLSARGKWEIVYTDDEGDMMLVGDDPWEEFCNMVRRIFICSREQVKKMSSGSKQLASIEVEGAVVIPDSPAI

>CpARF08

MFIALHLGENAIIRMKEADKNLDPQLWHACAGGMVQMPAINSKVFYFPQGHAEHAQAAVDFTSSLRIPPLIPCRVLAVKFLADHETDEVYANVRLVPLANNEINCEEEGGFGSNGSENNMEKPASFAKTLTQSDANNGGGFSVPRYCAETIFPRLDYTADPPVQTVIAKDVHGEVWKFRHIYRGTPRRHLLTTGWSSFVNQKKLVAGDSIVFLRSKNGDLCVGIRRAKRAIGCAADHPYGWNPGGGNSIPPYGGLTMFLRDEDNKLSRKGSLSSNGGGSLRGKGKVRPESVIEAAALAASGQAFEVVYYPRASTPEFCVKASSVRAAMRIQWCSGMRFKMPFETEDSSRISWFMGTISSVQVADPIRWPNSPWRLLQVTWDEPDLLQNVKRVSPWLVELVSNMPVIQLSPFSPPRKKFRLPQHPDFPLDSQFQLSSFPSNTLRPSSPMCCLSDNTSVGIQGARHTQFGISSSDFHLNNKLQLGLLPSSFQQLDFHSRISHRSVTDHRGSGSQNSSALLNAENTGPKLERSDSVKKYQFLLFGQPILTEQQITRSSSGDVHSPRTEKSSLDVNLERVKFLSDGSGYTFKQQQISPNKSPGAGFPWYQGYQATELGLDIGHCKVFMESEDVGRTLNLSVIGSYEELYRRLANMFGMEKPDILSHVLYQDATGAVKQAGDKPFSDFIKTARRLTILTDSGSDKMVGRRLMDGLRSGENGLDSSNKTGPLSIFA

>CpARF09

MEIDLNLTASDVGKNAYCNGNCEEGRCNYCLSSSTSSCSSNSSPALVSSSTYLELWHACAGPLTSLPKKGNVVVYFPQGHLEQIASASPFSPMEMRTFDLQPQILCRVINVHLLANKENDEVYTQLTLLPLPEFLGTGLEGKELEELALNGADGDGSGVSPTRSTPHMFCKTLTASDTSTHGGFSVPRRAAEDCFPPLDYTQLRPSQELIAKDLHGVEWRFKHIYRGQPRRHLLTTGWSLFVSQKNLISGDAVLFLRGENGELRLGIRRAVRPRNGLPDSIVGNQNSCADDLARVVKAISTKSIFDVFYNPRAYHAQFVISCQKYVKSINNPVTVGTRFKMRFEMDDSPERRFNGVVLGIGDMDPLRWPNSKWRCLTVRWDKDGDHQERVSPWEIEPSVSLPPLSVQSSPRLKKLRTSLQAAPPNAFAVGRGGFMDFEDSIRSSKVLQGQENVGIVSPFYGRDTTKRSLEFEVRSSAHQASGGAEKYRGDYVKVHPNSSFTGFMESERFLKVLQGQEICSLRPQTRKPEHCLGVWGKFNLSDNSFNTFHSPNSSFYHMASNGAQNMYFPRSEFYSTGQAAAVMRSNDGNFPRESALFSPSVDASVMSTTSGSNIKNSKDENVNENSTGCRLFGFSLTTETATNMQSSGKRSCTKVHKQGSLVGRAIDLSRLSGYTDLLSELERLFCMEGLLKDPDKGWRVLYTDNENDVMVVGDYPWLDFCDAVSKIHIYTQEEVEKMTNGVISDDTQSCLDQAPLCMEASKSSSVGQPDSSPTVGAAHEYRL

>CpARF10

MSSVEDKLKTPMGLVNNTPQTNLLDEMKLLKQMQDQTGARKAINSELWHACAGPLVSLPHVGSLVYYFPQGHSEQVAVSTKRTATSQIPNYPNLPSQLMCQVQSVTLHADKDSDEIYAQMSLQPVNSEKDVFLVPDFGLRPSKHPNEFFCKTLTASDTSTHGGFSVPRRAAEKLFPPLDYTMQPPTQELVVRDLHDNTWTFRHIYRGEDEKSQLLIGVRRANRQQTTLPSSVLSADSMHIGVLAAAAHAAANRSPFTIFYNPRACPSEFVIPLAKYRKCVFGTQLSAGMRFGMMFETEESGKRRYMGTIVGISDLDPLRWPGSKWRNLQVEWDEPGCCDKQNRVSSWEIETPESLFIFPSLTSGLKRPLHGGFLGETDWESLVKRPMLRVPENIRGDLSYAAPNLCSEPLMKMLLRPQLVSLHGATLHQDSANNLVKIQDMKDMQPETNSKMKQPIPTETASPGNQNPDPINPNSSPKESLPGKVHTSAATESEPPTGADDVSTNQSNTLPPVGDCVGGKLSAKEVNMQTLVNQLSFMNQNQIPMQSWPLQPQLESLIQHPQPIDMPQPEFTLDGDGCLINPSCLPLPGVMRSPGNLSMLALQESSTVFPEALNFPLPSTGQDMWDPLNNIRFSSNLNCMGNANVMRAVSDESNNQSGIYSCSNLEISNGGNTLVDPAVSSTILDDYCALKDADFPHPSDCLVGNFSSSQDVQSQITSAASLGDSQAFSRQDFHDNSGGTSSCNVDFDEGSLLHNGSWNQVVPPMRTYTKVQKAGSVGRSIDVTSFKNYDELCSAIECMFGLEGLLNDPRGSGWKLVYVDYENDVLLIGDDPWEEFVSCVRCIRILSPSEVKQMSEEGMKLLNSAMMQGINCSMPEGGRG

>CpARF11

METNFLFFLLLFDHRRSDSGSMDRQLWQAFAGKSVHIHTVGSEVYYFVQGHVEQATYIPSLSQSVLSNPATKCLVTGTDFHADSLSDEVCIKLNLHPIRPGRDESVVVSRFGRCDDNGGERDKIESFAKILTSSDANNGGGFSVPRCCADSVFPPLNYQADPPVQTLSITDVHGVVWNFRHIYRGTPRRHLLTTGWSKFVNHKKLIAGDSVVFAKNLRGDMFVGIRRASTSITRAISGGDCGRWNSQNGGARCSLEENCSGDGGTKVFSRRNIGKLPAEAVANAAELAAQFKPFELVYYPRAGRSEFVVQVEKVNKSKNYQWYSGTRVKMAMETEYSKMTWYQGTVTSASVPEHGPWMGSPWRMLEVTWDEIDALQSAKYVSPWEVELATPTPPIQPPLHPAKKFRGLPKSGMLNGEAELFSPLMNFVDSTMEQFNPSLLNLNSFPAGMQGARQKLFCESRLFNPCKEMTPLTCDESSMSELNAAKTMQMVSTDLLIGSVQFDTLSSDSQASVLSFATGTAENQNCCNSAKAGVNSIQLFGQVIYMNPPVENGLENGVSTNDGGKNLNQYLGQGRQEADYNMNIGSRYFSPASHSSPVNKEEDDGEDGNKAGDELHGLSLQALINVYREAFLDGDQKTISKVEAKLKNFEREKDGLFQKVSNTSAEITSGKENYIRLQADFDNFRKRSEKERLTVKNNAQKEVIENLLPMIDSFEKARQQIVPQTDKEKKIDVSYQGIYKQFVEVLRSWRISAVAAVGRPFDPSLHEAVAREESQEIKEGIIIQELRRGFLLGERLLRPARVKVSKGPGKKNSPTTNSDIPTEQPATATARLDEH

>CpARF12

MKTPANGVASSAAAPNSCEGGLEKKIINPELWQACAGPLVNLPPAGYHVVYFPQGHSEQVAASLKKDVDAQIPNYPNLPSKLLCLLHNVTLHADPETDEVYAQMTLQPVPSFDKDALLRSDLALKSNKPQPEFFCKTLTASDTSTHGGFSVPRRAADKIFPPLDFSMQPPAQELVAKDLHDTVWTFRHIYRAAAHAAANNSPFTVFYNPRASPSEFVIPLAKYYKAVSANQISLGMRFRMIWKGSQWRNLQVGWDESTGGERRNRVSIWEIEPVIAPFFICPPPFFRSKRPRQPGMPDDESSDLDSIFKRTMFGDDFCMKDPQGYPGLNLVQWMNMQNPSLSNMQPNYMHSFSGSMLPNLGGVDISRQLGMSNAQNPQSNNIQFNAQRLLPQAQQLDQLPKIPSTMNSMASVVQPPQQFDDISQQTRQNVVTSQIQSQIMQQPHANGILQQQTSLQNQQLQRSLPQNMLMQQHQQILGQNQQQNMNQPPIPDQSNHPLQMSDNQIQMQMLQKFQQQQQSLLAQQSVLQPAQLAQLPEQQSFSISMSTNQTMDVPHSAPAAGPPPSAQQAPKSHSSNQHLQPKFPQLQQQPHDMSRPMGHNNQFSAATSSVITGAAGAGQSGITDDIPSCSTSPSTNNSPSLVQPVTNGRLHRTTGLVDDVAQSAATIFSTNTLDSISPNANLVKNIPHKAAVKPSLNISKNQSHGVLTQQTFLTGAVPQTEFLDTSSSTTSACLSQNDAQLQQNNIMSFNSQPMLFRDGSQDLEPPTDLHNIPYGTNVDGQLVAHLNSDPLMNKGIGGLGKDFSNNFSSGDMLATYDAQKDPQQEISSSIVSQSFGIPDMTFNSMDSTINDSSFLNRNQWAPPPPFQRMRTYTKVYKRGAVGRSIDITRYSGYDELKQDLARRFGIEGQLEDRQKIGWKLVYVDHENDVLLLKVHSFIYHYGYDSLLAMEEIL

>CpARF13

MKAPPNGFVPNSGEGTGERKNINSELWHACAGPLVSLPPVGSLVVYFPQGHSEQVAASMNKETDFIPNYPNLPSKLVCMLHNVILHADPETDEVYAQMTLQPVNKYEKEALLASDIGLKQNRQPAEFFCKTLTASDTSTHGGFSVPRRAAEKIFPPLDYSMQPPAQELVARDLHDNSWTFRHIYRGMGPFVFSPLLSLDEKSQLLLGIRHANRQQPALSSSVISSDSMHIGILASAAHAAANNSPFTIFYNPRASPSEFVIPLAKYTKAMYTQVSLGMRFRMMFETEESGVRRYMGTITGISDMDSVRWKNSQWRNLQVGWDESAAGERPNRVSIWEVEPVVTPFYICPPPFFRPKFPKQPGMPDDESDIENAFKRAMPWFGDDFGMKDTPSSIFPGLSLVQWMSMQHNNQFPAAQSGILPSMLQQQQLLAGNVQSHQTSQPSNKNSIETTSLLQETQFQPQIDQQPSLVSKHHQQTQFQQAPLQLLQQSLSQRTQQLPQVQQFSQPIIPSEQQLQWQLLQKLQQQQQQQQQQQQQQQPLVSPASPLFPPQMIQPHPVHQQNQQLPPLPLSNQPQFNASGGSFQTEKHNSNGFSSLGLMQSQQVPITQSHNQFKPTTAIRGYSGLTDGDAPSCSTSPSTNNCQVSVSNLLNKNQQGAATLGGDLVVEPATNLPQELPSKPDLRNKPEFPNSKGLDQLKYKGTVPDQSFGVPNLPFKPGCSNDVNVNEAGALSSGLWANHGQRMRTYTKIETKAAKIMKVQKRGSVGRCIDVTRYKGYDELRHDLARMFGIEGQLEDPQRTDWKLVYVDHENDILLVGDDPWEEFVSCVQSIKILSSSEVQQMSLNGNLGHIQAPNQACSGTDSGNAWRGQYDDNSAASFN

>CpARF14

MSFSAVSSLPPGGPTSGPSSDALYRELWHACAGPLVTLPRQDERVYYFPQGHMEQLEASMHQGLEQQMPSFNLPSKILCKVVNVVLRAEPDTDEVYAQITLLPESNQSEVTSPDPALPEPTRCNVHSFCKTLTASDTSTHGGFSVLRRHADDCLPPLDMSQQPPWQELVASDLHGNQTSRSTFLVSLNKYLEAQNHKLSVGMRFKMRFEGEEVPERSFSGTIVGLGDNASPGWANSDWRSLKVQWDEPSSILRPDRVSSWELEPLVASSPLSSLPAQRNKRPRPAVLPSPSADAAVLGGWKPTVESSAFSYTEPQRGRDLYSSPKFSTAASNSLGFNGNNSLGAVSSNAYWSNTSMVENVMDTSSHGAKREPVEKKQNTRNGCRLFGFQLLGNSNVEEASPVSTPKMVGEDRPVPPIDTAFDQHFDPSNIHRSDMPSVSCDADKSCLISPLESQSRQIRSCTKVQMQGIAVGRAVDLTRFNQYDDLLRKLEEMFDIEGELCGSVKKWKVVYTDDEDDMMMIFIYTTEEVKRLSPKIKLPLSGEMKLSKPDSDMVANHTEDQSFSSVRLFFGAFLSPDYKTVQVRSLFLYGLSMTIREEEAQTSSTLL

>CpARF15

MRLSAAGFSPQAPEGERRVLNSELWHACAGPLVSLPAVGSRVVYFPQGHSEQVAASTNKEVDTQIPNYPSLPPQLICQLHNLTMHADADTDEVYAQMTLQPLSAQELKEAYLPAELGTPSRQPTNYFCKTLTASDTSTHGGFSVPRRAAEKVFPPLDFSMQPPAQELIARDLHDNEWKFRHIFRGMKRTNYSSESGVQVDRKPNEKNQLLLGIRRASRPQTVMPSSVLSSDSMHLGLLAAAAHAAATNSRFTIFFNPRASPSEFVIPLAKYVKAVYHTRVSVGMRFRMLFETEESSVRRYMGTITGISDLDPVRWQNSHWRSVKVGWDESTAGERQPRVSLWEIEPLTTFPMYPSPFPLRLKRQWPTGLPSFGIKDSDLGMNSPFMWLRGDNSDRGIQCLNFQGNGVSPWMHPRLDPSMVGMQSDVYQAMAAAALQEMRAIDYSKLAPASMLQFQQPQGLPCQPSTLMQPQMLHQSQSQHAFLQSVQEHQQHSQSQTQTQSHHLQPQLQPEPSFNNQQPQHQQQPRQPQPQPPPQPQPQPLVDHQPIPCTIPAISQFASCSQSQSPSLQTVPSLCQQPSFSDSNGNPVTSPTVSPFHSLAGSFVQDESSQLLNIQRANSVIPSAGWPSKRSAIDPLSTGASQYFLPQVESGMSQNTVALPPFPGRECAIGDRDEGSDPENHVLFGVNIESSSLLMQNGMSNLRGVGNDNVSTTLPFSSNYMSSSGTDFSVNPTTNCIDESGFLQPHENVGQVNQPNGTFVKVHKSGTYSRSLDITKFNNYLELRSELARMFGLEGELEDPLRSGWQLVFVDRENDVLLLGDGPWPEFVNSVWCIKILSPDEVQEMGKRGLELLNSMPIQRLSNNTCDDYGSRQDSRNLISGITSVGPFDY

>CpARF16

MRLSSAGFSPQPPEGERRVLNSELWHACAGPLVSLPAVGSRVVYFPQGHSEQVAISTNREVDAHIPSYPSLPPQLICQLHNVTMHADVETDEVYAQMTLQPLSAQEQKEPYLPAELGAPSKQPTNYFCKTLTASDTSTHGGFSVPRRAAEKVFPPLDFSQQPPAQELIARDLHENEWKFRHIFRGQPKRHLLTTGWSVFVSAKRLVAGDSVIFIWNEKNQLLLGIRRANRPQTVMPSSVLSSDSMHLGLLAAAAHAAATNSRFTIFYNPRACPSEFIIPLAKYVKAVYHTRVSVGMRFRMLFETEESSVRRYMGTITGISDLDSTRWPNSHWRSVKVGWDESTAGERQPRVSLWEIEPLTTFPLYPSPFSLRLKRPWPTGFPSFHGLKEDELGLNSQLMWLRGDGLDRGIQAMNFPGIGVAPWMQPRLDASMVGMQPEIYQAMAAAALQEMRTVDPAKVQAASLLQFQQTQTLPNRPATFMSPQILQQPQPTFIQGDDNQRLSRSQAQTLPTVLQQEIKHQTFNNQQQQQQQVFDHQQIPSTITTMSQFSSASQSQAQSLQTTPPLCRQQSFSDSNPNHVTSPIISPLHSLLGSFPQDESSQLLNLPRTNPMVHSSTWPSKRAAVDPLLSSVNSQFVLPQGENMGTTQTNISQNAISLPPFPGRECSLDQGNADPQSNLLFGVNIEPSSLLMQSGMPNLRGICSDSDSTAIPFSSNYVNTAASNFSANPTGTPSNCIEDSGFLQSPENTGQVNPSTRTFVKVYKSGSLGRSLDISKFSSYHQLRSELAHMFGLEGELEDPVRSGWQLVFVDRENDVLLLGDDPWPEFVNSVWCIKILSPQEVQEMGKQGLELLKSVPIQRLSNGSCDNYANRQESSRNLSTGITSVGSLEY

>CpARF17

MGALIDLNTTEEDESPSSAASSVSSSSASALTSSPSPSLTSSICLELWHACAGPLTSLPKKGNLVVYLPQGHLEQMQEFPATPSDLPPHILCRVIDVQLHQAEAGSDEVYAQVSLLPENEQMEHKMQEEMSNDIEEEDVEEGEKTTTPHMFCKTLTASDTSTHGGFSVPRRAAEDCFPPLDYNQQRPSQELVAKDLLGLKWKFRHIYRGQPRRHLLTTGWSAFVNKKRLVSGDAVLFLRGNDGELRLGIRRAAQLKSGSTFSNICSQKLNSSSIMDVVNAISSKSSFSVCYNPRATSSQFVLPFHKFLKSMNRSFSVGMRFRLSFETEDAADLRHTGHITGIGDVDPIGWPGSRWRSLMVRWDDGEINRHGRVSPWEIEPSGSVSMSSNLVPPGLKRSRIGLSSTKLEFPVPHGIGATDFGESLRFHKVLQGQEILGYNTPTDCDDNTRYHPPEKRRLLPDLHGSGIALMRNGPRNRLINSETSSRGFVFDESFQFQKVLQGQEIFPGPFYGRAMATNEVKESGGCGPVDGFRLSTTRDGWPTTMQCENFHTRSSIPSAQVSSPSSVFMFQQSMVPVLSFDSHNRGIMNKSTSHNSGTAFMTDNSMCPTSLGELNLLGLSHPSTTTASAFSGSKDLSSTTKAGCRLFGFSLTEEKNVGNTDKGSPATTPIHAGTTIGAQCPLKSPLMSKVVGSNCTKGALQYHYANYSTYY

>CpARF18

MDPKKKMITFMDSKEKSKEMDKCLDPQLWHACAGGMVQMPPVNARVFYFPQGHAEHACASPVDFRTCPKLPSYTLCRVSAIKFLADPDTDEVFAKLRLIPINGSELDFDDGIGRLNGSEQDKPTSFAKTLTQSDANNGGGFSVPRYCAETIFPPLDYSADPPVQTILAKDVHGETWKFRHIYRGTPRRHLLTTGWSSFVNHKKLVAGDSIVFLRAENGDLCVGIRRAKRGAGDGPDSSCGWNGAVPYGAFSANESLMGKGKVKAKSVIEAATLAANGQPFEIVFYPRASTPEFCVKAALVKAALQIRWCSGMRFKMAFETEDSSRISWFMGTVNSVQVADPLRWPESPWRLLLNVKRVSPWLVESVSNMSPIHIAPFSSPRKKLRYPQHPDFPLDNQPPMPLFSSYLHGSGSPFGCPPDNNSAGMQGARHAHFGLSLSDFHLSKLHSGLFPIGYRSPDPAAESATLSCNAMTEKPSMSENVSCLLTMAHSTQASKKCDGDDRQEVEPNVDPGHCKVFMESEDVGRTLDLSSLGSYEELYTKLGNMFDIDNSETLNHVLYRDVSGAVKHVGDEQFSDFIKTARRLTIL

>CpARF19

MDPKLWRAFAGDLAHLHTVGSEVYYFVQGHVEQATYAPKLSPAVLSNPVSKCLVTGVGLDADALTDEVLIKINLHPIRPGEGRSEVVSRLGCSEVNVISKFAKVLTSSDANNGGGFSVPRSNRPFTSGECSRVEENPSGDGDPRNFSRRTIGRVPPEVVATAAELAAQFKPFEVVFYPRIGLSQFVVPVEIVNNSMKYQWYPGIRVKLPTETEDTLRTQWHQGTIISVSIPEHDPWKGSPWRMLEITWEETDAPPNGKYVCPWEVELAGPAPPIQPSLHIAKRPRGHSKSGQLNGEAELFSPMMRVGDSSMEQFNQALLSFNSFPAGMQGARQNFLCESGLFDNPYKETTNAEPSTQMVSQVVSTDLHIGSAQSDTLSPDSQASVLSFATESADNQLSNSTEAGVTSFQLFGQIIHLNPPPENGANTDDVDMTSNQSD

>CpARF20

MKTPANGAAAMPNSSEGGLEKKTINPELWQACAGPLANLPPAGYHVVYFPQGHSEQVAASLKKDVDGQVPNYPNLPSKLLCLLHNVTLHADPETDEVYAQMTLLPVPSFDKDALLRSDLSLKSNKPQPEFFCKTLTASDTSTHGGFSVPRRAAEKIFPPLDFSMQPPAQELVAKDLHDNVWTFRHIYRGQPKRHLLTTGWSLFVSGKRLLAGDSVLFIRDEKQQLLLGIRRANRQPTNLSSSVLSTDSMHIGILAAAAHAAANNSPFTVFYNPRASPSEFVVPLAKYYKAVSANQISLGMRFRMMFETEESGTRRYMGTITGISDLDSVRWKGSQWRNLQVGWDESTGGERRNRVSIWEIEPVIAPFFICPPPFLRSKRPRQPGMPDDDSSDLDGICKRTMFGDDFCMKDPQGYPGLNLVQWMNMQNPSLSNAMQPSYMHSFSGSMLPNLGGVDISRQLGLSNTQIPQSNNIQFNAQRLLSQAQQLDQLPKLPSSMNSMGSVVQPSQQLDDMSQQTRSNLINQNAVSSQIQAQIMQQQQPHTNGIVLQQNQQLQRSLPQNQSQTMNQSPLPDPMIHQLQMSDNQVPMQMLQKLQQQQQQQQSLLAQQSVVLQPAQLPEQLRQSVDASFSRSMSSNQMLDIPQSTPAAVPPSNVSPQQAAKSNGTTNNRFSNQHMQPKLPQLHQPPPSTVLSDMSRPVGLPPTQTYNQLSAATSSVITGAAAAGQSGVTDDIPSCSTSPSTNNGPSLIQPVNNGRVHRSTGLVEDVAQSSATIFSTNTLTAVKPSLNISKNQSHGIFAQQTFLSGVVSQTDFLDTSSSTTSACLSQNEAQLQQNNVMSFNSQPMLFRDSTQDLEVPTDLHNIPYGTNVDGPLVAQLNSDPLLNKGIGELGKDFSNNFSSGAMLTTYDAQKDPQQEISSSIVSQSFGIPDMTFNSMDSAMNDNAFLNRNQWAPPPPFQRMRTYTKVYKRGAVGRSIDITSYSGYDELKQDLARRFGIEGQLEDRQKIGWKLVYVDHENDVLLVGDDPWEDFVNCVRSIKILSPQEVQQMSLDGDFGNGVLLNQACSSSDGGNA

>CpARF21

MGMKAPPNGFLPNSGEGERKNINSELWHACAGPLVSLPPVGSLVVYFPQGHSEQVAASMNKETDFIPSYPNLPSKLICMLHNVTLHADLETDEVYAQMTLQPVNKYEKEALLASDIGLKQNRQPAEFFCKTLTASDTSTHGGFSVPRRAAEKIFPPLDYSMQPPAQELVARDLHDNSWTFRHIYRDEKSQLLLGIRRANRQQPALSSSVISSDSMHIGILASAAHAAANNSPFTIFYNPRASPSEFVIPLAKYNKAMYAQVSLGMRFRMMFETEESGVRRYMGTITGISDMDSVRWKNSQWRNLQVGWDESAAGERPNRVSIWEVEPVVTPFYICPPPFFRPKFPKQPGMPDDESEIENAFKRAMPWFGDEFGMKDASSSIFPGLSLVQWMSMQHNNQFPAAQSGILPSMVAPSALHGTLTNDESSKLLSFQAPLIYHHCLCPISSSSILVEGAYKQKNITAMAFQLPPLPLSNQQQFNTSGGSLQTEKHNSNGFSSLGLMQSQQAPITHSHNQFKPTMAIRAYSGLTDGDAPSCSTSPSTNNCQVSASNMLNKNQQGAATLGGDPVAEPATNLAQDLQRKPDLRIKHEFPSSKGLDQLKYKGTVPDQLEACSSGTSYCLDAGTIQQTFPLPTCLDNDVQSHPRNNIPFSNSMDGLAPDTLLSRGYDSQKDLQNLLSNYGGGVPRDIETELSTAAISSQSFGVPNLPFKSDCSNDVNVNEAGALSSGLWANHSQRMRTYTKGNHRTFVVSRNEEEMRDLVIDPQRTDWKLVYVDHETDILLVGDDPWEEFVSCVQSIKILSSAEVQQMSLDGNLGHIQAPNQACSGTDSGNAWRGQYDDNSAASFNR

>CpARF22

MACNGGDSYTQSCVSTQGSGRNDLYTELWRACAGPLVDVPRVDERVFYFPQGHMEQLEASTNLELNKRIPLCNLSSKILCRVIHIELLADHESDEVYAQITLMPESNQNEPNSLDPCRPEPARPVVHSFCKVLTASDTSTHGQPRRHLLTTGWSTFVTSKRLSAGDSFVFLRGDNGEMHVGVRRRARQQSSMPSSVISSQSMHLGVLATASHAVTTQTRFVVYYKPRTCQFIISLNKYLEAVNNKFSVGMRFNMSFEGEDSPERRFSGTIIGATDISPHWPNSSWRSLRVQWDEQTSILRPDRVSPWDIEPLTSSAVTGLSQPISKNKRPRQSTPAHDGADLPKPALWDSGLAESHDGKQSSNAAESRKGENNELCHHRETDAISNRTCVSRTQADGTWLSSTQSNSHKHPVNDMAQDYKTVPGCGWSFLSGPSTSHLVKLSDEQILDSTENGRKGETVASCRLFGIDLNHLAKLPAEKASYQPSSVSSDTDGRSSTLSVAQSDPKSDNLEVSVERKSEPLQASLKETQSNQSSSANTRSRTKVHMHGMAVGRAVDLTILEGYDQLIDELEKMFDVRGQLCARDKWEIVYTDDEGDMMLVGDDPWEEFCNMVRRIFICSREQVKKMSSGSKQLTSIEVEGAAIIPDSPAV

>CpARF23

YWGKLDMKLSTSGFGQQDHEDDLYWGKLDMKLSTSGFGQQDHEGGEKKCLNSELWHACAGPLVSLPTAGTRVVYFPQGHSEQVAATTNKEVDGHIPNYPNLPPQLICQLHNVTMHADVETDEVYAQMTLQPLTAQEQKDTFLPMELGIPSRQPTNYFCKTLTASDTSTHGGFSVPRRAAEKVFPPLDFSQQPPAQELIARDLHDVEWKNEKNQLLLGIRRATRPQTVMPSSVLSSDSMHIGLLAAAAHAAATNSCFTVFYNPRASPSEFVIPLTKYVKAVFHTRVSVGMRFRMLFETEESSVRRYMGTITGISDLDPVRWPNSHWRSVKVGWDESAAGERQPRVSLWEIEPLTTFPMYPSLFPLRLKRPWHPGVSSVHDNRDDASNGLMWLRGGVGDQGLHSLNLQSMGSLPWLQQRLDSPMFGNDHNQQYQAMLAAGMPNLGGVDMLRQQIMHLQQPFQYIQQAGFHNSLLQQQQQLVQQSMPQNILQTPSQVMAENIPQHILQQTPQNQSEDIPNQQQRTYHDTLQVQSNQFHQGENSNVPSPPFPRTELMDSNTNYSESMISRRNILASSCAEGTGNLSNIYRSGQSILTEQLPQQSPVSKNAHPQVDAHSNSMTFPPPFSGRDSILELGNCNSDSPSRTLFGVNIDSSGLLLPSNVPTYASPSIGPDSSSMPMGDSGFQNSMYSCVQDSSELLHSSGQVDPLNPTRTFVKVYKSGSVGRSLDISRFSSYQELREELAQMFGIEGQLVDPHRSGWQLVFVDRENDVLLLGDDPWEAFVNNVWYIKILSPQDFQKLGVQAIESFNPVGGQRLTSGGNEAENVSGLPSVGRNSSI

>CpARF24

MTRGMDSVVEPMRNHEKYLDSQLWHACAGGLIELPTIDSKVVYFPQGHAEHAQENVDFGVAQIPSLIPCRVSGIRHMADSETDEVFANIRLIPLRNNEFSLDDDDELLGHNDIRTREKPASFAKTLTQSDANNGGGFSVPRYCAETMFPRLDYSAEPPVQTILAKDVHGEIWKFRHIYRGTPRRHLLTTGWSNFVNQKKLVAGDSIVFLRAETGDLFIGVRRAKRGNGYGIDYETGWDPTNSASPLVGYSDFMRENEGRLVRRISNGNSSGRVTVESVIEAAMLAASGKPFEIVYYPCTGTPEFVVKASCLRSAMQVHWYPAMRFKMPFETKDSSRISWFMGTISSIQVADPVRWPDSPWRMLQVAWDEPDLLQNVKSINPWLVEVVVNMPAIHVSPFSPPRKKQRFHPAETAVFGHLPMPSFSTNFFETTSSLQSVTGNNIPAGIQGARHTQFGLSSPDFQLSKLNLGPCSAGFKHLDEATPLPGSPGENMFGGMKSPDNSHWLTMGNNTESSKDSKETKPDHIILFGQLILPNQLISNSCSADTTNASYENQGKSSNPSDGSGLSSQQNGSLENSSEGGSTSYNGHEKTGFSLNTGQCKVFMEFEDIGRTLNLSLLRSYDELYGKLANMFGLGSSEMLNCVLYEDALGTTKQAGDEPFSTFLKTARKLTILTDSRSSDNSKT

>CpARF25

MANRGGGSFSSQGGGGDGLYTELWKASAGPLVEVPRVNDKVFYFPQGHMEQLEASTNHELNQKHPLFKLSSKILCRVVDVRLMAEQETDEVYAQITLMPDSNQEEPTNPDPSPPECRKPKVHSFCKVLTASDTSTHGGFSVLRKHATECLPPLDMTQQTPTQELVAKDLHSYEWRFKHIFRGQPRRHLLTTGWSTFVTSKRLVAGDSFVFLRGENGELRVGVRRLARQQTSMPSSVISSDSMHLGVLATASHAVSTLTRFVVYYKPRASQFIVSLNKYIEAMNRKFLVGMRFKMRFEGEESPERRFSGTIVGVDDISPHWPNSKWRSLRIQWDELASIPRPDRVSPWEIEPFVAPTSPSIPQSVSVKNKRLRPPLDIPDSDNSTVTTLRHPGSTQSHDDRTQLSVTAAEMKRFENHATWNYKQTDVSSIGNSISRTPKEGSWLASPNGSVSQHRLQNLTDDRNSNYVWSTVFSGAPAAQSTCPAPHPSNPKSSDEVNDLGEKGRKTEVAPSCRLFGIDIIGHSKSPVPPEMAADQPISAPNEITDAEQNSDQPKASKERKLGLLQVPPKEIQHKQSSSTNSRSRTKVRLYEIYYTDYSCLELYGHTLIYRLLVGCQYMKVQMQGMAVGRAVDLTMLEGYGQLIDELEKMFDIKGELHPRDKWEIVFTDDEGDTMLMGDYPWQEFCNMVRRIYIWSSQDVKMMSSVSKLTMSAMECDGTVITSESADS

>CpARF26

MGIQMKEAEKSLDPQLWHACAGGMVQMPAINSKVFYFPQGHAEHAQATVDFTSSLRIPPLILCRVLAVKFLADPETDEVFANVSLVPLANNDLNFEEEGGFGSNGSDNNMEKPASFAKTLTQSDANNGGGFSVPRYCAETIFPRLDYTADPPVQTVIAKDVHGEVWKFRHIYRGTPRRHLLTTGWSSFVNQKKLVAGDSIVFLRSKNGDLCVGIRRAKRAIGCAADHPYGWNPGGGNCFPPCGGLTMFLRDEDNNKLSRKGSVSSSGGGGNLRGKSKIRPESVIEAAALAASGQPFEVVYYPRASTPEFCVKASSVRAAMRIQWCSGMRFKMPFETEDSSRISWFMGTTSSVQVADPIRWPNSPWRLLQVTWDEPDLLQNVKRVSPWLVELVSNMPVIQLSPFSSPRKKFRLPQHPDVPLDSQFPLSSSSSSFSSNTLRPSSPMCCLSDNTSVGIQGARHTHFGISLSDFHLNNKLQLGLVPSSFQQLHFHSRISNTDHTRSSSALLNGEKTGPKLERSDSAKKKHQFVLFGQPILTEQQICCSSSSDIHQVSPNKSDTELGLDIGHCKVFMESEDVGRTLNLSVIGSYEELYRRLANMFGMEKPDILSHVLYQDATGAVKQAGDKPFSDFIKTARRLTILTDESGSDKMGRRTLMDGVLSGENGRLDASNKTGPLSILA

>CpARF27

MEIDLNQTASEEGKNAYCHGNCEEGRCNCCLSSSTSSCSSNSSSTPASSSTYLELWHACAGPLTSLPKKGNAVVYFPQGHLEQIASASPFSPMEMATFDLQPQILCRVINVHLLANKENDEVYTQLTLLPLPELLGTGVEGKELEELALNRAADGDGSGGSPTRSTPHMFCKTLTASDTSTHGGFSVPRRAAEDCFPPLDYTQLRPSQELIAKDLHGVEWRFKHIYRGILGENGELRLGIRRAVRPRNGLPDSIVGNQNSCANDLTRVVKAVSTKSTFDVFYNPRAYHAQFVVSCQKYVKSINNPVNVGTRFKMRFEMDDSPERRFNGVVVGIGDMDPFRWPNSKWRCLTVRWDKDSDHQERVSPWEIDPSVSLPPLSVQSSPRLKKLRTSLQAAPPNNAFNGRGGFMDFEDSVRSSKVLQGQENVGMVSPFYGCDNTAKRSLEFEVRSSAQQNQASGGVEKLNIGDYVTSFTGFMESDRFLKVLQGQEICSLRPQTRKPEPSLGVWGKFNLSDNSFNPFQSPNSSFYHMMSNGARNMHYPRRDIYSTGQAAMMSSNDINFPRESALFNPSAKMERANSTPPTLGSTMRNSKDENVHKQGSLVGRAIDLSRLNGYTDLLSELERLFSMEGLLKDPDKGWRVLYTDNENDVMVVGDYPWHDFCDAVSKIHIYTEEEVEKMTNGVISDDTQSCLDQAALCMEASKSSSVGQPDSSPTVVRV

>CpARF28

MTLSEQNPAEFRKGLEGEGLFEELWKACAGPLVEVPFNGERVFYFPQGHMEQLEESTNHELNHQIPHFDLPSKILCRVVNIRLLAEKETDEVYAQITLQPEADQSEPQCPDPEPPERTRPTVHSFCKILTASDTSTHGGFSVLKKHATECLPPLDMSQSTPTQELAAKDLHGHEWKFKHIFRGQPRRHLLTTGWSTFVVSKRLVAGDAFVFLRGDNGELRVGVRRQARQQSLMPSSVISSHSMHLGVLATASHAVRTQTFFVVYYKPRTSQFIIGLNKYLETIKNRYEVGMRFKMRFEGEESPERRFTGTIVGVGDISPIWSDSKWRSLKIQWDEAAAIQRPERVSPWEIEPFVPSASLNFTHPSIKSKRARPVEVPPPENTSSSTPTGFWLHGSTIPHEIPQLSGANDVPSSGNQVVWSLWQQTLDVNVDSSRSHCNPMAHVEGIWPSPPPPLCNNSLKPLPSPYSPSSTSKPSSELIEHDQSEKGNKPDISLGCRIFGIDLKKNSSIVPSLEKKSCCQTTVATDIAKDPVPIAAVTSQADAGKEQQQVASELSMKGTQTNHIPNSSSRTRTKVQMQGVAVGRAVDLTTLKGYEDLIDELENVFEIKGELREMNKWSIVFTDDEYDMMLVGDDPWPEFCQMVKRIFIYSSEEVKTMSSNSKLVSPPSLDSLDSERKTES

>CpARF29

MASSEVSINPNSASVPFNDHADSTKLTSDPPNALSARDADFALFTELWNACAGPLVSVPRENDRVFYFPQGHIEQVEASTSQVADQQMPVYDLPSKILCRVINVHLKAEPDTDEVFAQITLVPEANQDEHAVDKEPPPPPPRRFHVHSFCKTLTASDTSTHGGFSVLRRHADECLPPLDMSRQPPTQELVAKDLHGNEWRFRHIFRGQPRRHLLQSGWSVFVSSKRLVAGDAFIFLRGENGELRVGVRRAMRQHGNVPSSVISSHSMHLGVLATAWHAISTGTMFTVYYKPRTSPSEFIVPYDQYMESIKKSYTIGMRFKMRFEGEEAPEQRFTGTIIGCEDADPKRWKDSKWRCLKVRWDETSTISRPEKVSPWKIEPALAPPALNPLPMTRPKRPRSNMVPTSPDSSVLTREGSSRVTVDPSPVSAFTRVLQGQEFSTLRGNFIDGNDPDAAEKSVMWPPSLDDEKIDVVSTSKKHGADCWITPGRSEPTYADLLSGFGTNIDSSHGVRAGMGDPAVVTANSIRKHVMDQDGKFNFLGGSSWSVLPSSLSLNLVDSSQKGHIQAGDLSYQVRGNATFNGFGDHSVAHCHRTEQPHGNWLMAPLSSHFDYPVHSTELMSMPMVFQNQDIMKPKDGNCKLFGISLIKNPAIPDPAGLNRNMNGADVTHLNIHQIHSNESDLKSEPFRGSMLADKSLAINDADKLQQTCTQNLKDAHCKSQGTSARSCTKVQKQGIALGRSVDLSRFNNYDELVAELDQLFEFGGELLAPKKNWLIVYTDDEGDMMLVGDDPWQEFCGMVRKIFIYTREEVQKMNRGSLNLKGDENPSVEGKEAKEIEGQAVPSISAPESS

>CpARF30

MITFMDSKEKVKEMEKCLDPQLWHACAGGMVQMPPVNARVFYFPQGHAEHACAPVDFRNCSKVPPYTLCRVSAIKFLADPDTDEVFAKLRLIPINGSELEFEDDGIGRLNGSEQDKPTSFAKTLTQSDANNGGGFSVPRYCAETIFPRLDYSADPPVQTILAKDVHGETWKFRHIYRGTPRRHLLTTGWSTFVNHKKLVAGDSIVFLRAENGDLCVGIRRAKRGIGDGPEPPCGWNPAGGNCAVPYGAFSTFLREDENRVNRTNGKGKVKAESVIEAATLAANGQPFEIVYYPRASTPEFCVKAGLVKAALQIRWCSGMRFKMAFETEDSLRISWFMGTINTVQAADPLRWPESPWRLLQVTWDEPDLLQNVKRVSPWLVELVSNISPIHLAPFSPPRKKFRYPQHPDFPHDNQPSMSPFASYLHGPGSPFGCPPDNNPAGMQGARHAHFGLSLSDFHLSKLQSGLFPIRYRSLDPAAGSTRLSGNAMTEKPSMSENVSCLLTMAHSTQTSKKFDNVKTPQLILFGRPILTELQMSQSCSGDTVSPVGTGNSSSDGNGSGSALHQQGLPERSSCENFQWYKDNRQDVEPNLDTGHCKVFMESEDVGRTLDLSSLGSYEELYRKLGNMFGIDNSEMLNHVLYRDISGAVKHVGDEQFSEFMKTARRLTILTDSGSNNVILVLNVNGSGFKFKEVSRDTSRPLQCDV

>CpARF31

MAALIDLNTTEEDDAPSSAASSASSSSASALTSSPSPSLTSSICLELWHACAGPLTSLPNKGSLVVYFPQGHLEQMQEFPATAAYDLPPHILCRVIDVQLHAEAGSDEVYAQVSLFPENEPIEHKMQEEMTNDSEGEDFEGSEKTTTPHMFCKTLTASDTSTHGGFSVPRRAAEDCFPPLDYSQQRPSQELVAKDLLGIKWKFRHIYRGQPRRHLLTTGWSAFVNKKRLVSGDAVLFLRGNDGELRLGIRRAAQLKSGSPFSNICSQQLNSSSIMDVFNAISSKTSFSVYYNPRATSSQFVLPFHKFLKSINHSFSAGMRFSLSFETDDAADRRCTGRITGVGDVDPIRWPGSRWRSLVVRWDDVETKRHGRVSPWEIEPSGSVSVASNVVPPGLKRTRIGLSPTELEFPVPNGIGASDFGESLRFQKVLQGQEILGYSTPIDGDDNIRHPPEKRRSFPGLLGSGIALMRNGPRIPVTNSETSSRGFMFDESFQFHKVLQGQEMYPSPFFGIATATNEVKASGGYGPVDSVPLSRSKDGWPMTMQSENFLTRSSIPSVQVSSPSSVFMFQQSMVPVPSFNSYNRGNFTEQRTMNKSTSHNSGTAFMTDHNSSTEVPQGMRPTSLGVQKQLGLSHPSTTEPAFTGNKAGCRLFGFSLTEGKNVGNTTDKASPATTPINAGTTSVLSSNSGLRCPLKSPLMNKVVGSNCTKGAVQYHFANCSTYY

>CpARF32

MKSVIEEIDFGASNLGVNTSLGLLDLTEMRLSAAGFSPQPPEGEKRVLNSELWHACAGPLVSLPAVGSRAVYFPQGHSEQVAISTNKEVDAHIPSYPSLPPQLICQLHNVTMHADIETDEVYAQMTLQPLSAQEQKEPYLPAELGAPSKQPTNYFCKTLTASDTSTHGGFSVPRRAAEKVFPPLVILKMNEKNQLLLGIRRASRPQTMMPSSVLSSDSMHLGLLAAAAHAAATNSRFTIFYNPRASPSEFIIPLAKYVKAVYHTRVSVGMRFRMLFETEESSVRRYMGTITGISDLDPARWPNSHWRSVKVGWDESTAGERQPRVSLWEIEPLTTFPMYPSPFSLRLKRPWPTGLPSFHGLKEDDLGLNSQLMWLRGDALDRGIQPLNLHGIGVAPWMQPRLDASMVGLQPEIYQAMAAAALQEMRTVDPAKAQAASLLQFQQTQNLPNRPATFMSPQMLQQPQPHQTFIQNDHEIQHLPHSQAPTQPTVLRQEMKHQTFNNEQQQQQQQQQQPQQQVFDHQQISSSISTMTQFGSVSQSLQTIPSFCRQQSFSDSNGIHMTNPIISPLHSLLGSFPQDESSQLLNLPRTHPMIHSSTWPSKRAAIDPHISSGNSQFVHLQEENMGTAQANISQNVSLPPFPGRECSLDQRNGDPQSNLLFGVNIEPSSLLMQNGMPNIRGICSDSDSTAIPFSSNYVNTAGTNFSANPAGTPSNCIEDSGILPNFVKVYKSGTFGRSLDISKFSSYHQLRSELAHKFNLEGELEDPLRSGWQLVFVDRENDVLLLGDDPWQEFVNSVWCIKILSPQEVQEMGRVDSYADRQESSRNLSSGITSVGSLEY

>CpARF33

MRLSAAGFSPQPPEGERRVLNSELWHACAGPLVSLPAVGTRVVYFPQGHSEQVAASTNKEVDAQIPNYPSLPPQLICQLHNLTMHADAETDEVYAQMTLQPLSAQELKEAYLPAELGSPSRQPTNYFCKTLTASDTSTHGGFSVPRRAAEKVFPPLDFSMQPPAQELIARDLHDNEWKFRHIFRGQPKRHLLTTGWSVFVSAKRLVAGDAVLFIWNEKNQLLLGIRRASRPQTVMPSSVLSSDSMHLGLLAAAAHAAATNSRFTIFFNPRASPSEFVIPLTKYVKAVYHTRVSVGMRFRMLFETEESSVRRYMGTITGISDLDPVRWQNSHWRSVKVGWDESTAGERQPRVSLWEIEPLTTFPMYPSPFPLRLKRPWPTGLPSFGIKDSDLGMNSPFMWLRGDNCLNFQGNGVSPWMQPRLDTSMMAMQPDMYQAMATAALQEMRAIDYSKIAPASVLQFQQSQGLPCQSSSLMQPQMMHQSQPQQAFLQSIQENQQHHLQPQLQPQQFNNQQQQPRQPQPPQLDHQQIPCTIPAAISQFASCSQSQLPSLQTIPSLCQQPSFSDSNCNPVTTTVSPLHSLAGSSSVQDESSQLLNLQRANSIIPSAGWPAKRAAIDPLTTGASQYFLPQGEILGTSQSSIPQNTVALPPFPGRECSNGDRDDGSDPQNHVLFGVNIESSSLLMQNGASHLRGVGNDSASTTLPFSSNYMSTAGTDFSVNPTMTSSNCIDESGFLQSHENVGQVNHPPNGTFVKVHKSGTYSRSLDITKFNSYPELRSELACMFGLEGELEDPLRSGWQLVFVDRENDVLLLGDGPWPEFVSSVWCIKILSPEEVHEMGKRGLELLNSVPIQRLSNSTCDDYGSRQDSRNLVSGITSVGPLDY
